# Lesion environments direct transplanted neural progenitors towards a wound repair astroglial phenotype in mice

**DOI:** 10.1038/s41467-022-33382-x

**Published:** 2022-09-28

**Authors:** T. M. O’Shea, Y. Ao, S. Wang, A. L. Wollenberg, J. H. Kim, R. A. Ramos Espinoza, A. Czechanski, L. G. Reinholdt, T. J. Deming, M. V. Sofroniew

**Affiliations:** 1 Department of Neurobiology, David Geffen School of Medicine, University of California, Los Angeles, CA 90095-1763 USA; 2 Department of Biomedical Engineering, Boston University, Boston, MA 02215-2407 USA; 3 Department of Chemistry and Biochemistry, University of California Los Angeles, Los Angeles, CA 90095-1600 USA; 4 Department of Bioengineering, University of California Los Angeles, Los Angeles, CA 90095-1600 USA; 5 The Jackson Laboratory, Bar Harbor, ME 04609 USA

**Keywords:** Regeneration and repair in the nervous system, Astrocyte

## Abstract

Neural progenitor cells (NPC) represent potential cell transplantation therapies for CNS injuries. To understand how lesion environments influence transplanted NPC fate in vivo, we derived NPC expressing a ribosomal protein-hemagglutinin tag (RiboTag) for transcriptional profiling of transplanted NPC. Here, we show that NPC grafted into uninjured mouse CNS generate cells that are transcriptionally similar to healthy astrocytes and oligodendrocyte lineages. In striking contrast, NPC transplanted into subacute CNS lesions after stroke or spinal cord injury in mice generate cells that share transcriptional, morphological and functional features with newly proliferated host astroglia that restrict inflammation and fibrosis and isolate lesions from adjacent viable neural tissue. Our findings reveal overlapping differentiation potentials of grafted NPC and proliferating host astrocytes; and show that in the absence of other interventions, non-cell autonomous cues in subacute CNS lesions direct the differentiation of grafted NPC towards a naturally occurring wound repair astroglial phenotype.

## Abstract (web summary)

Effects of lesion environments on transplanted neural progenitor cells (NPC) are not well characterized. Here, the authors show that NPC transplanted into CNS lesions generate cells that share transcriptional and functional features with host wound repair astroglia in mice.

## Introduction

Neural tissue that is lost to injury or disease in the mature mammalian central nervous system (CNS) is not spontaneously replaced. Instead, naturally occurring and conserved CNS wound repair mechanisms generate lesions in which non-neural lesion cores of fibrotic and inflammatory cells are partitioned from adjacent preserved neural tissue by newly formed astroglial borders^1–10^. Although this wound repair response is effective in clearing debris, limiting infection, and protecting nearby viable neural tissue, the resulting lesions often contain large volumes of non-neural fibrotic scar tissue that lacks the specialized neural cells necessary to support axon regeneration or the remodeling of neural circuits^10–14^.

Neural cell transplantation represents one potential therapeutic strategy for replacing lost neural tissue and improving outcome after CNS insults^15–18^. Different types of cell transplantation are being explored for this purpose, including fetal cell grafts^19–24^, adult neural progenitor cells (NPC)^25–27^, and NPC derived from lines of embryonic stem cells (ESC) or induced pluripotent stem cells (iPSC)^28–32^. Despite considerable progress in the derivation, production and transplantation of NPC into CNS injuries, many questions remain about the roles of cell autonomous versus non-cell autonomous factors in determining NPC differentiation and their neural repair support functions after grafting in vivo^33^.

Here, we examined how transplantation of NPC into different CNS environments altered their gene expression and differentiation fate in vivo. To do so we derived NPC from mouse embryonic stem cells (ESC) that constitutively express the ribosomal protein Rpl22 with a hemagglutinin (HA) tag (Rpl22-HA), also known as RiboTag, which permits cell-specific transcriptional profiling by RNA sequencing (RNAseq) and immunohistochemical characterization of HA-positive cells^34,35^. These RiboTag-NPC permit the selective transcriptomic analysis of NPC and their progeny after transplantation into host tissue in vivo even when the NPC are present in relatively small numbers compared with host cell numbers, and without the need for mechanical tissue dissociation and cell sorting that have the potential to induce transcriptional changes. We first extensively characterized these Rpl22-HA-expressing NPC (RiboTag-NPC), in vitro, including determining their transcriptional responses to factors known to induce different types of differentiation. We then used these well-characterized RiboTag-NPC to selectively evaluate NPC transcriptional profiles and differentiation fates following transplantation into uninjured CNS or into CNS lesions after forebrain stroke or spinal cord injury. We compared NPC transcriptional responses in vivo with NPC transcriptional responses in vitro to specific non-cell autonomous molecular cues that modified their differentiation; and we compared NPC differentiation phenotypes in vivo with the profiles of newly proliferated host astroglia that naturally adopt wound repair functions. We found that non-cell autonomous cues powerfully modify NPC transcription and can instruct different differentiation fates both in vitro and in vivo, and that grafted NPC are directed towards different cell fates by non-cell autonomous cues in uninjured or lesioned CNS tissue. Our findings reveal similarities between the transcriptional profiles, cellular morphologies, and certain functional features of cells derived from NPC transplanted into subacute CNS lesions and host astroglia that are stimulated by CNS injuries to proliferate and adopt a naturally occurring, border-forming wound repair astroglial phenotype.

## Results

### Neural induction and expansion of RiboTag mESC derives reproducible and stable NPC lines

**Fig. 1:**
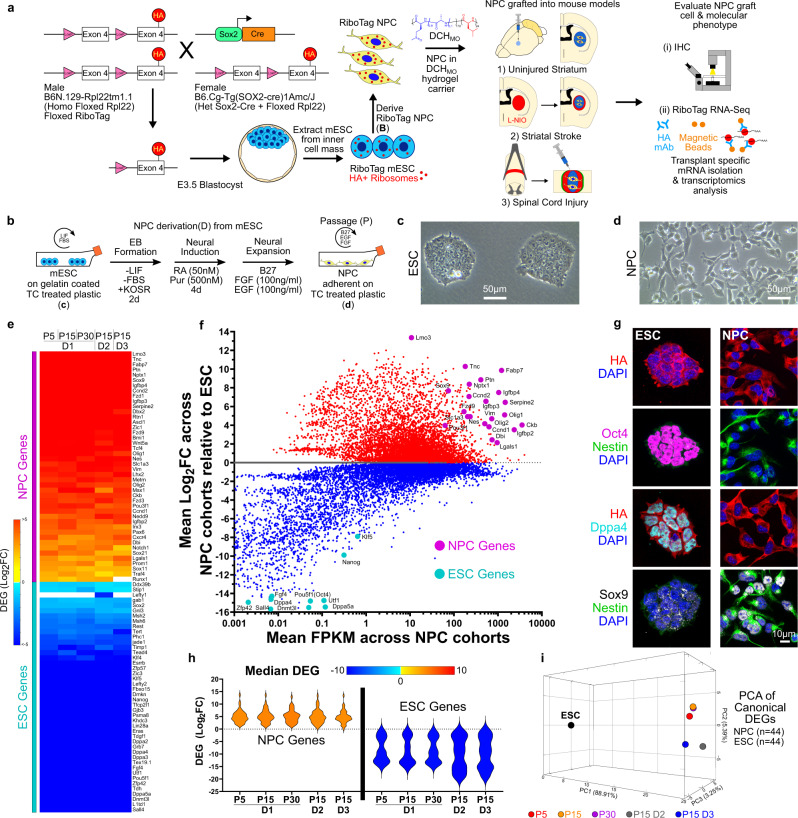
Neural induction and expansion of RiboTag mESC derives reproducible and stable NPC lines. **a** Schematic of RiboTag ESC derivation by Cre-Lox recombination and experimental workflow. **b** Schematic of NPC derivation by sequential neural induction and expansion. **c** Phase contrast image of ESC colonies. **d** Phase contrast image of adherent NPC. **e** Heatmap of DEGs for ESC and NPC gene panels for NPC at different Passages (P) and derivation (D). **f** M–A Plot of mean transcriptome differences averaged across NPC derivations (5 groups, *N* = 4 samples per group) referenced to ESC (*N* = 4 samples) showing increased expression of NPC genes and decreased expression of ESC genes. **g** Immunocytochemistry of ESC and NPC samples showing robust expression of HA-positive ribosomes across both cell types as well as downregulation of ESC markers (Oct4, Dppa4) and upregulation of neural stem markers (Nestin, Sox9) following application of neural induction and expansion procedures. **h** Violin plots of DEGs for the different NPC derivations (D) and passages (P) for the canonical NPC gene and ESC gene panels with color scale based on median DEG for each derivation. **i** 3D PCA of NPC samples from different derivations (D) and passages (P) using canonical NPC gene and ESC gene panels (44 genes for each panel).

Mouse ESC expressing RiboTag through Cre-Lox recombination (Fig. 1a) was used to generate NPC by neural induction and expansion^35,36^ (Fig. 1b). A single female mESC line that expressed RiboTag was used to generate NPC for all experiments in this study. Discrete multicellular ESC colonies (Fig. 1c) were transitioned into spindle-shaped NPC and expanded as adherent monolayer cultures rather than as floating neurospheres (Fig.1d). Transcriptome profiling by bulk RiboTag RNA Sequencing (RNA-Seq) showed gain of NPC phenotype and loss of ESC characteristics assessed using defined panels of canonical genes for each cell type^37^. NPC generation markedly reduced ESC gene expression with a median log_2_Fold Change (FC) of approximately −10 (Fig. 1e, f, h). Concurrently, NPC derivation induced increased expression of canonical neural stem cell genes with a median log_2_FC of approximately +5 (Fig. 1h). Loss of protein expression of ES markers Dppa4, Oct4, Nanog as well as emergence of NPC markers Nestin, Sox9 and Fabp7 by immunocytochemistry (ICC) and quantitative western blotting (WB) further supported successful NPC generation (Fig. 1g, Supplementary Fig. 1h, i). RiboTag-derived HA-positive ribosome expression was consistently and robustly detected in all cells across all in vitro evaluations including in mESC colonies, NPC derivations and differentiated NPC populations (Fig. 1g). To evaluate reproducibility and variability, three unique NPC derivations (D) were generated from different ESC cultures. To assess NPC stability and genetic drift, multiple passages (P) of a single NPC derivation up to passage 30 were generated. Samples from 5 unique NPC groups with different D and P combinations were profiled by bulk RNA-Seq (Fig. 1e, f, h, Supplementary Fig. 1a–g). Principal Component Analysis (PCA) of transcriptome differences showed minimal variation amongst NPC groups and comparably large differences between all NPC groups and the ESC state (Fig. 1i, Supplementary Fig. 1a, b). Two independent and complementary metrics, PCA Euclidean distance and Cosine Similarity (CS), showed high correlation amongst the different NPC groups but large and equivalent differences relative to ESC (Supplementary Fig 1c, e–g). Limiting comparisons to 88 DEGs comprising the canonical ESC and NPC gene panels strengthened the similarity amongst NPC groups compared with the full complement of 14,532 DEGs (Supplementary Fig 1e).

These data show that RiboTag-NPC derived from a common ESC stock by standard neural induction and expansion protocols are genetically stable and maintain unaltered RiboTag expression across serial passages and up to 4 months of constant culture with minimal differences between derivation or passage. RiboTag-NPC express well-established canonical neural stem cell genes and significantly downregulate all canonical embryonic stem cell genes making them suitable candidates for further investigation.

### RiboTag-NPC spontaneously differentiate into astrocyte and oligodendroglia lineage cells in vitro with phenotypes modulated by exposure to specific molecules

**Fig. 2:**
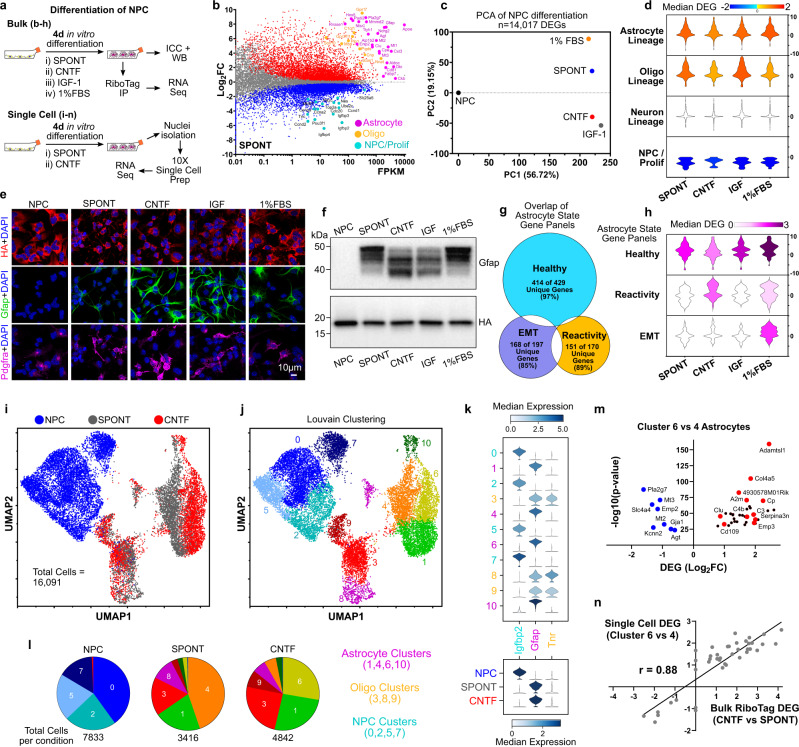
NPC cell autonomously differentiate into astrocyte and oligodendroglia lineage cells in vitro with phenotype modulated by specific molecules. **a** Schematic of experimental approach for evaluating in vitro NPC differentiation. **b** M–A Plot of Spontaneous (SPONT) differentiation referenced to NPC (*N* = 4 samples) showing increased expression of astrocyte and oligodendroglia lineage genes and decreased expression of NPC/proliferation genes. **c** PCA of all DEGs associated with NPC differentiation across the conditions referenced to NPC showing large differences for all differentiation conditions compared to the NPC state and variation amongst the unique differentiation conditions. **d** Violin plots of DEGs for neural cell-specific gene panels. The color of violin plot reflects the value of the median DEG for specified gene panel. **e** ICC staining of the differentiation conditions for astrocyte (Gfap) and oligodendroglia (Pdgfra) markers. All DAPI-positive cells across differentiation conditions are RiboTag (HA) positive. **f** Western blot for Gfap and HA showing variation in Gfap isoform bands amongst the various differentiation conditions. HA expression is not altered by differentiation condition. **g** Venn diagram of genes for the different astrocyte states derived through meta-analysis of published datasets showing minimal overlap of healthy astrocyte, reactivity and EMT genes. **h** Violin plots of DEGs from the healthy astrocyte, reactivity, and EMT gene panels that are amongst the top 2000 most differentially expressed genes for the differentiation conditions. The color of violin plot reflects the Median DEG for specified gene panel. **i** UMAP of 16,091 single-nuclei samples designated by NPC, SPONT, and CNTF condition and derived from the top 5000 uniquely expressed genes. **j** UMAP of 16,091 single-nuclei samples as in **i**) segregated by Louvain clustering algorithms into 11 unique clusters. **k** Violin plots of expression values for Igfbp2, Gfap and Tnr for the 11 unique clusters. Igfbp2 is highly expressed in NPC dominant clusters. Gfap is highly expressed in astrocyte clusters. Tnr is highly expressed in oligodendroglia lineage cell clusters. SPONT and CNTF conditions result in predominant astrocyte differentiation. **l** Pie charts for NPC, SPONT and CNTF conditions indicating the predominate cell populations for each condition. Cluster 0 (NPC) predominates in NPC sample. Astrocyte Cluster 4 predominates in SPONT condition whereas Cluster 6 Astrocytes are the dominant cell type in CNTF condition. **m** Volcano plot of DEGs identified by comparing Cluster 6 vs Cluster 4 astrocytes. Cluster 6 astrocytes upregulate numerous genes on the reactivity gene panel (red labeled markers) and downregulate several canonical healthy astrocyte genes relative to Cluster 4 cells (blue labeled markers). *p* values for DEGs determined by pairwise *t*-test (two-sided) with overestimated variance. **n** Correlation analysis of Single-nuclei RNA-Seq DEGs for Cluster 6 vs 4 astrocytes compared with bulk RiboTag DEGs for CNTF compared with SPONT. The DEGs identified by both methods show strong correlation (*r* = 0.88).

RiboTag-NPC maintained in serum-free medium with mitogens EGF and FGF retain multipotency over serial passages, and as expected^38^ undergo spontaneous, cell autonomously regulated differentiation (SPONT) over 4 days under reduced EGF/FGF conditions (Fig. 2a) which induced upregulation of astroglial and oligodendroglia lineage genes and downregulation of neural stem and proliferation genes (Fig. 2b). We examined the effects of different well known extracellular stimuli on NPC gene expression and differentiation in vitro so as to enable comparisons with changes in phenotype and gene expression undergone by NPC transplanted in vivo. We added CNTF or 1% FBS to promote astrocyte differentiation^36,39,40^; and IGF-1 to promote oligodendroglia differentiation^41^. We compared variations in transcription profiles using reference gene panels for Astrocyte lineage, Oligodendrocyte lineage, and Neuronal lineage cells (Supplementary Table 1). The healthy astrocyte gene panel was derived by performing meta-analysis of eight unique archival datasets of astrocyte-specific gene expression and contained 429 genes that were identified to be astrocyte enriched genes in at least 5 of the 8 datasets (Supplementary Fig 2). Oligodendrocyte lineage and neuronal gene panels were derived using PanglaoDB single-cell datasets^42^. PCA and CS analysis of all 14,017 DEGs (Fig. 2c) or neural cell type-specific DEGs (Supplementary Fig. 3a, b) revealed large differences for all differentiated cells compared to the NPC state (defined by PC1) and smaller but measurable variation among the four differentiation conditions (defined by PC2). Astroglia and oligodendroglia lineage phenotypes emerged as the dominant transcriptomic signatures amongst all differentiation conditions, with little overall change in neuronal genes but comprehensive downregulation of NPC and proliferation genes (Fig. 2d). For many astrocyte genes (including *Gfap*, *Apoe, Pla2g7, Slc1a3, Sparc, Ndrg2, Id3*), the expression levels (by FPKM) after in vitro differentiation were comparable to the levels detected for healthy mouse spinal cord astrocytes in vivo where RNA was recovered using transgene-derived RiboTag-HA expressed via Gfap-Cre (Supplementary Table 2). However, other astrocyte genes (*Slc7a10, Myoc, Fam107a, Gjb6, Slc1a2, Aqp4, Aldh1a1, Slc2a4, S100b, Atp1a2*), were not expressed by astroglia differentiated from NPC at in vivo astrocyte levels but were generally increased compared to NPC. IGF-1 evoked the highest increase in oligodendrocyte gene expression (Fig. 2d, Supplementary Fig. 3a).

Gfap-positive astrocytes and Pdgfra-positive oligodendroglia were detected across all differentiation conditions by ICC, but expression levels and morphology patterns varied markedly (Fig. 2e). Variation in Gfap-positive cell morphology amongst differentiation conditions was associated with differences in Gfap isoform usage^43^ demonstrated by WB staining bands to mouse monoclonal and rabbit polyclonal Gfap antibodies (Fig. 2f). Lower molecular weight bands for Gfap were predominant in CNTF and IGF-1 conditions and this correlated with the observation of long filamentous morphologies by ICC under the same conditions (Fig. 2e). Higher molecular weight Gfap bands were more concentrated in SPONT and 1% FBS and correlated with weaker Gfap staining by ICC (Fig. 2e, f). Despite displaying similar Gfap bands, 1% FBS treated cells were flat and polygonal shaped whereas SPONT had long filamentous morphologies (Fig. 2e, f). RiboTag expression was maintained in every cell across all differentiation conditions (Fig. 2e, f). Some, but very few, Tuj-1 positive cells were detected in differentiation conditions but often colocalized with Gfap (Supplementary Fig. 3c).

To identify transcriptome differences amongst the four differentiation conditions that may be associated with the varied astrocyte cell morphologies identified by ICC, we analyzed the top 2000 most differently regulated DEGs which mathematically are the 2000 DEGs with the greatest variation across the four differentiation conditions. To profile transcriptome differences in astrocyte phenotype amongst the differentiation conditions we used the healthy astrocyte gene panel as before (Supplementary Table 1, Supplementary Fig. 2) and in addition generated gene panels for two other broad categorizations of astrocyte states: (i) a reactive astrocyte gene panel that was derived from meta-analysis of published acutely injured astrocyte transcriptomics data (Supplementary Fig. 4); and (ii) an EMT-like (epithelial to mesenchymal transition) gene panel that was adapted from Msigdb gene sets which has previously been applied by others to evaluate astrocytes responding to CNS injury^44,45^ (Supplementary Table 3). EMT-related genes were examined also because pro-regenerative zebrafish glia activate an EMT-like transcriptional program that is required for glial-bridging of axons and distinguishes zebrafish and mammalian glia after CNS injury^46^. There was minimal overlap of genes on these three astrocyte state gene panels with each gene panel having at least 85% unique genes (Fig. 2g). The top 2000 most different DEGs for the in vitro differentiation conditions, included genes from the Healthy (78 out of 429), Reactivity (40 out of 170) and EMT (49 out of 197) astrocyte state gene panels. All 4 conditions showed upregulation of various different healthy astrocyte genes compared with NPC, but only CNTF and 1% FBS evoked net upregulation of reactivity genes, with CNTF causing the largest upregulation (Fig. 2h, Supplementary Fig 2d). Only 1% FBS exposure had a net upregulation of EMT genes. Consistent with these transcriptional differences, CNTF and FBS conditions stained positively for astrocyte reactivity markers Cd44 and Vimentin whereas SPONT did not (Supplementary Fig. 2e).

To evaluate the relative proportions and phenotypes of astrocyte and oligodendrocyte lineage cells, we performed single-nucleus RNA-Seq on NPC, SPONT, and CNTF-directed differentiation in vitro. 16,091 total nuclei from the 3 conditions were separated into 11 clusters by Louvain algorithms (Fig. 2i, j, Supplementary Fig. 2f, g). NPC separated into 4 clusters defined by shared elevated expression of *Igfbp2*, a canonical neural stem cell gene^47^ and other canonical NPC genes (Fig. 2k, Supplementary Fig. 2h), with variation in cell cycle/proliferation gene expression prompting the 4 cluster segregation (Supplementary Fig. 4i). SPONT and CNTF conditions induced comprehensive differentiation with only 0.32% and 0.29% of SPONT and CNTF treated cells partitioning into NPC clusters (Supplementary Fig. 2f, g). SPONT and CNTF conditions generated oligodendrocyte (*Tnr* high) and astrocyte (*Gfap high*) lineage cells (Fig. 2k, l, Supplementary Fig. 2g, j) but no obvious neuronal populations. Oligodendrocyte lineage cells segregated into 3 clusters: (i) oligodendrocyte progenitor cells (OPCs) (Cluster 3), (ii) mature oligodendrocytes (Cluster 8), and (iii) a smaller number of actively proliferating OPCs (Cluster 9), and SPONT and CNTF promoted comparable distributions of these different oligodendrocyte clusters (Fig. 2j, k, Supplementary Fig. 2k, l). Astrocyte lineage cells partitioned into four clusters: immature astrocytes (Cluster 1), and more mature astrocytes (clusters 4, 6 and 10) (Supplementary Fig. 2m). SPONT and CNTF promoted different predominant astrocyte clusters such that SPONT comprised ~90% of total cluster 4 and CNTF comprised ~93% of total cluster 6 astrocytes (Fig. 2i, Supplementary Fig. 4f, k). Notably, cluster 6 astrocytes, when compared with cluster 4 astrocytes, exhibited higher expression of diverse reactivity genes from the meta-analysis curated reactivity gene list such as *Emp3, Serpina3n*, and *C4b* (Fig. 2m, Supplementary Fig. 2m) and cluster 4 astrocytes exhibited comparatively higher expression of canonical healthy astrocyte genes *such as Pla2g7, Mt 2,3, Slc4a4, Kcnn2, Agt, and Emp2*. Cluster 6 was also enriched for other genes linked to astrocyte reactive states including *C3, Clu, Cp*, and *Cd109* (Fig. 2m). Moreover, there was strong correlation (*r* = 0.88) between the DEGs for cluster 6 referenced to cluster 4 astrocytes and the Bulk RiboTag DEGs for CNTF relative to SPONT suggesting that the signature that emerged in the bulk analysis was likely associated with the difference in the predominant astrocyte cell population and not just a global upregulation of those reactivity genes across all cells (Fig. 2n).

These data show that upon mitogen removal, RiboTag-NPC cell autonomously differentiate in vitro into a mixed population of astrocyte and oligodendrocyte lineage cells, with few neuronal lineage cells, and that exposure to non-cell autonomous molecular factors alters the transcriptome phenotypes in a manner consistent with previous observations^44,45,48–50^. While the expression of astrocyte specific genes in differentiated samples was upregulated compared to NPC, the levels of expression of these genes by FPKM generally did not reach the levels observed in samples of host spinal cord astrocytes recovered using the same RiboTag procedure, and certain specialized astrocyte genes associated with maintaining neuronal functions in healthy neural tissue were not significantly expressed in cultures.

### High serum concentration induces myofibroblast-like phenotypes in NPC in vitro

NPC transplanted into CNS lesions are exposed to lesion-derived molecular cues from multiple sources that can alter cell fate^33^. Acute CNS lesions caused by ischemic stroke or hemorrhagic trauma have substantial blood-brain barrier leak creating microenvironments with spatially varied concentrations of blood and serum-derived molecules^51^. To make comparisons with NPC transplanted into CNS lesion in vivo, we evaluated the effects on NPC phenotype and transcriptome of increasing serum concentrations during spontaneous differentiation in vitro. Fetal bovine serum (FBS), a widely used media supplement known to influence astrocyte phenotypes in vitro compared to serum-free media^49,50,52^, induced concentration-dependent changes in the transcriptome profiles of NPC undergoing spontaneous differentiation after mitogen withdrawal (Supplementary Fig. 5a, b). Notably, PCA on 10,427 DEGs revealed increasing divergence from the SPONT state for higher concentrations of FBS and analysis of astrocyte state gene panels (healthy, reactivity, and EMT) revealed that FBS concentration-dependent effects involved adoption of new cell fates rather than attenuation of NPC differentiation, such that a low dose (1%) induced the most pronounced astrocyte phenotype, and increasing serum concentrations evoked myofibroblast-like phenotypes often associated with EMT, including pronounced upregulation of α-smooth muscle actin (α-Sma) together with a concurrent decrease in healthy astrocyte genes (Supplementary Figs. 5b–e and 6a–d). FBS concentration-dependent changes in NPC differentiation were also identified by ICC and WB such that Gfap protein expression decreased at higher FBS concentrations, while α-Sma and F-actin (Phalloidin) markedly increased and cells exhibited increases in cytoplasm and nuclei size, with a flattened morphology and augmented arrangement of actin stress fibers, in a manner consistent with a contractile activated myofibroblast-like state (Supplementary Figs. 5e, g and 6d)^53^. We also found that removing high molecular weight molecules from FBS via 100 kDa molecular weight cut off (MWCO) ultrafiltration attenuated α-Sma expression and increased Gfap expression (Supplementary Figs. 5h and 6e, f). Treating cells with only the higher molecular weight components of FBS retained after ultracentrifugation induced α-Sma expression comparable to that for the equivalent concentration of FBS (Supplementary Figs. 5h and 6e, f). Treating NPC with an established EMT inhibitor, the Tgf-β receptor inhibitor SB-431542^54^, significantly reduced α-Sma expression at 10% FBS suggesting that FBS-induced EMT may involve this pathway (Supplementary Fig. 5i)^55^. CNTF combined with FBS also attenuated the upregulation of EMT genes and α-Sma protein expression while sustaining the full complement of Gfap isoforms including isoforms induced uniquely by FBS or CNTF alone (Supplementary Fig. 6g, h). We also treated NPC with freshly prepared mouse serum, which in high concentrations induced a comparable phenotype to FBS, including reduction in Gfap expression, increased cytoplasm and nuclei size, flattened morphology, and increased arrangement of actin stress fibers (Supplementary Figs. 5j and 6i), ruling out non-specific effects of FBS.

These data suggest that serum-derived factors in CNS lesions have the potential to non-cell autonomously modify NPC in a concentration-dependent manner such that low serum concentrations may promote astrocyte differentiation, whereas high concentrations may drive NPC towards myofibroblast-like phenotypes via EMT-like processes. This effect was associated with proteins in the serum fraction with molecular weights greater than 100 kDa that includes candidate molecules such as globulins, transferrin, complement proteins and assembled lipoproteins.

### RiboTag-NPC grafted into healthy mouse striatum differentiate into astrocyte and oligodendroglia lineage cells

**Fig. 3:**
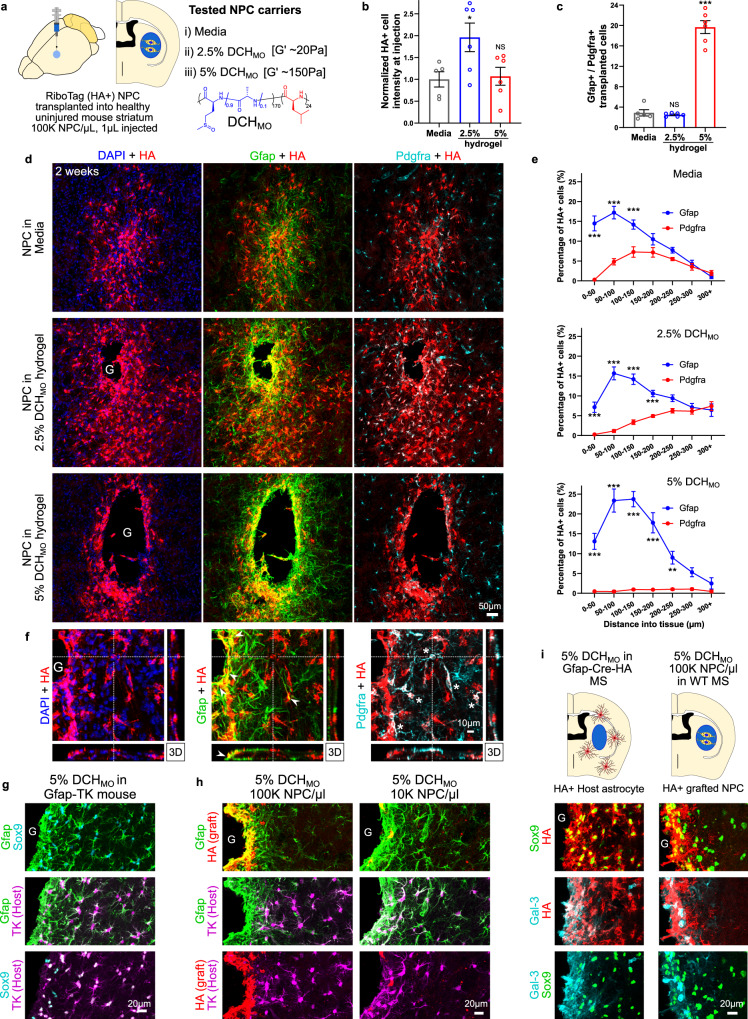
NPC grafted into healthy mouse striatum differentiate into astrocyte and oligodendroglia lineage cells in a spatial and carrier-dependent manner. **a** Schematic summarizing experimental approach for evaluating NPC grafting in uninjured mouse striatum and evaluating different hydrogel (DCH_MO_) transplant carrier formulations. **b** Quantification of normalized NPC number at striatal injection site by HA-positive cell intensity on IHC sections. (*N* = 5 for Media, *N* = 6 for hydrogels). *p value < 0.05, NS not significant. One-way ANOVA with Tukey multiple comparison test. **c** Quantifi**c**ation of proportions of Gfap-positive to Pdgfra-positive (Gfap+/Pdgfra+ ratio) transplanted HA-positive cells (*N* = 5 for Media, *N* = 6 for hydrogels). ***p value < 0.0001, NS not significant. One-way ANOVA with Tukey multiple comparison test. **d** Survey Immunohistochemistry (IHC) images of grafted HA-positive NPC in healthy striatum stained for astrocytes (Gfap) and oligodendroglia lineage (Pdgfra) at 2 weeks after NPC transplantation using media or hydrogel carriers. Hydrogel deposits (G) lack fluorescence and therefore appear as black spaces on IHC. Most, but not all NPC have migrated out of hydrogel deposits. **e** Quantification of the percentage of Gfap- and Pdgfra-positive grafted cells as a function of distance from the edge of detectable tissue adjacent to the hydrogel deposits for specific distances into neural parenchymal tissue for each carrier (*N* = 5 for Media, *N* = 6 for hydrogels). ***p value < 0.0001, **p value < 0.002. Two-way ANOVA with Bonferroni multiple comparison test. **f** High magnification 3D view showing HA-positive grafted NPC in 2.5% DCH_MO_ hydrogel (G) carrier showing formation of Gfap-positive borders (➤) and Pdgfra-positive cells in neural parenchyma (*) by grafted NPC. **g** Detailed IHC images showing the contributions of host astrocytes (TK-positive) to astrocyte borders formed at hydrogel-neural tissue interface for hydrogel injection alone. **h** Detailed IHC images showing the contributions of host astrocytes (TK-positive) and grafted NPC (HA) to the astrocyte border formed at the NPC hydrogel carrier-neural tissue interface. Contribution from NPC to the astrocyte border is dependent on concentration of cells grafted. **i** Schematic and detailed IHC images comparing HA-positive cell responses from host or NPC graft to hydrogel injections. HA-positive host astrocytes derived using Gfap-Cre-HA transgenic mice show prominent HA-positive borders expressing Sox9 and Galectin-3 (Gal-3) at hydrogels injected without NPC. Transplanting HA-positive NPC using the same hydrogel carrier provokes astrocyte border formation by grafted NPC and comparable expression of Sox9 and Galectin-3 (Gal-3). All graphs are mean ± s.e.m.

We next examined how different tissue environments influence transplanted NPC fate in vivo by using the RiboTag-NPC characterized above and conducting immunohistochemical and transcriptional analysis of NPC grafted either into healthy or injured tissue. We began by comparing the effects of different potential grafting vehicles on the survival and appearance of RiboTag-NPC transplanted into healthy mouse striatum and identified by immunohistochemistry for HA. Because biomaterial vehicles represent potential means of improving NPC transplantation^56^, we tested two different formulations of our previously developed synthetic polypeptide hydrogel (DCH_MO_) that differed in polymer weight fraction and mechanical stiffness^35^, and compared these with grafting in media only (Fig. 3a). Cells prepared in media at a density of 100 K cells/µL showed an increased tendency to clump and separate in the pipette over the course of an injection session whereas both hydrogel groups maintained uniform suspensions of cells throughout injections. NPC transplanted in media showed reduced cell survival compared to the 2.5% hydrogel but comparable survival numbers to the 5% hydrogel (Fig. 3b).

We found that grafting vehicle properties markedly influenced the spatial distribution and differentiation phenotype of transplanted NPC (Fig. 3c, d). Hydrogel carriers augmented the dispersal of grafted cells over a larger tissue volume compared to media and this effect was influenced by hydrogel formulation (Supplementary Fig. 7a, b). However, for all experimental groups the majority of grafted NPC and progeny persisted in neural tissue within 350 µm radially from the center of the striatal injection (Fig. 3d, e, Supplementary Fig. 7a–e). We used Gfap and Pdgfra immunohistochemistry to identify HA-positive astrocyte and oligodendrocyte lineage cells derived from NPC, respectively (Fig. 3f, Supplementary Fig. 7g). Pdgfra is a well-characterized marker for oligodendrocyte progenitor cells (OPC)^57^. For all conditions, NPC differentiated into a mix of Gfap and Pdgfra-positive cells at 2 weeks after transplantation. Notably, graft-derived progeny residing closest to the injection epicenter predominately expressed Gfap whereas Pdgfra-positive cells were more prevalent in neural tissue greater than 300 µm from the injection center (Fig. 3d, Supplementary Fig. 7c, d, f). The 2.5% hydrogel and media showed an overall number ratio of Gfap/Pdgfra-positive cells of between 2.5 and 2.9, and this ratio was similar to the proportions of astrocyte and oligodendrocyte lineage cells characterized by single-cell transcriptome analysis of spontaneous NPC differentiation in vitro. In contrast, the stiffer 5% hydrogel carrier is slowly degraded and persisted longer in the tissue (Fig. 3d), and NPC grafted in 5% hydrogel showed a predominant Gfap-positive cell phenotype with a Gfap/Pdgfra-positive cells ratio of ~20 (Fig. 3c). No neuronal differentiation was observed in any animals across the different transplant groups, although grafted NPC interacted directly with healthy neurons (Supplementary Fig. 7h).

Graft-derived astrocyte or OPC-like cells that had migrated into neural tissue appeared dispersed with individual cell domains reminiscent of tiled host glia (Fig. 3e, Supplementary Fig. 7g), whereas NPC at the injection site adopted a densely packed border-forming astrocyte phenotype with overlapping processes (Fig. 3d, f) similar to host border-forming astrocytes at interfaces between host neural tissue and biomaterial depots^1^ (Fig. 3g). This border-forming glia limitans-like phenotype was most apparent around the 5% hydrogel, which formed larger and stiffer deposits that evoked a stronger foreign body response^1^. Notably, when transplanted in sufficiently high numbers, grafted cells qualitatively appeared to comprise the majority of these border-forming astroglia as demonstrated through comparisons in Gfap-TK transgenic reporter mice with labeled host astrocytes (Fig. 3h). When grafted at low NPC concentrations, host cells predominated at these borders and the sparser number of grafted cells intermingled with them (Fig. 3h). In addition, grafted cells forming these astrocyte limitans borders expressed molecular markers, Gfap, Sox9, galectin-3 (Gal-3) and fibronectin (Fn), similar to host border-forming astrocytes (Fig. 3f–i, Supplementary Fig. 7i).

To assess effects of the injection procedure on NPC fate, we performed RiboTag RNA-Seq analysis on (i) NPC passed through glass pipettes in media or 2.5% hydrogel, and (ii) cells allowed to dwell in hydrogel for 4 h before injection, the maximum potential time course of an injection session (Supplementary Fig. 8a). Compared to NPC in culture, there were small but detectable differences in the transcriptome for samples passed through pipettes and most of these changes were shared between these three conditions (i.e. injection dependent but carrier and time independent) (Supplementary Fig. 8b, c). Comparisons between hydrogel and media carriers identified a modest increase in astrocyte differentiation transcriptome signature and a concurrent decrease in some progenitor gene expression in the hydrogel group that increased over the 4-h dwell period in pipettes (Supplementary Fig. 8d, e).

These findings show that grafted NPC that migrate into healthy CNS tissue generate cells with phenotypic features similar to surrounding healthy glia, whereas NPC immediately abutting injection site wounds adopt features similar to host border-forming astroglia. These data also show that hydrogel carrier formulation and mechanical properties act as non-cell autonomous modifying cues that influenced survival and differentiation outcomes of transplanted NPC. The mechanically stiffer hydrogel stimulated a predominant glia limitans-like border phenotype consistent with transplanted NPC responding to the hydrogel as a foreign body and contributing to host astroglial border formation associated with the classic CNS foreign body response^1^. The hydrogel carrier with lower mechanical stiffness but comparable chemistry showed the best NPC survival and interestingly, using this carrier the relative proportions of astrocyte and oligodendrocyte lineage cells detected after NPC grafting into healthy, uninjured tissue were similar to the proportions identified by single-nucleus transcriptome analysis of spontaneous NPC differentiation in vitro. This optimal 2.5% hydrogel formulation was used for subsequent experiments.

### NPC grafted into striatal stroke or SCI lesions generate astroglia that reduce fibrotic scar and bridge lesions and resemble host-derived border-forming astroglia

**Fig. 4:**
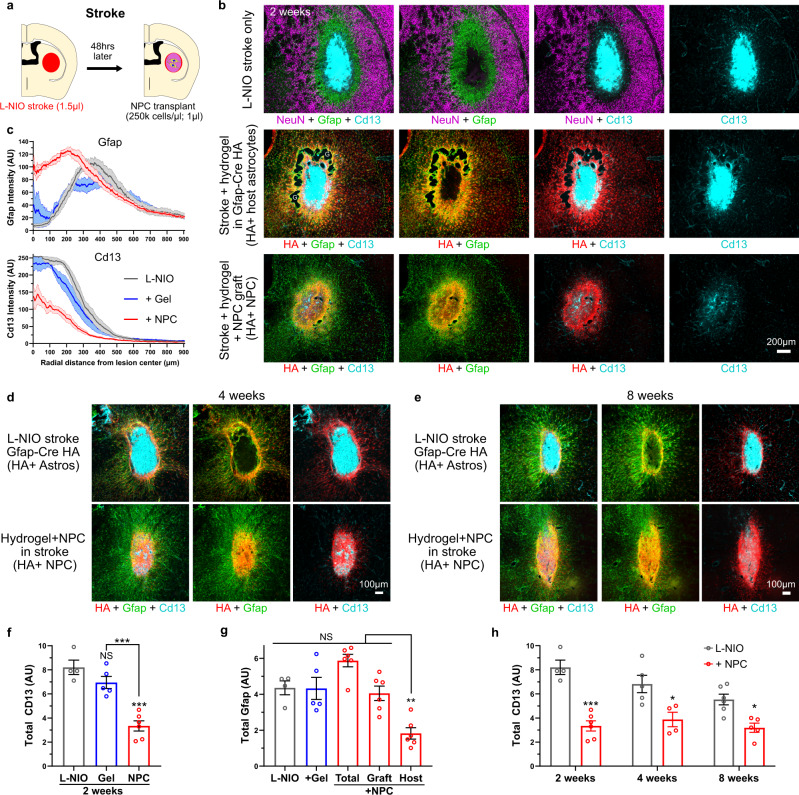
NPC grafted into striatal stroke generate astroglia that reduce fibrotic scar and bridge lesions. **a** Schematic summarizing experimental approach for evaluating NPC grafting in mouse striatal stroke. **b** Survey IIHC images of stroke lesions at 2 weeks comparing effect of hydrogel (DCH_MO_) alone or grafted HA-positive NPC on stroke lesion phenotype. NPC grafted lesions show Gfap-positive lesions with reduced Cd13-positive tissue in non-neural lesion core. **c** Quantification of Gfap and Cd13 intensity at stroke lesions as a function of the radial distanced from the center of the lesion with mean±s.e.m. (s.e.m represented as shaded region). **d**, **e** Survey IHC images of stroke lesions at 4 weeks (**d**) and 8 weeks (**e**) comparing the effect of grafted HA-positive NPC on stroke lesion phenotype. **f** Quantification of total Cd13 at stroke lesion cores at 2 weeks after L-NIO injection for stroke only, hydrogel (DCH_MO_) alone, and NPC grafting by performing area under the curve (AUC) calculations of Cd13 traces in c. (*N* = 4 for L-NIO only, *N* = 5 for gel, *N* = 6 NPC graft). ***p value < 0.0005. One-way ANOVA with Tukey multiple comparison test. **g** Quantification of total Gfap at stroke lesions at 2 weeks after L-NIO injection for stroke only, hydrogel (DCH_MO_) alone, and NPC grafting by performing AUC calculations of Gfap traces in c. (*N* = 4 for L-NIO only, *N* = 5 for gel, *N* = 6 NPC graft) Using Gfap-HA colocalization (see Supplementary Fig. 11) the total Gfap contribution from the NPC graft and host astrocytes in the NPC graft group was determined. **h** Quantification of total Cd13 at stroke lesion cores at 2, 4, and 8 weeks after L-NIO injection for stroke only and NPC grafting. (*N* = 4, 5, 6 for L-NIO only at 2, 4, and 8 weeks, *N* = 6, 4, 5 for NPC grafts at 2, 4, and 8 weeks). ***p value < 0.0001, **p value < 0.005, *p value < 0.05. Two-way ANOVA with Tukey multiple comparison test. All graphs are mean ± s.e.m. AU arbitrary units.

**Fig. 5:**
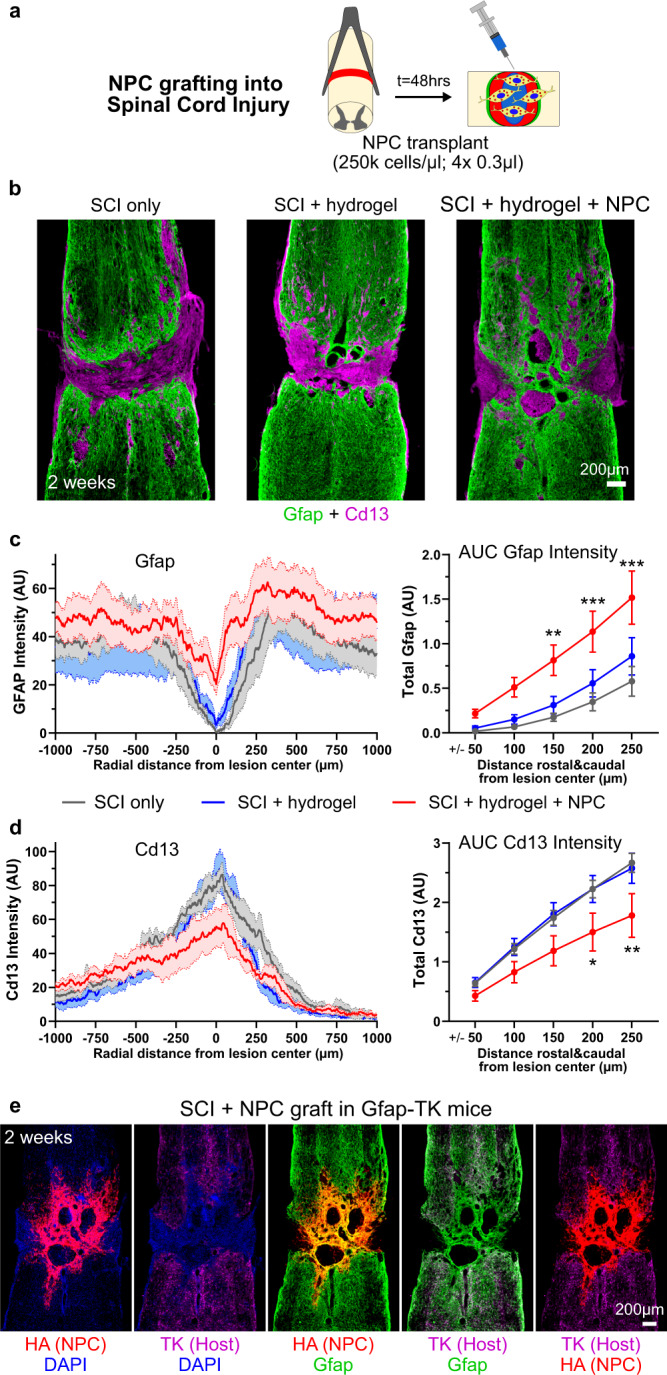
NPC grafted into SCI generate astroglia that reduce fibrotic scar and bridge lesions. **a** Schematic summarizing experimental approach for evaluating NPC grafting in mouse spinal cord injury (SCI). **b** Survey IHC images of SCI lesions at 2 weeks comparing effect of hydrogel alone or grafted HA-positive NPC on SCI phenotype. NPC grafted lesions show Gfap-positive bridges with reduced Cd13-positive tissue in lesion cores. **c** Gfap intensity plots and quantification of total Gfap via an area under the curve (AUC) calculation of Gfap Intensity within defined regions at SCI lesion cores at 2 weeks showing increased Gfap at lesion cores with NPC grafts. **d** Cd13 intensity plots and quantification of total Cd13 via an area under the curve (AUC) calculation of Cd13 Intensity within defined regions at SCI lesion cores at 2 weeks showing decreased Cd13 at lesion cores with NPC grafts. For both **c**, **d**, *N* = 6 for SCI only, *N* = 5 for gel, *N* = 6 NPC graft, ***p value < 0.0005, **p value < 0.006, *p value < 0.05. Two-way ANOVA with Tukey multiple comparison test. Gfap and Cd13 intensity plots show mean as darken lines and SEM as shaded area. AUC graphs are mean ± s.e.m. **e** Survey IHC images of NPC grafted SCI lesions at 2 weeks in GFAP-TK mice showing contribution of grafted NPC to the Gfap-positive tissue at the lesion core. AU arbitrary units.

We next used immunohistochemistry for HA to examine NPC transplanted into either L-NIO-induced stroke lesions in the striatum or into crush spinal cord injury (SCI) (Figs. 4 and 5). These models generate two common types of CNS lesions, ischemic lesions due to loss of blood flow after stroke or hemorrhagic lesions caused by severe traumatic injury after crush SCI. NPC in 2.5% hydrogel carrier were grafted at two days after either injury (Figs. 4a and 5a), a timepoint after acute CNS injuries when most neural cell death has occurred and multicellular repair processes such as debris clearance and glial proliferation have begun, including the onset of host astrocyte proliferation to form borders that separate fibrotic scar from adjacent viable neural tissue (Supplementary Fig. 9)^4,10,58^. Using separate cohorts of mice, we compared host-astrocytes transgenically labeled with RiboTag via Gfap-Cre, with grafted RiboTag-NPC (Fig. 4, Supplementary Fig. 10b–e). Host astrocytes expressing transgene-derived RiboTag-HA under Gfap-Cre were readily detected in borders around stroke lesions by IHC staining for HA (Fig. 4b), indicating that the HA expressed by NPC-derived cells should also be detectable via HA IHC and that death of HA-positive cells does not result in non-specific HA deposits.

At two days after L-NIO injection, untreated stroke lesion cores exhibited complete loss of viable neurons and glia, accumulation of axon damage markers including amyloid precursor protein (App), pronounced BBB leak, and onset of acute inflammation (Supplementary Fig. 9a–e). App breakdown into Aβ demarcated the nascent lesion border at this timepoint, suggesting a potential role for Aβ in normal wound responses (Supplementary Fig. 9b, c). NPC grafts were placed directly into the stroke lesions using stereotactic injections and tissue was harvested after 2, 4 and 8 weeks (Fig. 4b–e). By 2 weeks, untreated L-NIO strokes formed focal compartmentalized lesions with a dense core of Cd13-postive non-neural cells consisting mainly of infiltrating macrophages and fibrotic scar^1^ that was surrounded by a border of newly proliferated astrocytes, which isolated the lesion from immediately adjacent viable neural tissue^1^ (Fig. 4b). Injection of empty DCH_MO_ hydrogel into stroke lesions caused no significant alteration to this lesion phenotype (Fig. 4b). In contrast, NPC grafts survived robustly, localized at the lesion site and markedly altered the cellular organization of stroke lesions such that by two weeks, HA-positive grafted cells that were strongly Gfap-positive had consistently spread throughout the entire lesion core (Fig. 4b, g). Lesion remodeling facilitated by NPC grafts resulted in significant reduction in total number of Cd13-positive cells and fibrotic lesion size (Fig. 4b–e) in a manner similar to the anti-inflammatory and lesion-restricting roles performed by host reactive astrocytes^5,10,59^. Grafted HA-positive cells persisted robustly for the duration of all experiments and were abundantly present at 4 and 8 weeks after grafting resulting in a long lasting reduction of infiltrating Cd13-positive cells (Fig. 4d, e, h).

Given the reduction in Cd13-positive fibrotic and inflammatory tissue within lesion cores that received NPC grafts (Fig. 5b–h), we examined more closely the spared neural parenchyma and lesion core interface to investigate possible neural cell infiltration promoted by grafts. Spared neural tissue was defined as containing at least 4 main types of host neural cells: NeuN-positive and neurofilament-positive neurons, Aldh1l1-positive astrocytes, Pdgfra-positive OPC and P2yr12-positive microglia (Fig. 4b, d, e; Supplementary Figs. 10 and 11). In untreated strokes, these four neural cell types were all absent from the lesion core, with newly proliferated Aldh1l1-positive astrocytes and P2yr12-positive microglia forming sharp borders at the interface between lesion core and spared neural tissue, which persisted at least 8 weeks after injury, the longest timepoint examined (Supplementary Fig. 10). Stroke lesions treated with NPC grafts showed a comparable volume of lesion core that lacked neurons but was packed with Gfap-positive graft-derived cells throughout its entire volume and exhibited a less sharply defined border at its interface with spared neural tissue (Supplementary Fig. 10). NPC grafted strokes showed a measurable reduction in host Gfap-positive cells at lesions borders but not an overall reduction in total Gfap due to the contributions from graft-derived Gfap-positive NPC (Fig. 4g). At 2 weeks, these NPC-derived Gfap-positive cells exhibited negligible expression of the mature astrocyte markers Aldh1l1 (Supplementary Fig. 10a), but this expression increased by 4 and 8 weeks, particularly along the immediate interface with host neural tissue (Supplementary Fig. 10b). NPC treated stroke lesions transiently contained P2yr12-positive CNS derived microglia at 2 weeks, but not at 4 or 8 weeks. At these later times, Cd68-postive, P2yr12-negative cells were present in NPC grafted lesions, but these cells were at a visibly lower density compared to the untreated stroke suggesting an NPC graft induced shift in the immune cell milieu within the lesion core (Supplementary Fig. 10d). Lesion cores with NPC grafts did not show any neuronal repopulation or ingrowth of neurofilament-positive axons by 8 weeks (Supplementary Fig. 10e).

Even though we used precise stereotactic injections to introduce grafts into stroke lesions, we found it notable that grafted NPC were consistently localized to stroke lesion cores such that across all grafted animals at least 95% of grafted NPC persisted within the lesion core within an average radius of approximately 400 µm (Supplementary Fig 11a, b). It seems unlikely that such precise localization could be attributed only to surgical technique and may have involved some migration and potential chemoattraction of grafted NPC to the stroke lesion cores, given the known migratory potential of certain NPC stages^60^. Grafted NPC that localized within this 400 µm radial lesion core zone almost exclusively assumed border-forming astroglia roles as evident by over 81% of these cells expressing Gfap and an overall number ratio of Gfap/Pdgfra-positive cells greater than 10 to 1 within lesion cores (Supplementary Fig 11c–h) compared with a 2.5–1 ratio within healthy tissue (Fig. 3c). Graft-derived cells in lesions also expressed several additional molecular markers associated with host-derived astrocyte borders including Lcn2, Clu and Cpe (Supplementary Fig. 12a–d). Approximately 5% of HA-positive NPC grafted into stroke lesions resided within spared neural tissue adjacent to lesion cores (Supplementary Fig 11b, c). This sub-population of grafted cells differentiated into Pdgfra-positive OPC-like cells at greater proportions (33.5% of all cells in spared neural tissued) compared to the cells residing in the lesion core (8.4% of cells in lesion core) which equated to over 5-fold more Pdgfra-positive OPC-like cells compared with Gfap-positive astroglia in spared neural tissue (Supplementary Fig 11e, f).

Ischemic stroke lesions with NPC grafts lacked the clearly defined and sharp astroglia borders that were characteristic of untreated stroke and instead exhibited a contiguous bridge of Gfap-positive tissue that filled the entire lesion core (Fig. 4b–e). To examine this phenomenon further, we grafted NPC into transgenic mice expressing Gfap-TK in host astrocytes to compare the contributions of host-derived and graft-derived Gfap-positive cells. This comparison revealed that Gfap-positive cells throughout the lesion core were derived overwhelmingly from transplanted cells (Supplementary Fig 11g, h).

We next examined the effects of NPC grafts into SCI (Fig. 5a). As expected^1,61^, untreated crush SCI caused focal compartmentalized lesions similar to untreated L-NIO stroke, with a dense core of Cd13-postive non-neural cells consisting mainly of infiltrating macrophages and fibrotic scar surrounded by a border of newly proliferated astrocytes (Fig. 5b). SCI treated with DCH_MO_ hydrogel only also exhibited similar discretely compartmentalized lesions with Cd13-positive cores surrounded by host-derived border-forming astrocytes (Fig. 5b). NPC transplanted at two days after SCI and evaluated at 2 weeks survived robustly and differentiated into Gfap-positive cells that populated lesion cores and formed contiguous cell clusters and tracts throughout the lesions (Fig. 5b). NPC grafting also significantly reduced total Cd13-postive cells and increased Gfap-positive cells within SCI lesions (Fig. 5c, d), but remodeling of lesions with graft-derived cells was not as comprehensive after SCI as after stroke injury. In stroke, grafted NPC-derived cells essentially filled the entire lesion core and there were few isolated volumes of infiltrating inflammatory cells and fibrosis, whereas in SCI, there were discrete, and sometimes large, pockets of Cd13-positive immune cells surrounded by grafted cell borders (Fig. 5b, e). These differences could be due to differences in lesions generated by ischemic stroke injury with little or no bleeding or by crush SCI with clear evidence of bleeding and pronounced hemorrhagic necrosis^62^. Nevertheless, even after SCI, lesions with NPC grafts exhibited significantly reduced infiltration of peripheral inflammatory cells and reduced amounts of fibrotic tissue (Fig. 5c, d), and there were prominent Gfap-positive cell tracts that surrounded inflammatory and fibrotic cells throughout the lesion core in all animals, and these tracts provided bridges of contiguous Gfap-positive cells that spanned the SCI lesions across their rostral to caudal borders (Fig. 5b, e). Comparisons using separate molecular markers of host (TK) and graft (HA) derived Gfap-positive astroglia in the same animals demonstrated that the bridges spanning the lesion core were comprised exclusively of grafted cells and that the grafted cells integrated seamlessly with host astrocytes (Fig. 5e). Notably, although these NPC-derived astroglial bridges did not encourage significant spontaneous infiltration of neurofilament-positive axons, previous studies show that appropriately stimulated and chemoattracted host axons can grow along host astrocytes bridges in lesions^61,63^, suggesting that NPC-derived astroglial bridges warrant future testing for this capacity.

**Fig. 6:**
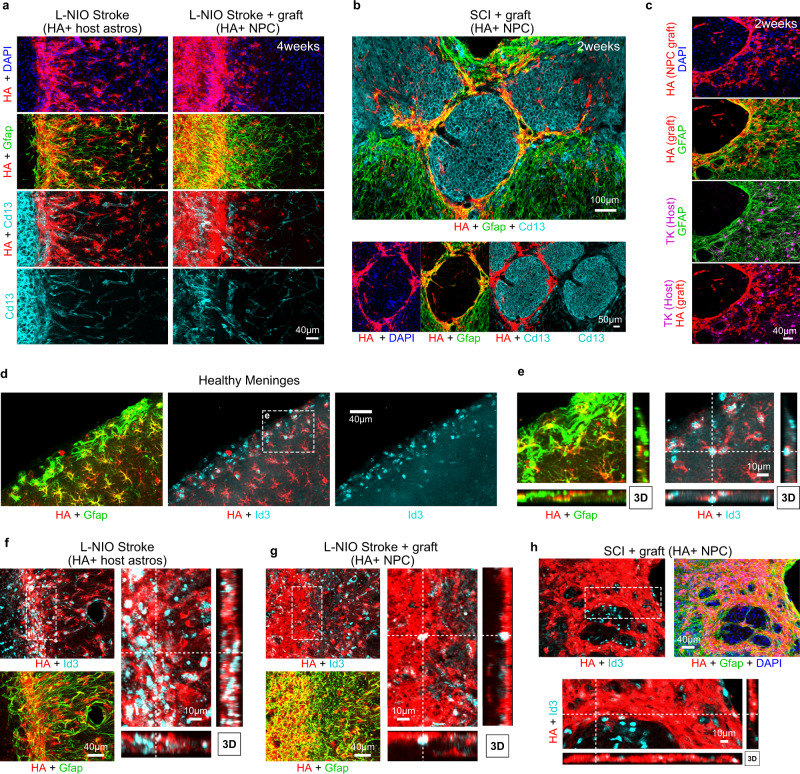
Astroglia derived from NPC grafted into CNS lesions exhibit features of border-forming astroglia. **a** Detailed IHC images showing the astrocyte border phenotype of stroke lesions at 4 weeks in untreated stroke only and NPC grafted strokes. **b** Survey IHC of NPC grafted SCI lesions at 2 weeks showing NPC-derived astrocyte borders formed around Cd13-positive inflammatory cells. **c** Detailed IHC images showing the astrocyte border phenotype in SCI derived from transplanted NPC and not from host astrocytes (TK-positive). **d**, **e** Survey and detailed, higher magnification 3D IHC imaging of healthy mouse cortex and meninges showing higher expression of transcriptional regulator Id3 (inhibitor of DNA binding 3) in host astrocytes (HA+ Gfap-Cre-RiboTag) that form glia limitans at the meninges and penetrating blood vessels. **f** High magnification 3D IHC images showing astrocyte borders formed at stroke lesions upregulate expression of Id3. **g** High magnification 3D IHC images showing grafted NPC that remodel stroke lesions also express Id3. **h** NPC grafted into SCI lesions that form astrocyte borders also express Id3.

NPC grafted into stroke and SCI organized into contiguous borders along the entire surface interface between viable neural tissue and the stromal and inflammatory cells of lesion cores after both stroke and SCI in a manner similar to host border-forming astrocytes (Fig. 6a–c). Host astrocytes that form borders around CNS injury lesions exhibit characteristics and functions similar to glia limitans astrocytes along all meningeal borders in the healthy CNS^5,64,65^. Glia limitans astrocytes that abut the fibroblast lineage cells of the meninges or penetrating blood vessels with meningeal borders selectively express the transcription factor Id3^66^ (Fig. 6d, e, Supplementary Fig. 12e, f). We found that this Id3 expression is shared not only by host border-forming astrocytes around lesions, but also by NPC graft-derived astroglia that similarly form such borders (Fig. 6f–h) further supporting similarities between these cell types. In contrast, Id3 expression is sparce among other astrocytes located distant to borders with either meninges, large vessels or injury lesions (Fig. 6d–h, Supplementary Fig. 12e, f).

These findings show that NPC grafted into forebrain stroke or thoracic SCI lesions at 2 days after injury survive well, persist and populate the lesion cores with Gfap-positive cells. Notably, graft-derived Gfap-positive cells spread throughout the lesion cores and significantly reduced the amount of tissue exhibiting inflammation and fibrosis after both stroke and SCI. In addition, grafted NPC-derived cells formed contiguous tracts of Gfap-positive cells that spanned across lesions and integrated seamlessly with host astrocytes, thereby forming astroglial bridges that interconnected preserved neural tissue on opposite sides of lesions after both stroke and SCI.

### NPC grafted into subacute SCI and host wound repair astrocytes exhibit converging transcriptional profiles

**Fig. 7:**
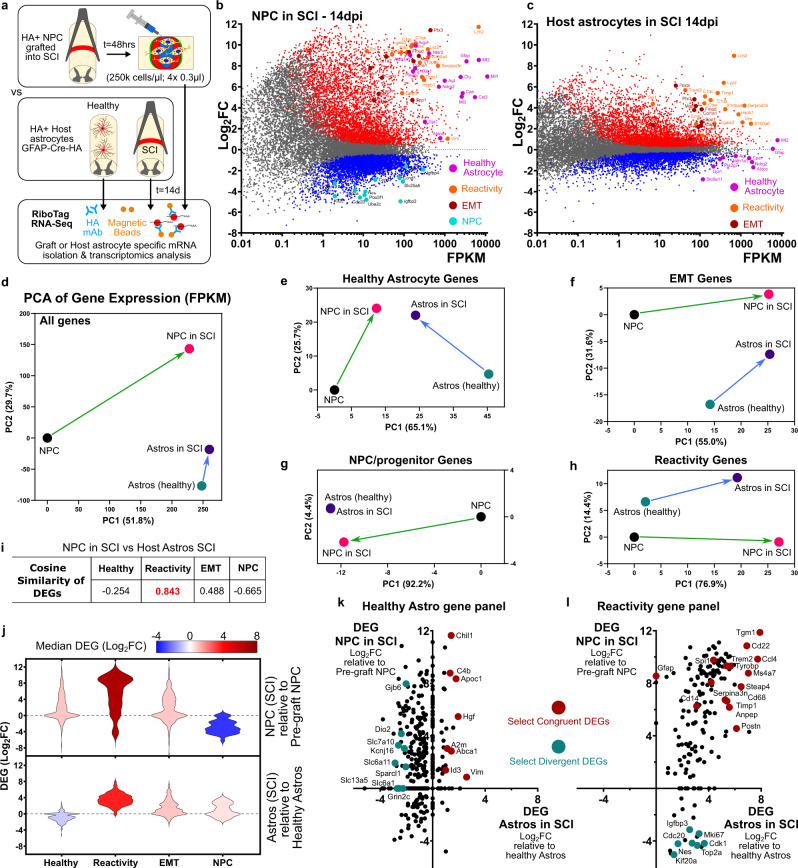
Astroglia derived from NPC grafted into SCI exhibit similar astroglial reactivity states to host astrocytes responding to SCI. **a** Schematic summarizing experimental approach for evaluating transcriptomes of NPC grafted into SCI with host astrocytes responding to SCI. **b** M-A Plot of grafted NPC (*N* = 5 samples) recovered at 2 weeks post injury (14dpi) compared to pre-grafted NPC (*N* = 5 samples) showing increased expression of healthy astrocyte, reactivity, and EMT genes and decreased expression of NPC/proliferation genes. **c** MA-Plot of host astrocytes at 14 days after SCI showing comparable upregulation of reactivity and EMT genes but decreased expression of healthy astrocyte genes. PCAs using FPKM values comparing state changes between NPC before and after grafting into SCI (recovered at 14dpi) and host astrocytes before and 14 days after SCI for all expressed genes (**d**) as well as using gene panels for healthy astrocytes (**e**), EMT (**f**), progenitors (**g**) and reactivity genes (**h**). **i** Cosine similarity of DEGs comparing state changes in NPC after grafting with that of host astrocytes after SCI. NPC and astrocytes show similar reactivity and EMT gene changes but divergent changes in astrocyte and NPC/proliferation genes. **j** Violin plots of DEGs for NPC grafted into SCI and host astrocytes in SCI for astrocyte, reactivity, EMT, and NPC/proliferation gene panels. **k**, **l** Plot of DEGs from the healthy astrocyte (**k**) and reactivity (**l**) gene panels for NPC after grafting compared with astrocytes after SCI showing strong congruent regulation) of reactivity genes and genes associated with glia limitans borders (maroon). Genes from the reactivity gene list but that are also associated with proliferation or progenitor phenotype as well as neural tissue support function show divergent regulation (cyan).

**Fig. 8:**
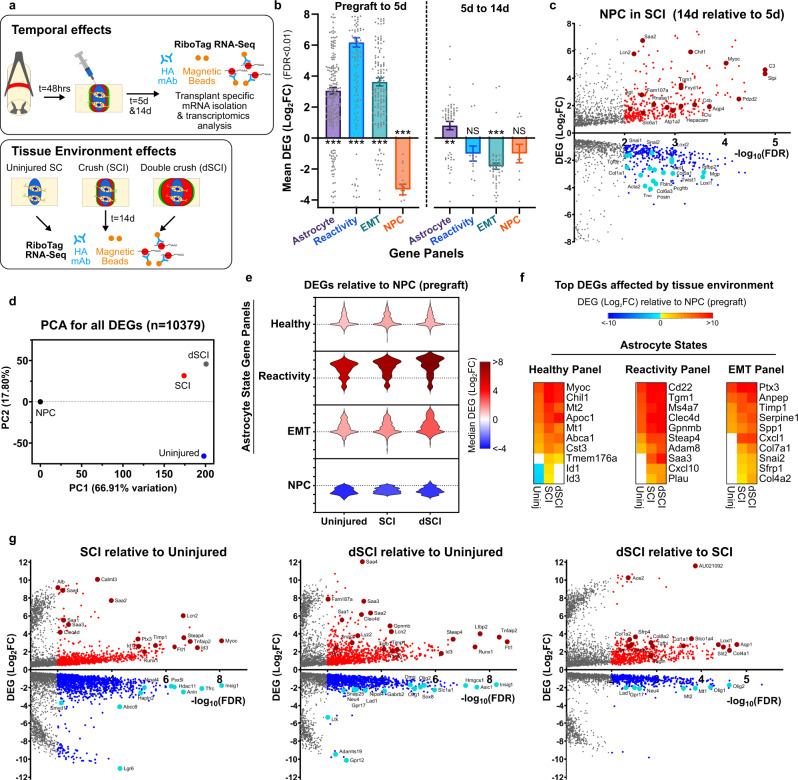
Astroglia derived from NPC grafted into subacute crush spinal cord injury mature over time and are altered non-cell autonomously by grafted environment. **a** Schematic summarizing experimental approach for evaluating temporal and tissue environment effects of NPC grafting into mouse SCI (*N* = 5 mice per condition). **b** Bar graph of DEGs for astrocyte, reactivity, EMT and NPC/progenitor gene panels for NPC in SCI recovered at 5 days relative to Pre-graft NPC and NPC recovered at 14 days relative to 5 days. Expression of all astrocyte state gene panels increased by 5 days whereas NPC gene expression decreased. From 5 to 14 days after grafting, astrocyte gene expression in NPC increased whereas EMT gene expression decreased. ***p value < 0.0005, **p value < 0.004, One sample *t*-test (two-tailed) for hypothetical mean = 0. Graphs are mean ± s.e.m. and individual DEGs are overlayed. **c** Volcano Plot comparing DEGs for NPC grafted into 2d SCI and recovered at 14 days after grafting versus samples recovered at 5 days after grafting. **d** PCA for all DEGS for NPC grafted into uninjured, single crush SCI (SCI) and double crush SCI (dSCI) referenced to NPC (pre-graft) showing large differences for all differentiation conditions compared to the NPC state and the variation amongst the unique tissue environments. **e** Violin plots of DEGs for NPC grafted into different tissue environments for astrocyte, reactivity, EMT and NPC/proliferation gene panels. **f** Heat maps showing the top 10 DEGs for the astrocyte, reactivity and EMT gene panels most altered in grafted NPC by tissue environment. **g** Volcano Plot comparin**g** DEGs for NPC grafted into different tissue environments.

We next dissected transcriptional characteristics of grafted NPC in vivo. For this, we first focused on transplantation of RiboTag-NPC after SCI in order to make direct comparisons with existing datasets on host astrocyte responses to SCI derived using the same RiboTag IP method^61,65^, and for ease of collecting high-quality tissue restricted to the lesion site from SCI versus forebrain stroke. The RiboTag procedure enabled recovery of mRNA selectively from small populations of transplanted NPC and their progeny with high yield and specificity (Fig. 7a, Supplementary Fig. 13a, b). NPC grafted at 2 days after SCI and harvested at 14 days showed net upregulation across all astrocyte state gene panels including from the healthy, reactivity and EMT panels with a concurrent downregulation of NPC genes when compared with pre-graft NPC profiles (Fig. 7b). Host border-forming astrocytes exhibited a comparable upregulation of reactivity and EMT genes at 14 days after SCI together with a net downregulation of genes associated with healthy astrocyte functions, consistent with their new primary interactions with non-neural lesion core cells rather than with neural cells (Fig. 7c). PCA and CS analyses of gene expression by FPKM values revealed that from very different transcriptional starting states, NPC and host astrocytes at SCI lesions both converged to remarkably similar astroglial reactivity states despite the large differences in their overall transcriptional state changes (all genes) from their original starting conditions as either proliferating NPC or non-proliferating astrocytes resident in healthy tissue (Fig. 7d–l). The gene panel for reactivity in particular showed remarkable congruency in the responses of host astrocytes and NPC with comparable vectors in Euclidean PCA space and a CS = 0.843 (Fig. 7h, i). Reactivity genes such as *Tgm1, Cd22, Serpina3n, Spi1*, and *Timp1* showed highly correlated congruent elevated expression in both NPC and host astrocytes in SCI (Supplementary Fig. 14, Fig. 7l), consistent with findings that many astrocyte reactivity genes after SCI are not detectably expressed in either healthy astrocytes^67^ or NPC. Other reactivity genes associated with injury-induced astrocyte proliferation (e.g. *Top2a, Nes, Mki67, Cdk1*) were divergently regulated because NPC downregulate these genes from high levels upon grafting, whereas host cells upregulated them from very low levels after injury (Supplementary Fig. 14, Fig. 7g, j, l). Transcriptome analysis of healthy astrocyte gene expression by grafted NPC and host astrocytes in SCI also revealed convergence of gene expression FPKM levels (Fig. 7g, Supplementary Figs 14 and 15) reached through divergent regulation of these genes by NPC and host astrocytes (Fig. 8j, k). such that host astrocytes showed significantly reduced expression of healthy astrocyte genes related to specialized neural tissue support functions such as synapse maintenance (*Sparc, Sparcl, Gpc5*), channels (*Kcnj10, Kcnj16, Gjb6*), and molecular transport (*Atp1a2, Dio2, Slc1a2, Slc6a1, Slc6a11, Slc7a10*) after SCI (Fig. 7k, Supplementary Fig. 15), whereas in grafted NPC, these neural tissue support function genes were instead upregulated from pre-graft levels and reached FPKM levels comparable to host astrocytes responding to SCI (Fig. 7k). Nevertheless, in NPC grafts the expression of neural tissue support genes failed to reach the high levels observed for host astrocytes in the uninjured state, likely due to the prioritization of border-forming functions and the limited interaction of grafts with host neurons at SCI lesions (Supplementary Fig. 15). Notably, both NPC and host astrocytes increased or sustained expression of astrocyte genes associated with glia limitans borders^66^ (e.g. *Id3, Vim, Clu, Chil1, C4b*) (Supplementary Fig. 14, Fig. 7k).

These findings show that NPC transplanted into CNS lesions and host astrocytes proliferating in response to CNS lesions exhibit converging transcriptional profiles during CNS wound repair which generates astroglial borders that surround and isolate CNS lesions.

### NPC grafted into SCI mature over time in vivo

Host astrocyte border formation around CNS lesions is a dynamic process that evolves over time during the 14 days after SCI and is likely to be influenced by molecular cues in the lesion environment^58^. To evaluate NPC graft transcriptome at an earlier timepoint during border formation we harvested grafts at 5 days after transplantation (Fig. 8a–c, Supplementary Fig. 13c). At 5 days post grafting, NPC show significant upregulation of all astrocyte state gene panel genes with reactivity genes showing the greatest net upregulation with a Log2FC ~ 6 compared to pre-graft levels (Fig. 8b). This astrocyte differentiation of NPC by 5 days occurred concurrently with downregulation of progenitor genes. From 5 to 14 days after transplantation, NPC grafts significantly downregulated EMT-related genes and upregulated healthy astrocyte genes while reactivity and NPC genes did not change significantly from their levels detected at 5 days (Fig. 8b, c, Supplementary Fig. 13c, d). These data suggest that astrocyte differentiation and cessation of proliferation occur quickly after transplantation, but maturation and adoption of specialized astrocyte functions proceeds more slowly. Key EMT regulatory genes more highly expressed in 5-day grafts included the canonical markers *Snai2, Postn*, *Fbln2* and *Acta2* (Fig. 8b, Supplementary Fig. 13d). The downregulation of EMT gene expression from 5 to 14 days coincides with consolidation of wound repair functions and stabilization of astroglial borders as well as reduced blood and serum exposure. Concurrently, healthy astrocyte gene expression increased from 5 to 14 days including genes required for forming and maintaining astrocyte border functions such as *Chil1* and *Myoc*^66,68–70^ as well as specialized astrocyte functional genes such as *Aqp4, Agt, Hepacam, Slc6a1* (Fig. 8b, c, Supplementary Fig. 13d).

These data show that differentiation of NPC grafted into acute lesion environments evolves over the temporal course of wound healing, and that reactivity and EMT-like transcriptional profiles are provoked acutely and then transition to profiles more similar to wound repair astrocytes over time.

### NPC transcriptional profiles and differentiation potential in vivo are modified by non-cell autonomous cues in different host tissue environments

Differentiation of grafted NPC into different phenotypes detected by IHC correlated with the spatial proximity of NPC to preserved neural tissue, lesion borders or pockets of immune and fibrotic cell-laden lesion cores. NPC progeny located within neural tissue expressed either Gfap or Pdgfra/Olig2 (Fig. 4d, e) whereas NPC at lesion borders overwhelmingly expressed Gfap, and NPC surrounded only by non-neural cells within SCI lesion cores expressed none of these markers, suggesting a strong environmental influence on NPC functions (Fig. 6b, c). We therefore next used RNA-Seq to further dissect the effect of molecular environment on non-cell autonomous regulation of NPC phenotype. We grafted NPC into three different conditions in the spinal cord that would direct unique predominant differentiation phenotypes that could be readily evaluated by bulk RiboTag transcriptomics: (i) uninjured spinal cord, (ii) single forceps crush SCI (SCI) as done previously, and (iii) double forceps crush SCI (dSCI) that created substantially larger non-neural cores and concurrent decreased influence of preserved neural tissue at the margins of the lesion on NPC (Fig. 8a, d–g, Supplementary Fig. 13e, f). NPC transcriptome analysis at 14 days revealed both common changes in NPC grafts that were not altered by tissue environment, and lesion environment-specific effects (Fig. 8e–g). Numerous healthy astrocyte genes were upregulated by at least Log_2_FC > 5 upon grafting versus pre-graft NPC gene expression regardless of tissue environment or lesion size including *Gfap, Atp1a2, Pla2g7, S100b, Gjb6, Aqp4* (Supplementary Table 4). Further analysis of NPC grafted into these different tissue environments revealed significant environment-dependent regulation of other healthy astrocyte genes including astrocyte glia limitans and border-forming genes^66,68–70^ *Id3, Myoc*, and *Chil1* which were most highly upregulated in SCI consistent with a higher proportion of grafted cells adopting astroglia border functions in response to injury (Fig. 8f, g, Supplementary Fig. 13e, f). Interestingly, expression of healthy astrocyte genes was highest in the SCI environment but decreased slightly for grafts in the larger double crush lesions (dSCI) (Supplementary Fig. 13e, f).

Consistent with our histological observations in the forebrain, NPC exhibited different proportions of immunohistochemically identified oligodendroglia and significantly different oligodendroglia transcriptional profiles when grafted into healthy tissue versus SCI lesions (Supplementary Fig. 16). NPC grafts in all tissue environments showed increased oligodendroglia lineage gene expression profiles (Supplementary Fig. 16a, b) but the overall expression levels of most of these genes including canonical OPC genes *Pdgfra, Gpr37, Hapln2 and Olig 2*, were reduced in grafts made in SCI lesions compared with uninjured tissue (Supplementary Fig. 16c–e). This reduction in the proportion of OPC-like graft progeny in SCI grafts measured by transcriptomics was corroborated with immunohistochemistry where NPC grafts in uninjured neural tissue showed widespread expression of OPC marker Olig2 and a uniform dispersion throughout tissue beds populated with neurons (Supplementary Fig. 16f, g). By contrast, NPC grafts made in SCI lesions showed Olig2 positivity only in a minority of cells found in regions where grafted cells made direct contact with preserved neural tissue whereas grafted cells in close proximity to, or within, SCI lesion cores showed negligible Olig2 expression (Supplementary Fig. 16h, i). Reactivity genes were the most differentially altered by tissue environment of the three unique astrocyte state gene panels (Fig. 8e, f, Supplementary Fig. 13f) with immune and inflammation response-related reactivity genes such as *Cd22, Tgm1, Ms4a7*, and *Clec4d*, most prominently varied by environment (Fig. 8g). NPC grafted into more severe dSCI environments with less exposure to neural tissue also exhibited increased expression of canonical EMT genes including *Anpep*, *Snai2 and Timp1* whereas less severe SCI lesions showed negligible increase in these genes compared to NPC grafts in uninjured tissue (Fig. 8f, g, Supplementary Fig. 13f).

These data show that the transcriptional profiles of NPC transplanted in vivo are significantly modulated by non-cell autonomous cues present in host tissue in a manner consistent with the generation of different types of progeny when grafted into different types of host tissue environments.

## Discussion

**Fig. 9:**
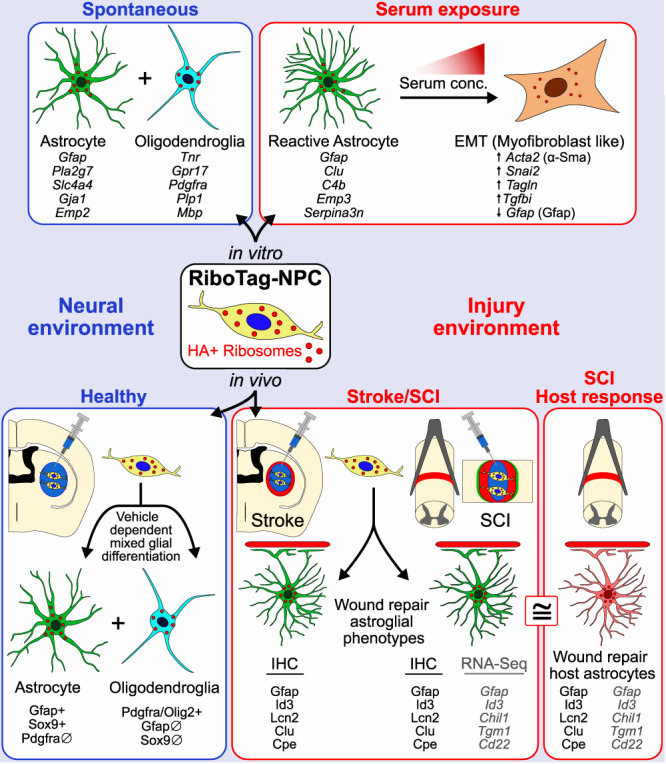
Schematic summarizing the main findings. RiboTag-NPC were derived by neural induction of mESC and were used for RNA-Seq and IHC studies in vitro and in vivo. When placed into in vitro (top left) or in vivo (bottom left) neural environments, RiboTag-NPC spontaneously differentiated into mixed populations of astrocyte lineage and oligodendrocyte lineage cells. Glial cell differentiation was verified based on gene expression determined by bulk RiboTag RNA-Seq and single-nuclei RNA-Seq. Introducing NPC into CNS injury-like environments in vitro (via exposure to increasing concentrations of serum (top right) or grafting in vivo into lesion environments after stroke and spinal cord injury (bottom right), instructed cells to adopt wound repair astroglial phenotypes through non-cell autonomous cues. The wound repair astroglial phenotype adopted by grafted NPC in CNS injuries shared numerous protein and transcriptome similarities (≅) with the host astrocyte wound repair response (bottom right). Example proteins and genes (italicized) shared by these wound repair phenotypes are listed. Notably, high serum concentrations in vitro (top right), or grafting into larger and more severe lesions (not depicted), increased myofibroblast-like phenotypes in NPC characteristic of EMT. EMT epithelial mesenchymal transition, HA haemagglutinin, IHC immunohistochemistry.

Directing the fate of transplanted NPC into desired cell phenotypes to serve specific therapeutic purposes after transplantation in vivo will require a detailed understanding of the relative roles of non-cell autonomous cues and cell-autonomous programs in determining NPC fate. Although NPC can be matured into neuronal or glial restricted progenitors in vitro that tend to give rise preferentially to neurons or glia after grafting in vivo^33^, the pronounced degree to which different host cell environmental cues modulate NPC differentiation is only beginning to be recognized, specified and addressed^71^. Our findings here demonstrate the power of the Rpl22-HA (RiboTag) tool for transcriptionally profiling and morphologically characterizing NPC and their progeny after grafting under different conditions in vivo. Using this approach, we compared the transcriptional states of NPC grafted into uninjured or lesioned CNS tissue with the transcriptional changes induced in NPC by exposure to specific molecular cues in vitro and with transcriptional profiles of endogenous CNS wound repair cells. Our findings show that (i) NPC fate is strongly influenced by different non-cell autonomous cues in vitro and in different host CNS environments in vivo, and (ii) NPC grafted into CNS subacute lesions after stroke or SCI adopt a phenotype similar to newly proliferated host astroglial that participate in wound repair (Fig. 9).

After mitogen withdrawal in vitro, RiboTag-NPC spontaneously differentiated primarily into astrocyte and oligodendrocyte lineage cells as reported for NPC by others^36^. This cell-autonomous differentiation in vitro was modified as expected by known non-cell autonomous cues such that exposure to CNTF or 1% serum generated more astroglia^36,39,40^, whereas IGF-1 generated more oligodendroglia^41^. NPC grafted into healthy CNS tissue generated cells with transcriptional phenotypes similar to astroglia and oligodendroglia present in healthy adult CNS tissue or to cells generated after NPC differentiation in vitro. Notably, NPC grafted into uninjured CNS tissue intermingled with local neurons and upregulated genes associated with neuronal interactions. In striking contrast, these NPC when grafted into subacute ischemic or hemorrhagic CNS lesions and exposed to serum or blood from the blood–brain barrier leak, differentiated primarily into cells with transcriptional and morphological features similar to newly proliferated host astrocytes that surround lesions and corral inflammatory and fibrotic cells. Notably in such lesions, NPC progeny did not intermingle with nearby neurons and did not upregulate genes associated with neuronal interactions, but instead interacted with inflammatory and fibrotic tissue and upregulated genes associated with wound repair. To look for potential non-cell autonomous cues underlying these differences in grafted NPC fate, we examined NPC responses to serum proteins in vitro. Serum response factor (Srf) is a prominent transcription factor of astrocyte reactivity across multiple disorders, including SCI and stroke^67^. We found that serum proteins at lower concentrations fostered NPC acquisition of astroglial features but at higher concentrations attenuated astroglia features and promoted EMT-like changes. In vivo, we noted changes compatible with these in vitro findings such that NPC located in the center of large lesions and thereby exposed to high serum and inflammatory/fibrotic cells but not to neural cells exhibited more EMT-like features, whereas NPC located along borders between inflammatory/fibrotic cells and neural cells and thereby exposed to mixed cues from both cell populations were directed towards a border-forming astroglial phenotype, indicating that different microenvironments within lesions can differentially influence NPC cell fate. Thus, non-cell autonomous cues derived from various sources, including blood-borne molecules, leukocytes or local neural cells present in different host tissue environments can powerfully modulate grafted NPC transcription and instruct differentiation into cells with different phenotypes. This differential responsiveness of NPC to different non-cell autonomous cues after grafting in vivo strongly supports the notion that understanding these cues will foster the development of bioengineered interventions that can intentionally provide desired, or block unwanted, cues and thereby direct grafted NPC into therapeutically useful phenotypes^71^.

During naturally occurring wound repair in the adult CNS, neural tissue that has been lost to injury or disease is replaced by non-neural fibrotic tissue that is partitioned away from adjacent viable neural tissue by protective astroglial borders^1–10^. This stereotypic organization of CNS lesions is conserved across rodents and humans, and the newly proliferated astroglia that form narrow borders between neural and non-neural tissue during wound repair share similarities with astroglial that form narrow ‘limitans’ borders separating neural from non-neural tissue along meninges in the healthy CNS^5^.

Here, we found that NPC grafted into subacute CNS lesions differentiated into an astroglial phenotype with striking similarities to that adopted by proliferating host astrocytes that generate naturally occurring astroglial borders around non-neural lesion core tissue. Starting from two very different transcriptional states, transplanted NPC and proliferating host astroglia in serum-exposed lesions exhibited convergent differentiation into astroglia with similar transcriptional, morphological, and functional features. In this regard, it deserves mention that the peak time of host-derived astrocyte proliferation for border formation is from 2 to 7 days after injury^58^ so that NPC grafted at 2 days after stroke or SCI, as done here, were placed into an environmental niche potentially primed to direct them into such border-forming cells. It is interesting to speculate that NPC and proliferating astrocytes share similar differentiation potentials and that the same molecular cues in lesions might have similar effects on their differentiation. Other researchers have shown that adult astroglia induced to proliferate after injury and then placed into neurogenic culture conditions exhibit differentiation potentials similar to NPC^64,72^, providing further evidence for similarities between these cells. Likely sources of differentiation modifying cues are serum-derived and blood-borne molecules that are present in high concentrations in ischemic or hemorrhagic lesions during the timepoint of astroglial proliferation and border formation. We show here that serum exposure in vitro alters the transcription of NPC differentiating in vitro to become similar to that of reactive astrocytes in CNS lesions. In addition, we show that border-forming astroglia derived from either grafted NPC or host proliferating astrocytes, as well as serum-exposed NPC in vitro, all upregulate the transcription regulator Id3, which is also expressed by astroglial limitans borders along meninges and large vessels in healthy tissue but is rare among other astrocytes. Interestingly, other researchers have shown that blood-derived fibrinogen can instruct endogenous periventricular NPC to upregulate Id3 and adopt a wound repair astroglial phenotype^73^. Together, these findings support a model whereby newly proliferated astroglia that form borders around CNS lesions represent a naturally occurring wound repair phenotype^1,4,5,10,74^ and that non-cell autonomous cues in serum-exposed CNS lesions activate similar intrinsic potentials in grafted NPC and proliferating host astroglia towards this naturally occurring wound repair astroglia phenotype.

NPC derived from ESC or iPSC and grafted into CNS lesions are well known to bias towards astroglial-like differentiation with formation of few neurons in the absence of exogenous interventions, but why this occurs and the degree to which it is cell autonomously or non-cell autonomously determined have not been well understood^71^. Because NPC generate primarily astroglia instead of neurons when transplanted into CNS lesions, the lesion environment is often regarded as hostile. Our findings challenge the notion that lesions are ‘hostile’ to grafted NPC and instead provide an explanation that NPC are responding to cues in the lesion environments that instruct NPC and host proliferating astrocytes to adopt a naturally occurring wound repair astroglial phenotype. Understanding how and why different non-cell autonomous cues instruct NPC transplanted in CNS host tissue to generate different cell types will be fundamental to achieving mechanism-based approaches to therapeutic NPC transplantation. Monitoring transcriptional profiles of transplanted NPC in vivo over time as described here can facilitate the dissection of such mechanisms.

## Methods

### Cells

Mouse embryonic stem cells (mESC) were derived from the inner cell mass of E3.5 blastocyst stage embryos generated from crosses of male homozygous B6N.129-Rpl22tm1.1 Psam/J(RRID: IMSR_JAX: Stock No: 011029) “Ribotag” mice to females hemizygous for a dominant, maternal effect cre allele, B6.Cg-Tg(SOX2-cre)1Amc/J (RRID: IMSR_JAX: 008454) and heterozygous for the “RiboTag” allele. Multiple male and female mESC lines were derived, and each was karyotyped and genotyped to confirm sex and homozygosity for the cre-exised, Ribotag allele^35^. A single female mESC line was used for all experiments in this work. These RiboTag mESC express a modified ribosomal protein L22 (Rpl22) with hemagglutinin HA epitope tag^34^.

### Animals

All in vivo experiments involving the use of mice were conducted under the continual ethical oversight of the Animal Research Committee (ARC) of the Office for Protection of Research Subjects at the University of California Los Angeles (UCLA) as detailed in the approval protocols ARC-2000-001, ARC-2008-051, ARC-2015-073. All in vivo animal experiments were conducted within approved UCLA facilities using wildtype or transgenic C57/BL6 female and male mice that were aged between 8 weeks and four months old at the time of craniotomy or spinal cord injury surgery. B6N.129-Rpl22tm1.1Psam/J (RRID: IMSR_JAX: 011029) were bred with B6.Cg-Tg(Gfap-cre)73.12 Mvs/J (RRID: IMSR_JAX: 012886) from an in-house colony to generate Transgenic 73.12 GFAP-Cre -RiboTag mice. B6.Cg-Tg(Gfap-TK)7.1Mvs/J (RRID: IMSR_JAX: 005698) were bred from an in-house colony to generate GFAP-TK mice. Mice were housed in a 12-h light/dark cycle in a specific pathogen-free facility with controlled temperature (20–25 °C) and humidity (50–70%) and were provided with food and water ad libitum. Mice that received surgical procedures were administered post-surgical analgesia (buprenorphine, 0.1 mg/kg) for at least 2 days after each surgery. Spinal cord injury mice received twice daily bladder expression until voluntary voiding returned. No animals in the study that received NPC transplants were administered with any immunosuppression drugs.

### NPC derivation from mESC

Neural progenitor cells (NPC) were derived from mouse embryonic stem cells (mESC) by adapting and refining existing protocols used by us and others^35,36,75^. mESCs were maintained and expanded on 0.1% gelatin-coated flasks (EmbryoMax™ 0.1% Gelatin Solution, Millipore Sigma, Cat# ES006B) in KnockOut™ DMEM media (Cat# 10829018, ThermoFisher Scientific) containing 15% Fetal bovine serum (FBS) (ES cell tested) (ThermoFisher Sci cat # 10439-016), MEM Non-Essential Amino Acids Solution (100X) (Cat# 11140050, ThermoFisher Scientific), 100X EmbryoMax® Nucelosides (EMD Millipore cat # ES-008-D), Beta-Mercaptoethanol (Sigma Aldrich M3148), Antibiotic-Antimycotic (100X) (Cat# 15240096, ThermoFisher Scientific) and Leukemia inhibitory factor (LIF) (1 million units/mL stock) (EMD Millipore cat # ESG1106). NPC lines were derived from mESCs using a standard 2−/4+ induction protocol followed by a neural expansion protocol^35,36,75^. The 2− step of embryoid body (EB) formation involved removing LIF and FBS supplements from the cell media and changing media to a differentiation media consisting of Advanced DMEM/F12 (ThermoFisher Sci cat # 12634-010) supplemented with L-Glutamine (Thermofisher Scientific Cat#25-030-081) (2 mM) and Knockout serum replacement (ThermoFisher Sci cat # 10828010) plus the other constituents described above for 2 days. Neural induction (4+) of embryoid bodies (EB) was performed by culturing isolated EB in differentiation media supplemented with retinoic acid (RA) (R2625-50MG, Sigma) (50 nM) and purmorphamine (PUR) (sonic hedgehog (SHH) agonist) (SML0868-5MG, Sigma) (500 nM) for 4 days. To propagate the neurally induced mESCs, the cells were grown in neural expansion media consisting of Advanced DMEM/F12 supplemented with B27 (no Vitamin A) (50X) (ThermoFisher Sci cat # 12587010) and growth factors EGF (Cat# AF-100-15-100UG, Peprotech) and FGF (Cat# 100-18B-100UG, Peprotech) (100 ng/mL for each) for a period of 2-6 weeks resulting in a passagable line of adherent NPC. Samples at defined passages after neural induction (P) were collected for analysis. NPC stocks were maintained so that all samples in the study were less than P30.

### In vitro NPC assays

NPC were differentiated spontaneously (Spont) by lowering the EGF/FGF concentration in the neural expansion media from 100 ng/mL to 1 ng/mL for 4 days. CNTF (Cat# 450-13-100UG, Peprotech) and IGF-1 (Cat#100-11-100UG, Peprotech) differentiation involved adding 100 ng/mL of the respective growth factor into the Spont media and culturing for 4 days. FBS differentiation involved adding FBS (Cat# 10437010, ThermoFisher Scientific) at 1, 5, 10, or 20% vol/vol to the Spont media and culturing for 4 days. For ICC evaluations cells were seeded onto 10 mm round glass coverslips that had been coated with 0.1% gelatin and placed into wells of a 24-well plate at 30,000 cells per well and cultured with 1 mL of media. At 4 days, cells on coverslips were fixed with 4% paraformaldehyde for 30 min. For RNA-Seq and Western blotting samples, cells were seeded at 30,000 cells/cm^2^ on T75 flasks (total of 2.25 million cells/flask) and recovered by gentle trypsinization and centrifugation before being frozen as cell pellets and stored in −80C cold storage until processing. TGF-βR inhibition of NPC cultured in 10% FBS was performed by preincubating NPC in media supplemented with the small molecule SB-431542 hydrate (S4317-5MG, Sigma) at a concentration 1 µM for 2 h prior to adding the FBS and then culturing for 4 days. Fractionation of FBS was performed using Amicon Ultra-0.5 Centrifugal Filter Unit (100 kDa MWCO, UFC510024, Sigma) spinning on a fixed angle rotor at 14,000 × *g* for 30 min to concentrate the >100 kDa fraction. Fractionation was performed in 12 separate filter units and the >100 kDa and <100 kDa fractions from each were pooled. The concentration factor for the >100 kDa sample was estimated from the pooled sample and diluted to equivalent concentration of the pre-fractionated FBS for use in the in vitro cell cultures. The <100 kDa fraction was used in the in vitro experiments as collected without any further dilution. Mouse serum was generated from pooled blood collected fresh from 12–16 week C57/BL6 female and male mice via cardiac puncture using a 3 mL syringe with a 18 gauge needle. Collected blood was transferred to a microcentrifuge tube and allowed to clot for 30 min at room temperature. Serum was isolated from the clotted blood by gentle centrifugation at 100 × *g* for 10 min in a refrigerated centrifuge (Model 5415R, Eppendorf).

### Synthesis and formulation of hydrogels

Physically crosslinked hydrogels were formulated from polypeptides that were synthesized using procedures developed previously by our group^1,35^. Polypeptide synthesis was performed in a N_2_ filled glove box using anhydrous solvents. Amino acid N-carboxyanhydride (NCA) monomers l-methionine (M) NCA, L-leucine (L) NCA and l-alanine (A) NCA were prepared by phosgenation in a tetrahydrofuran (THF) solution under inert conditions. NCA monomers were purified by either recrystallization (for L and A NCA) or column chromatography in the glove box (M NCA). Copolypeptides were prepared at 100 mg scale, by adding a solution of initiator, Co(PMe_3_)_4_, (3.5 mg, 0.001 mmol) in THF (20 mg/mL) to a mixture of l-methionine NCA (Met NCA; 100 mg, 0.57 mmol) and L-alanine NCA (Ala NCA; 7.3 mg, 0.063 mmol) in THF (50 mg/mL). After 2 h, l-leucine NCA (Leu NCA; 14 mg, 0.088 mmol) in THF (50 mg/mL) was then added and after a further 1 h the reaction mixture was removed from the glove box. The block copolypeptide solution was precipitated in deionized water, filtered, and dried under reduced pressure. Next, a volume of 70 wt. % *tert*-butyl hydroperoxide (TBHP) (16 molar equivalents per l-methionine residue) was added to the copolypeptide in DI water to convert l-methionine residues to l-methionine sulfoxide residues over 24 h at ambient temperature. To aid oxidation, a catalytic amount of camphorsulfonic acid (CSA) (0.2 molar equivalents per Met residue) was added to the solution. Polypeptide was dialyzed in 2000 MWCO dialysis bags against: (i) pyrogen-free deionized milli-Q water (3.5 L) containing sodium thiosulfate (1.2 g, 2.2 mM) for 1 day to neutralize residual peroxide, (ii) pyrogen-free deionized milli-Q water (3.5 L) containing ethylenediaminetetraacetic acid tetrasodium salt hydrate (1.0 g, 2.63 mmol) to aid cobalt ion removal, and (iii) pyrogen-free deionized milli-Q water (3.5 L) for 2 days to remove residual ions. For each step dialysate was changed every 12 h. The copolypeptide solution was then freeze dried to yield a white fluffy solid. Hydrogels were prepared by solubilizing lyophilized copolypeptide in phosphate buffered saline (PBS) or supplemented cell culture media and stored at 4 °C for 24 h to assemble, without stirring, before use. DCH formulations mixed with cells were prepared at either 10 or 5 wt% and then diluted 1:1 with cells in media via gentle mixing with a pipette. DCH alone formulations were prepared at 5% or 2.5% in PBS.

### Surgical procedures

All surgical procedures were approved by the UCLA ARC and conducted within a designated surgical facility at UCLA. All procedures were performed on mice under general anesthesia achieved through inhalation of isoflurane in oxygen-enriched air.

### Craniotomy procedure

Shaved mice heads were stabilized and horizontally leveled in a rodent stereotaxic apparatus using ear bars (David Kopf, Tujunga, CA). A small craniotomy over the left coronal suture was performed using a high-speed surgical drill and visually aided by an operating microscope (Zeiss, Oberkochen, Germany). As small rectangular flap of bone encompassing sections of the frontal and parietal bone was removed to expose the brain in preparation for injection.

### Stroke lesion model

To create focal ischemic strokes, 1.5 μL of L-NIO (N5-(1-Iminoethyl)-L-ornithine) (Cat. No. 0546, Tocris solution) (27.4 mg/mL (130 µM) in sterile PBS) was injected into the caudate putamen nucleus at 0.15 μL/min using target coordinates relative to Bregma: +0.5 mm A/P, +2.5 mm L/M, and −3.0 mm D/V. A standard micropipette injection protocol was used to make all injections into the brain or spinal cord using pulled borosilicate glass micropipettes (WPI, Sarasota, FL, #1B100-4) that were ground to a 35° beveled tip with 150–250 μm inner diameter. Glass micropipettes were mounted to the stereotaxic frame via specialized connectors and attached, via high-pressure polyetheretherketone (PEEK) tubing, to a 10 μL syringe (Hamilton, Reno, NV, #801 RN) controlled by an automated syringe pump (Pump 11 Elite, Harvard Apparatus, Holliston, MA). After injection, the surgical incision site was sutured closed and the animals were allowed to recover for 48 h before undergoing a second surgery involving injection of hydrogel with and without cells as described below.

### Spinal cord Injury crush model

A single incision was made along the back of mice and back musculature attached to the vertebrae cut and removed using small spring scissors and sterile cotton swabs. A laminectomy was made at the 10th thoracic (T10) spinal vertebrae level using spring scissors and 2SP laminectomy forceps (FST) to expose the dorsal surface of the spinal cord. A timed (5 s) lateral compression crush injury at the laminectomy site was made using No. 5 Dumont forceps beveled to a tip width of 0.5 mm.

### NPC and hydrogel injections

Cells and hydrogel formulations were backloaded into the pulled glass micropipettes prior to connecting to the stereotaxic frame. For brain injections, a volume of 1 μL of hydrogel and/or cells (loaded at 10,000, 100,000, or 250,000 cells/µL) were injected into the caudate putamen nucleus at 0.15 μL/min using target coordinates relative to Bregma: +0.5 mm A/P, +2.5 mm L/M. The pipette was lowered to −3.5 mm D/V for the start of the injection and moved up +0.5 mm twice over the course of the injection to a final location of −2.5 mm D/V to improve deposition of cells in brain tissue. The micropipette was allowed to dwell in the brain at the injection site for an additional 4 min at the end of the injection. The micropipette was then removed from the brain slowly and incrementally over a 2-min period. The same procedures were used for injections into L-NIO stroke lesions at 2 days after stroke induction. For hydrogel and/or cell injections into SCI lesions, at 2 days after SCI, NPC formulations were transplanted at a concentration of 250,000 cells/µL with four individual injections of 0.3 µL (4 × 0.3 µL = 1.2 µL total) made at the lesion site at a depth of 0.6 mm from the dorsal surface of the spinal cord.

### Transcardial perfusions

After terminal anesthesia by intraperitoneal injection of pentobarbitol, mice were perfused transcardially with heparinized saline (10 U/mL of heparin) and 4% paraformaldehyde (PFA) that was prepared from 32% PFA Aqueous Solution (Cat# 15714, EMS), using a peristaltic pump at a rate of 7 mL/min. Approximately, 10 mL of heparinized saline and 50 mL of 4% PFA was used per animal. Brains and spinal cords were immediately dissected after perfusion and post-fixed in 4% PFA for 6–8 h. After PFA post-fixing, brains and spinal cords were cryoprotected in 30% sucrose in tris-buffered saline (TBS) for at least 3 days with the sucrose solution replaced once after 2 days and stored at 4 °C until further use. For RiboTag RNA-Seq experiments mice were perfused transcardially with cold heparinized saline (10 U/mL of heparin) for 2 min (7 mL/min) and a 4 mm segment of spinal cord centered on the lesion was immediately dissected after perfusion on ice and snap frozen in microcentrifuge tubes on dry ice. Frozen spinal cords were kept at −80 °C until RiboTag Immunoprecipitation (IP) processing.

### Immunohistochemistry and immunocytochemistry

Coronal brain sections (40 µm thick) or horizontal spinal cord sections (30 µm thick) were cut using a Leica CM3050 cryostat. Tissue sections were stored transiently in TBS buffer at 4 °C or in antifreeze solution of 50% glycerol/30% Sucrose in PBS at −20 °C for long-term storage. Tissue sections were processed for immunofluorescence using free-floating staining protocols described in comprehensive detail previously using donkey serum to block and triton X-100 to permeabilize tissue^1,61,65^. The primary antibodies used in this study were: rabbit Hemagglutinin (HA) (1:1000, Sigma #H6908); goat HA (1:800, Novus, NB600-362); rabbit alpha-smooth muscle actin (α-Sma) (1:200, Novus, NB600-531); rabbit anti-Gfap (1:1000, Dako/Agilent, GA524); rat anti-Gfap (1:1000, Thermofisher, #13-0300); rabbit anti-NeuN (1:1000, Abcam, ab177487); guinea pig anti-NeuN (1:1000, Synaptic Systems, 266 004); goat anti-Cd13 (1:200, R&D systems, AF2335); rat anti-Galectin-3 (1:200, Invitrogen, 14-5301-82); rabbit anti-Fibronectin (1:500, Millipore, Cat#AB2033); rat anti-Cd68 (1:1000, AbDserotec-BioRad, MCA1957); rabbit anti-Iba-1 (1:800, Wako, 019-19741); guinea pig anti-Iba-1 (1:800, Synaptic systems, 234 004); rabbit anti-P2ry12 (1:500, Anaspec, AS-55043A); goat anti-Pdgfr-α (1:500, R&D systems, AF1062); goat anti-Nestin (1:500, R&D, AF2736); goat anti-Oct4 (1:500, R&D systems, AF1759); goat anti-Sox9 (1:500, R&D systems, AF3075); rabbit Aldh1l1 (1:1000, Abcam, Ab87117); rabbit anti-Amyloid Beta (Aβ) (1:200, Abcam, Ab2539); rabbit anti-Amyloid precursor protein (App) (1:200, abcam, ab32136); goat anti-Carboxypeptidase E/CPE (Cpe) (1:200, R&D systems, AF3587); goat anti-Lipocalin-2 (Lcn2) (1:200, R&D systems, AF1857), goat anti-Clusterin (Clu) (1:200, R&D systems, AF2747); Rabbit anti-Tuj-1 (1:500, Sigma, T2200-200UL); rat anti-Vimentin (1:500; R&D Systems, MAB2105); rat anti-Cd44 (IM7) (1:200; Thermofisher Scientific, #14-0441-82); goat anti-Dppa4 (1:200; R&D Systems, AF3730), Rabbit anti-Id3 (1:200; Cell Signaling Technology, #9837); rabbit anti–HSV-TK (1:1000) (generated by M. Sofroniew and validated previously^76^). All secondary antibodies used in this study were purchased from Jackson ImmunoResearch (West Grove, PA). All secondary antibodies were affinity purified whole IgG(H + L) antibodies with donkey host and target species dictated by the specific primary antibody used. All secondary antibodies were stored in 50% glycerol solution and diluted at a concentration of 1:250 in 5% normal donkey serum in TBS when incubated with brain histological sections. Nuclei were stained with 4′,6′-diamidino-2-phenylindole dihydrochloride (DAPI; 2 ng/mL; Molecular Probes). Acti-stain 555 phalloidin (Cytoskeleton Inc. Cat. # PHDH1-A) staining was performed in conjunction with DAPI nuclei staining using manufacturer’s instructions. Sections were coverslipped using ProLong Gold anti-fade reagent (InVitrogen, Grand Island, NY). Sections were examined and photographed using epifluorescence microscopy, deconvolution widefield fluorescence microscopy and scanning confocal laser microscopy (Zeiss, Oberkochen, Germany). Tiled scans of individual whole sections were prepared using a ×20 objective and the scanning function of a Leica Aperio Versa 200 Microscope (Leica, Wetzlar, Germany) available in the UCLA Translational Pathology Core Laboratory.

### RiboTag immunoprecipitation, RNA purification and RNA sequencing

Frozen 4 mm segments of SCI tissue containing HA-positive NPC transplants or pellets of cells collected from in vitro experiments were processed by RiboTag Immunoprecipitation (IP) using established methods^34,65^. SCI tissue or cell pellet samples were homogenized in supplemented homogenization buffer (50 mM Tris pH 7.4, 100 mM KCl, 12 mM MgCl_2_, 1% NP-40, 1 mM Dithiothreitol (DTT), 1× Protease inhibitors, 200 U/mL RNAsin, 100 mg/mL Cyclohexamide, 1 mg/mL Heparin) using 2 mL glass Dounce tissue grinders (D8938-1SET, Sigma). Tissue homogenates were centrifuged to remove tissue debris before being incubated with Anti-HA.11 Epitope Tag Antibody (Biolegend, Cat# 901515) for 4 h in a microcentrifuge tube on a microtube rotator kept at 4 °C. At the end of 4 h, IP solutions were combined with Pierce A/G Magnetic Beads (Thermofisher, #PI88803) and incubated overnight on a microtube rotator at 4 °C. On the second day, magnetic beads were washed three times with high salt solution (50 mM Tris pH 7.4, 300 mM KCl, 12 mM MgCl_2_, 1% NP-40, 1 mM Dithiothreitol (DTT), 100 mg/mL cyclohexamide). Unpurified RNA was collected from the magnetic beads by addition of RLT Plus buffer with BME and vigorous vortexing. RNA was then purified using RNeasy Plus Mini (for in vitro cell pellets) or Micro Kits (for spinal cord tissue) (QIAGEN Cat# 74134 and 74034). Total mRNA derived from the RiboTag IP was quantified using an automated electrophoresis bioanalyzer. RNA samples with RNA integrity numbers (RIN) greater than seven were processed for RNA Sequencing. Sequencing was performed on poly-A selected libraries using Illumina NovaSeq S2 (housed in the UCLA Technology Center for Genomics & Bioinformatics) using pair end reads (2 × 50 − 50 bp length) with an average of 50–100 M reads per sample. We performed sequence alignment and transcript counting on raw FASTQ files using standardized Galaxy workflows described in detail below.

### Nuclei preparation from in vitro cell cultures and single-nuclei RNA sequencing

Cultures of NPC and NPC differentiated using the Spont or CNTF conditions (~2.25 × 10^6^ cells per condition) were trypsinized and pelleted by centrifugation. Nuclei were extracted from cell pellets by gentle resuspension in ice-cold lysis buffer (10 mM Tris buffer, 10 mM NaCl, 3 mM MgCl_2_, 0.1% Nonidet P40 Substitute). Nuclei were pelleted by centrifugation (500RPM (Model 5415 R, Eppendorf) for 5 min at 4 °C) and then resuspended in Nuclei Wash and Resuspension Buffer (NWRB) (1× PBS, 1% BSA, 0.2 U/µL RNAse inhibitor). Nuclei were assessed by trypan blue staining before being washed once more in NWRB and then filtered using a 5 mL Polystyrene Round-Bottom Tube with 35 µm Cell-Strainer Cap and concentrated to a nuclei concentration of 1000 nuclei/µL (1 × 10^6^ nuclei/mL). Dissociated single-nuclei from the three conditions were processed using Chromium Next GEM Single Cell 3′ v3.1 kits using manufacturer’s instructions and libraries processed for RNA Sequencing using Illumina NovaSeq S2 (housed in the UCLA Technology Center for Genomics & Bioinformatics) using pair-end reads (2 × 50 − 50 bp length). Raw data files were processed using Scanpy workflows conducted using Galaxy and Galaxy Single Cell Omics platforms described in detail in the quantification section below.

### Western blotting

Protein was extracted from frozen cell pellets by homogenization in RIPA lysis buffer supplemented with PMSF, protease inhibitor, and sodium orthovanadate using a handheld grinder and attached pestle. Homogenized samples were kept on ice for 30 min after homogenization and then centrifuged to remove insoluble debris. Supernatants containing protein were decanted into fresh microcentrifuge tubes and stored at −80 °C until use. Total protein in solutions was quantified prior to gel loading using standard BCA assay (Pierce™, Cat#23225, Thermofisher) following manufacturer’s instructions. SDS-PAGE was performed using NuPAGE Novex Bis-Tris Pre-Cast Gels in XCell SureLock Mini-Cell Electrophoresis System. Protein samples were prepared in NuPAGE LDS Sample Buffer and heated at 70 C for 10 min prior to gel loading. For all runs, 15–30 μg protein per sample was used. Protein gel electrophoresis was run using 1× NuPAGE SDS Running Buffer and conducted at 150 volts for 1 h. Separated proteins in the gel were transferred to PVDF membranes using 1× NuPAGE Transfer Buffer and XCell Blot Module by applying 30 V for 1 h following the manufacturer’s instructions. Total protein across sample lanes transferred to the PVDF membrane was quantified using SYPRO Ruby. For western blotting, membranes were activated with methanol and blocked using non-fat milk in Tris Buffer Saline with Tween20 (TBST) for 1 h followed by primary antibody incubation overnight at 4 °C. Primary antibodies were prepared in 5%BSA/TBST. Primary antibodies used for WB included: rabbit anti-Fabp7 (1:2000, PA5-31864 Thermofisher); goat anti-Nanog (1:1000, AF2729-SP R&D Systems); rabbit anti-Oct4 (1:1000, MA5-14845, Invitrogen); goat anti-Sox9 (1:1000, R&D systems, AF3075); goat anti-Dppa4 (1:2000, Novus, AF3730); goat anti-Nestin (1:2000, R&D Systems, AF2736); rabbit alpha-smooth muscle actin (α-Sma) (1:2000), Novus, NB600-531; mouse anti-Gfap (1:3000, Sigma, Cat#3893). After primary incubation membranes were washed 3 times with TBST on a shaker before incubation with species-appropriate HRP-conjugated secondary antibody in 5% non-fat milk/TBST for 1 h. Secondary antibodies used were goat anti-rabbit (1:10,000, A27036, ThermoFisher); donkey anti-goat (1:5000, A15999, ThermoFisher); goat anti-mouse (1:20,000, Cat#62-6520, ThermoFisher). After additional washing bands were detected using SuperSignal West Pico PLUS Chemiluminescent Substrate and imaged on a ChemiDoc XRS + Imaging System (BioRad). Uncropped blots with protein ladder (molecular weight markers) are included in the Source Data file or Supplementary Information document.

### Statistics, power calculations, group sizes, and reproducibility

Graph generation and statistical evaluations of repeated measures were conducted by one-way or two-way ANOVA with post hoc independent pair-wise analysis as per Tukey, or by Student’s *t*-tests where appropriate using Prism 9 (GraphPad Software Inc, San Diego, CA). Statistical details of experiments can be found in the figure legends including the statistical tests used and the number of replicative samples. Across all statistical tests significance was defined as *p* value <0.05. Power calculations were performed using G*Power Software V 3.1.9.2. For immunohistochemical quantification analysis and RNA Sequencing, group sizes were calculated to provide at least 80% power when using the following parameters: probability of type I error (alpha) = 0.05, a conservative effect size of 0.25, 2–5 treatment groups with multiple measurements obtained per replicate. All graphs show mean values plus or minus standard error of the means (SEM) as well as individual values as dot plots. All bar graphs are overlaid with dot plots where each dot represents the value for one animal to show the distribution of data and the number (*N*) of animals per group. Injections of NPC and hydrogel formulations were repeated independently at least three times in different colonies of mice across a 2-year period with similar results.

### Principal Component Analysis (PCA) and Cosine similarity (CS)

Principal Component Analysis (PCA), Euclidean distance, and Cosine Similarity (CS) analysis was performed using XLStat (Addinsoft Inc, Long Island City, NY). For PCA, 2 and 3 dimensions were used. PCA data were represented in both Euclidean distance and correlation plots as appropriate. Euclidean distance calculations were derived by assessing the vector magnitude in PCA space of a specific sample referenced to another sample as an initial point. Cosine similarity (CS) measures similarity of samples by comparing the angle between the sample vectors of n-genes and is reported on a scale of 0–1, with 1 being the most similar and 0 being orthogonal.

### Quantification of immunohistochemistry

Immunohistochemical staining intensity quantification was performed on whole brain and spinal cord images derived from the slide scanner or tiled images prepared on the epifluorescence microscope. All images used for each comparable analysis were taken at a standardized exposure time and used the raw/uncorrected intensity setting. Quantification of antibody staining intensity was performed using NIH Image J (1.51) software using the Plot profile (for spinal cord sections) or radial profile angle (for brain sections) plugins using methods developed previously^1^. Total values for IHC stainings were determined by taking the integral (area under the curve) of the plot profile or radial intensity profile. Quantification of co-staining for HA and cell-specific markers Gfap and Pdgfr-α was performed using the RG2B colocalization plugin. Orthogonal (3D) images were prepared using Imaris 9.2 (Bitplane) or Zen 3.1 (Blue Edition) (Zeiss).

### Generation of gene panels

To generate a list of genes enriched in healthy astrocytes we mined eight published healthy astrocyte specific and enriched gene expression datasets from mouse brain and spinal cord. The following datasets were accessed and analyzed from the National Center for Biotechnology Information (NCBI) Gene Expression Omnibus (GEO): GSE199149, GSE94010, GSE84540, GSE114000, GSE52564, GSE18765, GSE66370, GSE103783, GSE35338, GSE153721, GSE100329. Astrocyte enriched genes (AEG) were defined as having a Log_2_FC > 2 compared to all other cells in the sample population or were defined as being detected specifically in astrocytes if derived from single-cell data. Genes designated to the healthy astrocyte gene panel appeared in at least 5 of the 8 lists resulting in a total of 429 genes. The Oligodendrocyte lineage gene (*n* = 112 genes) and neuron gene (*n* = 221 genes) lists were derived from a publicly available list generated from the aggregation of published single-cell datasets (PanglaoDB^42^). We derived the astrocyte reactivity gene panel by analyzing six unique datasets of astrocyte responses to injury in the acute setting available in the literature. The analysis included 3 spinal cord datasets and 3 brain datasets that all derived astrocyte-specific transcriptomic information at acute injury timepoints of less than 7 days after injury. Using the principal criterion that genes needed to be included in at least 3 of the 6 lists, we generated a curated list of 170 genes deemed to be associated with an acute astrocyte response to injury. we also generated an EMT gene panel using the EMT Gene Set Enrichment Analysis (GSEA) data set from the Molecular Signatures Database (MSigdb), which is a publicly available list derived from rodent and human studies. For the EMT gene panel there were 197 genes whose expression level could be assessed in mouse. Comprehensive gene panel information is provided in the Source data files.

### Transcriptomics analysis of RiboTag RNA sequencing data

Analysis of RNA-Seq raw data was performed in Galaxy using standardized workflows. The R1 and R2 FASTQ files from lanes 1 and 2 that were obtained directly from the Illumina NovaSeq S2 run were concatenated and cleaned up using the Trimmomatic tool. Data were then aligned to the M. musculus (mm10) reference genome using the HISAT2 tool applying default parameters. Gene counts from the aligned datasets were performed using the featureCounts tool applying default parameters. Fragments Per Kilobase of transcript per Million mapped reads (FPKM) values were calculated for each gene directly in Excel (Microsoft) using standardized lists of gene lengths and normalization of the count data. Differential expressed gene (DEG) analysis on raw gene count data was conducted using Edge-R in Galaxy applying Benjamini and Hochberg *p* value adjustment and TMM normalization. Across all studies we used a conservative false discovery rate (FDR) cut off <0.01 to define the significance of DEGs and evaluated at least 4 unique samples per experimental group. Gene ontology analyses were performed using Enrichr tool (https://maayanlab.cloud/Enrichr/). Differences in transcript expression across samples were evaluated using data-dimensionality reduction techniques including Principal Component Analysis, Euclidian distance and Cosine Similarity Analysis as described above. Heat maps of DEG data were generated using NG-CHM BUILDER (https://build.ngchm.net/NGCHM-web-builder/). Violin plots of DEGs were generated using Prism 9.

### Analysis of single-nuclei RNA-Seq data

Raw single-nuclei sequencing data were processed using RNA StarSolo on the Galaxy Single Cell Omics platform using M. musculus (mm10) reference genome, 3M-February-2018 barcode whitelist, the gencode vM25 annotation list, and Cell Ranger v3 configure chemistry options. Scanpy tools were used through the Galaxy platform to convert genes, barcodes, and matrix files derived from RNA StarSolo into an AnnData matrix h5ad format. Individual h5ad files for NPC, CNTF, and SPONT samples were generated separately and concatenated (merged) into a single dataset in h5ad format using the Manipulate AnnData tool and applying unique batch designations. The combined dataset was preprocessed to remove mitochondrial genes, filter out genes that were detected in less than 200 cells and filter out low-quality cells that had less than 333 attributed genes. Using Scanpy tools we normalized the data set, identified the 5000 most variable genes across the total population and generated the UMAP projection using the 5000 gene list. Cell clustering was performed on the UMAP data via Scanpy using Louvain clustering algorithms with a resolution of 0.5 which resulted in 11 discrete clusters. Marker genes that defined the clusters were determined using the Scanpy FindMarkers tool applying default parameters. Clusters were assigned NPC, astrocyte or oligodendroglia lineage designation on the basis of expression of known marker genes for those specific cells. Violin plots of gene expression distribution within clusters was also performed using Scanpy. The percentage of cells from each condition detected within each cluster was determined from the batch designations assigned upon merging of the datasets.

### Reporting summary

Further information on research design is available in the Nature Research Reporting Summary linked to this article.

## Supplementary information

Supplementary Information

Reporting Summary

## Data Availability

RiboTag RNA-Seq and Single-cell RNAseq data have been deposited at Gene Expression Omnibus (GEO) and are publicly available with Accession numbers GSE194319. Data users can retrieve raw sequencing data at https://www.ncbi.nlm.nih.gov/geo/query/acc.cgi?acc=GSE194319. All data generated for this study are included in the main and supplementary figures. For all quantitative figures, files of source data of individual values as well as the results of statistical tests are provided with the paper. Source Data are provided as a Source Data file. Other data that support the findings of this study are available on request from the corresponding author.

## Source data

Source Data

## References

[1] O’Shea TM (2020). Foreign body responses in central nervous system mimic natural wound responses and alter biomaterial functions. Nat. Commun..

[2] Dias DO (2021). Pericyte-derived fibrotic scarring is conserved across diverse central nervous system lesions. Nat. Commun..

[3] Bardehle S (2013). Live imaging of astrocyte responses to acute injury reveals selective juxtavascular proliferation. Nat. Neurosci..

[4] Burda JE, Sofroniew MV (2014). Reactive gliosis and the multicellular response to CNS damage and disease. Neuron.

[5] Sofroniew MV (2015). Astrocyte barriers to neurotoxic inflammation. Nat. Rev. Neurosci..

[6] Bunge RP, Puckett WR, Hiester ED (1997). Observations on the pathology of several types of human spinal cord injury, with emphasis on the astrocyte response to penetrating injuries. Adv. Neurol..

[7] Berry M (1983). Deposition of scar tissue in the central nervous system. Acta Neurochir. Suppl..

[8] Norenberg MD, Smith J, Marcillo A (2004). The pathology of human spinal cord injury: defining the problems. J. Neurotrauma.

[9] Dorrier CE (2021). CNS fibroblasts form a fibrotic scar in response to immune cell infiltration. Nat. Neurosci..

[10] O’Shea TM, Burda JE, Sofroniew MV (2017). Cell biology of spinal cord injury and repair. J. Clin. Invest.

[11] Carmichael ST, Kathirvelu B, Schweppe CA, Nie EH (2017). Molecular, cellular and functional events in axonal sprouting after stroke. Exp. Neurol..

[12] Fernández-Klett F, Priller J (2014). The fibrotic scar in neurological disorders. Brain Pathol..

[13] Chen B (2018). Reactivation of dormant relay pathways in injured spinal cord by KCC2 manipulations. Cell.

[14] Courtine G (2008). Recovery of supraspinal control of stepping via indirect propriospinal relay connections after spinal cord injury. Nat. Med..

[15] Assinck P, Duncan GJ, Hilton BJ, Plemel JR, Tetzlaff W (2017). Cell transplantation therapy for spinal cord injury. Nat. Neurosci..

[16] Blakemore WF, Olby NJ, Franklin RJM (1995). The use of transplanted glial cells to reconstruct glial environments in the CNS. Brain Pathol..

[17] Björklund A, Stenevi U (1984). Intracerebral neural implants: neuronal replacement and reconstruction of damaged circuitries. Annu Rev. Neurosci..

[18] Lindvall O, Barker RA, Brüstle O, Isacson O, Svendsen CN (2012). Clinical translation of stem cells in neurodegenerative disorders. Cell Stem Cell.

[19] Lu P (2012). Long-distance growth and connectivity of neural stem cells after severe spinal cord injury. Cell.

[20] Dulin JN (2018). Injured adult motor and sensory axons regenerate into appropriate organotypic domains of neural progenitor grafts. Nat. Commun..

[21] Houlé JD, Reier PJ (1988). Transplantation of fetal spinal cord tissue into the chronically injured adult rat spinal cord. J. Comp. Neurol..

[22] Björklund A, Stenevi ULF, Svendgaard N-A (1976). Growth of transplanted monoaminergic neurones into the adult hippocampus along the perforant path. Nature.

[23] Cummings BJ (2005). Human neural stem cells differentiate and promote locomotor recovery in spinal cord-injured mice. Proc. Natl Acad. Sci. USA.

[24] Svendsen CN (1997). Long-term survival of human central nervous system progenitor cells transplanted into a rat model of Parkinson’s disease. Exp. Neurol..

[25] Vroemen M, Aigner L, Winkler J, Weidner N (2003). Adult neural progenitor cell grafts survive after acute spinal cord injury and integrate along axonal pathways. Eur. J. Neurosci..

[26] Karimi-Abdolrezaee S, Eftekharpour E, Wang J, Morshead CM, Fehlings MG (2006). Delayed transplantation of adult neural precursor cells promotes remyelination and functional neurological recovery after spinal cord injury. J. Neurosci..

[27] Cao QL (2001). Pluripotent stem cells engrafted into the normal or lesioned adult rat spinal cord are restricted to a glial lineage. Exp. Neurol..

[28] Kokaia Z, Llorente IL, Carmichael ST (2018). Customized brain cells for stroke patients using pluripotent stem cells. Stroke.

[29] Nori S (2011). Grafted human-induced pluripotent stem-cell-derived neurospheres promote motor functional recovery after spinal cord injury in mice. Proc. Natl Acad. Sci. USA.

[30] Kadoya K (2016). Spinal cord reconstitution with homologous neural grafts enables robust corticospinal regeneration. Nat. Med..

[31] Sareen D (2014). Human induced pluripotent stem cells are a novel source of neural progenitor cells (iNPCs) that migrate and integrate in the rodent spinal cord. J. Comp. Neurol..

[32] Akhtar AA (2018). Inducible expression of GDNF in transplanted iPSC-derived neural progenitor cells. Stem Cell Rep..

[33] Fischer I, Dulin JN, Lane MA (2020). Transplanting neural progenitor cells to restore connectivity after spinal cord injury. Nat. Rev. Neurosci..

[34] Sanz E (2009). Cell-type-specific isolation of ribosome-associated mRNA from complex tissues. Proc. Natl Acad. Sci. USA.

[35] Wollenberg AL (2018). Injectable polypeptide hydrogels via methionine modification for neural stem cell delivery. Biomaterials.

[36] Roybon L (2013). Human stem cell-derived spinal cord astrocytes with defined mature or reactive phenotypes. Cell Rep..

[37] Kuegler PB (2010). Markers of murine embryonic and neural stem cells, neurons and astrocytes: reference points for developmental neurotoxicity testing. Altex.

[38] Pollard SM, Conti L, Sun Y, Goffredo D, Smith A (2006). Adherent neural stem (NS) cells from fetal and adult forebrain. Cereb. Cortex.

[39] Johe KK, Hazel TG, Muller T, Dugich-Djordjevic MM, McKay RD (1996). Single factors direct the differentiation of stem cells from the fetal and adult central nervous system. Genes Dev..

[40] Rajan P, McKay RDG (1998). Multiple routes to astrocytic differentiation in the CNS. J. Neurosci..

[41] Hsieh J (2004). IGF-I instructs multipotent adult neural progenitor cells to become oligodendrocytes. J. Cell Biol..

[42] Franzén O, Gan L-M, Björkegren JLM (2019). PanglaoDB: a web server for exploration of mouse and human single-cell RNA sequencing data. Database.

[43] Kamphuis W (2012). GFAP isoforms in adult mouse brain with a focus on neurogenic astrocytes and reactive astrogliosis in mouse models of Alzheimer disease. PLoS ONE.

[44] Iser IC, Lenz G, Wink MR (2019). EMT-like process in glioblastomas and reactive astrocytes. Neurochem. Int..

[45] Vivinetto AL (2020). Zeb2 is a regulator of astrogliosis and functional recovery after CNS injury. Cell Rep..

[46] Klatt Shaw D (2021). Localized EMT reprograms glial progenitors to promote spinal cord repair. Dev.Cell.

[47] Shen F, Song C, Liu Y, Zhang J, Wei Song S (2019). IGFBP2 promotes neural stem cell maintenance and proliferation differentially associated with glioblastoma subtypes. Brain Res..

[48] Haas C, Neuhuber B, Yamagami T, Rao M, Fischer I (2012). Phenotypic analysis of astrocytes derived from glial restricted precursors and their impact on axon regeneration. Exp. Neurol..

[49] Zhang Y (2016). Purification and characterization of progenitor and mature human astrocytes reveals transcriptional and functional differences with mouse. Neuron.

[50] Bologa L, Cole R, Chiappelli F, Saneto RP, De Vellis J (1988). Expression of glial fibrillary acidic protein by differentiated astrocytes is regulated by serum antagonistic factors. Brain Res..

[51] Brennan FH, Anderson AJ, Taylor SM, Woodruff TM, Ruitenberg MJ (2012). Complement activation in the injured central nervous system: another dual-edged sword?. J. Neuroinflammation.

[52] Fedoroff S, Hall C (1979). Effect of horse serum on neural cell differentiation in tissue culture. In Vitro.

[53] Schuster R, Rockel JS, Kapoor M, Hinz B (2021). The inflammatory speech of fibroblasts. Immunol. Rev..

[54] Halder SK, Beauchamp RD, Datta PK (2005). A specific inhibitor of TGF-beta receptor kinase, SB-431542, as a potent antitumor agent for human cancers. Neoplasia.

[55] Morishita R (2007). Expression of smooth muscle cell-specific proteins in neural progenitor cells induced by agonists of G protein-coupled receptors and transforming growth factor-beta. J. Neurochem.

[56] O’Shea, T. M. et al. *Smart Materials for Tissue Engineering: Applications* 529-557 (The Royal Society of Chemistry, 2017).

[57] Kang SH, Fukaya M, Yang JK, Rothstein JD, Bergles DE (2010). NG2+ CNS glial progenitors remain committed to the oligodendrocyte lineage in postnatal life and following neurodegeneration. Neuron.

[58] Wanner IB (2013). Glial scar borders are formed by newly proliferated, elongated astrocytes that interact to corral inflammatory and fibrotic cells via STAT3-dependent mechanisms after spinal cord injury. J. Neurosci..

[59] Tyzack GE (2017). A neuroprotective astrocyte state is induced by neuronal signal EphB1 but fails in ALS models. Nat. Commun..

[60] Lois C, Alvarez-Buylla A (1994). Long-distance neuronal migration in the adult mammalian brain. Science.

[61] Anderson MA (2018). Required growth facilitators propel axon regeneration across complete spinal cord injury. Nature.

[62] Shi X (2021). Stroke subtype-dependent synapse elimination by reactive gliosis in mice. Nat. Commun..

[63] Zukor K (2013). Short hairpin RNA against PTEN enhances regenerative growth of corticospinal tract axons after spinal cord injury. J. Neurosci..

[64] Sirko S (2015). Astrocyte reactivity after brain injury—: the role of galectins 1 and 3. Glia.

[65] Anderson MA (2016). Astrocyte scar formation aids central nervous system axon regeneration. Nature.

[66] Wu YE, Pan L, Zuo Y, Li X, Hong W (2017). Detecting activated cell populations using single-cell RNA-Seq. Neuron.

[67] Burda JE (2022). Divergent transcriptional regulation of astrocyte reactivity across disorders. Nature.

[68] Bjørnbak C, Brøchner CB, Larsen LA, Johansen JS, Møllgård K (2014). Brain barriers and a subpopulation of astroglial progenitors of developing human forebrain are immunostained for the glycoprotein YKL-40. J. Histochem. Cytochem..

[69] Cubas-Núñez L (2021). Potential Role of CHI3L1+ astrocytes in progression in MS. Neurol. Neuroimmunol. Neuroinflamm.

[70] Brøchner CB, Holst CB, Møllgård K (2015). Outer brain barriers in rat and human development. Front. Neurosci.

[71] Khazaei M (2020). GDNF rescues the fate of neural progenitor grafts by attenuating Notch signals in the injured spinal cord in rodents. Sci. Transl. Med..

[72] Buffo A (2008). Origin and progeny of reactive gliosis: a source of multipotent cells in the injured brain. Proc. Natl Acad. Sci. USA.

[73] Pous L (2020). Fibrinogen induces neural stem cell differentiation into astrocytes in the subventricular zone via BMP signaling. Nat. Commun..

[74] Schiweck J (2021). Drebrin controls scar formation and astrocyte reactivity upon traumatic brain injury by regulating membrane trafficking. Nat. Commun..

[75] Wichterle H, Lieberam I, Porter JA, Jessell TM (2002). Directed differentiation of embryonic stem. Cells into Mot. Neurons Cell.

[76] Bush TG (1999). Leukocyte infiltration, neuronal degeneration, and neurite outgrowth after ablation of Scar-forming, reactive astrocytes in adult transgenic mice. Neuron.

