# Supplementary material for: Lesion environments direct transplanted neural progenitors towards a wound repair astroglial phenotype in mice
Source: Nat Commun. 2022 Sep 28;13:5702. doi: 10.1038/s41467-022-33382-x (PMC9519954; doi:10.1038/s41467-022-33382-x)

Supplemental Information

Lesion environments direct transplanted neural progenitors towards a wound repair astroglial phenotype in mice

O'Shea, T.M.[1,2]*, Ao, Y.[1], Wang, S.[1], Wollenberg, A.L.[3,4], Kim J.H.[1], Ramos Espinoza, R.A.[2], Czechanski, A.[5], Reinholdt, L.G.[5], Deming, T.J.[3,4], and Sofroniew, M.V.[1]*

1. Department of Neurobiology, David Geffen School of Medicine, University of California, Los Angeles, CA, 90095-1763, USA.
2. Department of Biomedical Engineering, Boston University, Boston, MA, 02215-2407, USA.
3. Department of Chemistry and Biochemistry, University of California Los Angeles, Los Angeles, CA, 90095-1600, USA.
4. Department of Bioengineering, University of California Los Angeles, Los Angeles, CA, 90095-1600, USA.
5. The Jackson Laboratory, Bar Harbor, ME 04609, USA.

*Correspondence: toshea@bu.edu (T.M.O); sofroniew@mednet.ucla.edu (M.V.S.)

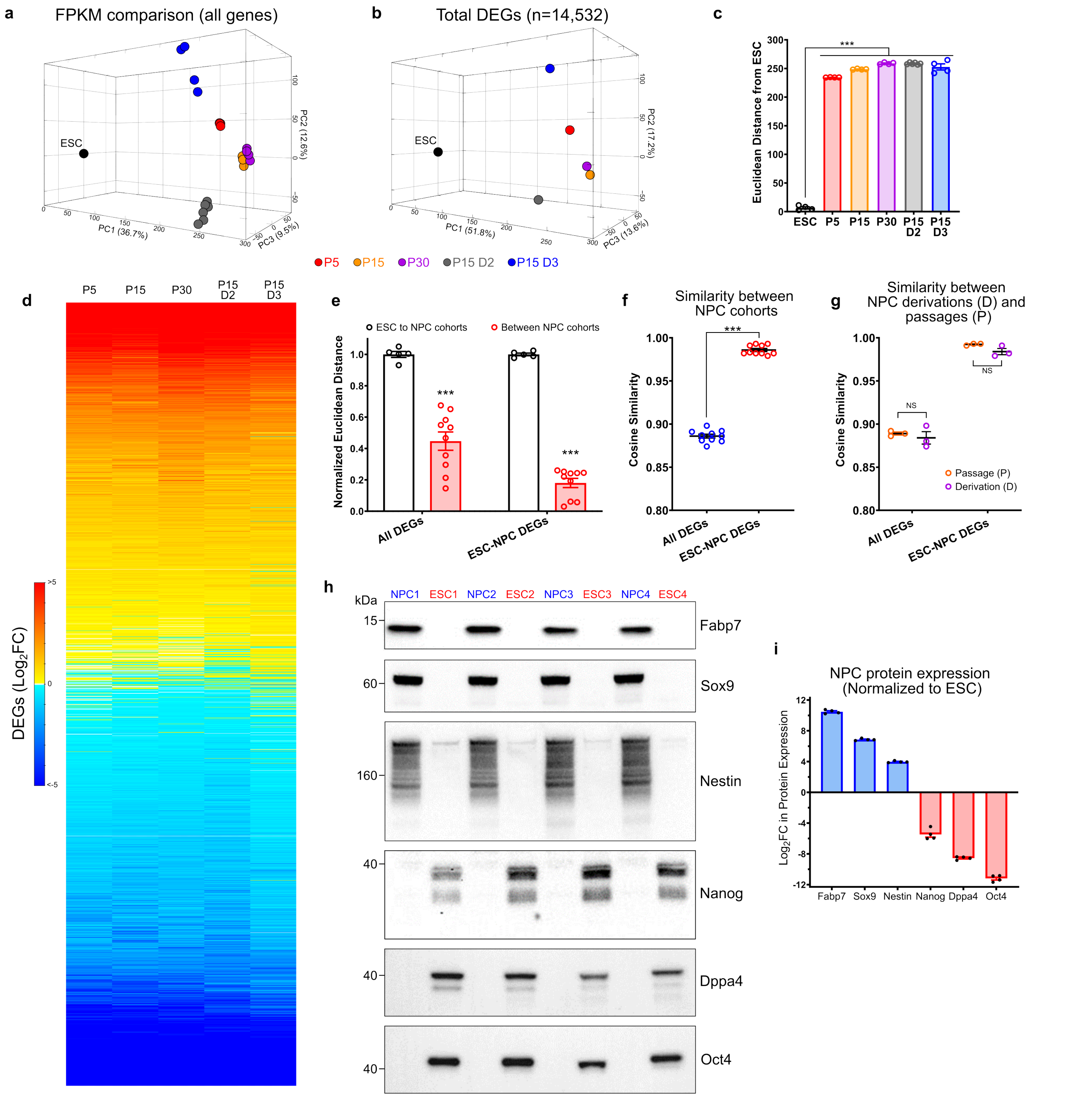

Supplementary Fig. 1 | Neural induction and expansion of RiboTag mESC derives a reproducible and stable NPC line. a PCA of NPC samples from different derivations (d) and passages (P) comparing all expressed genes (genes measured with an FPKM >0.1 in at least one sample) referenced to ESC sample average. n=4 per group for all groups. **b** PCA of NPC samples from different derivations (d) and passages (P) comparing all DEGs (DEG defined as having an FDR≤0.01) for NPC samples referenced to ESC. n=4 per group for all groups. Total number of DEGs used in PCA = 14,352. **c** Euclidean distance comparisons for ESC and NPC samples. Euclidean distance equates to the vector magnitude in PCA space of NPC samples referenced to mean of ESC samples as the initial point, using FPKM values for all detected genes. n=4, *** p-value < 0.0001, One way ANOVA with Tukey multiple comparison test. **D** Heatmap of all DEGs referenced to ESC for NPC at different Passages (P) and derivation (D). **e** Normalized Euclidean distance evaluations comparing the scaled vector lengths between ESC and NPC samples with the scaled vector lengths between individual NPC samples from different Passages (P) and derivation (D) for all DEGs (n=14,532 genes) or for ESC-NPC gene panel DEGs (n=88 genes). N=5 and 10, *** p-value < 0.0001, Two way ANOVA with Tukey multiple comparison test. **f** Cosine similarity comparisons for NPC samples from different Passages (P) and derivation (D) using all DEGs (n=14,532 genes) or ESC-NPC gene panel DEGs (n=88 genes). N=10 *** p-value < 0.0001, two-tailed, unpaired Student's t-test. **g** Cosine similarity comparisons for NPC samples comparing by different Passage number (P) or derivation (d). N=3 per group, Not significant (NS), Two way ANOVA with Tukey multiple comparison test. **h** Western blot of NPC and ESC samples (N=4 samples per cell type) for canonical NPC markers Fabp7, Sox9, Nestin as well as canonical ESC makers Nanog, Dppa4 and Oct4. **i** Quantification of Western blots measuring protein expression of canonical NPC and ESC markers (N=4 samples per cell type). Expression levels in all samples were referenced to total protein that was quantified by SYPRO Ruby Protein Blot Stain. Mean NPC expression levels were referenced to mean ESC levels. All bar graphs are mean ± s.e.m. with individual datapoints overlayed.

Astrocyte Gene Panel

Aldoc	Papss2	Hist1h2ac	Aass	Fam181a	Tmem176b
Apoe	Paqr6	Fzd9	Lfng	Atp13a4	Trp53bp2
Aldh1l1	Hyal1	Cyr61	Gli2	Rfx4	Acsm5
Htra1	Vcam1	Cpq	Fam181b	BC064078	Ctso
Slc1a3	Pld2	Aldh2	Dmp1	Car5b	Vim
Sox9	Ppp1r3c	Acaa2	Mmp14	Etv4	S100b
Ntsr2	Tmem51	Myh15	Ppp1r3g	P4ha3	Fam107a
Gja1	Scg3	Exoc3l4	Hsd11b1	Dag1	Apln
S1pr1	Elf5	Tmco4	Kctd14	Slc4a4	Mapkapk3
Fgfr3	Phka1	Bdh2	Ezr	Dio2	Stk17b
Pla2g7	Cideb	Rbm46	Fgfrl1	Fabp7	Lrp10
Slc1a2	Emp2	Etnppl	Slc2a10	Igsf1	Pnpla7
Lcat	Ednrb	Pdlim5	Pbxip1	Fkbp10	Tmem100
Cldn10	Npas3	Irak2	Rapgef3	Slc12a4	Acadm
Acsl6	Pon2	Smox	Steap3	Mfge8	Itga6
Entpd2	Slc15a2	Renbp	Fxyd1	Gdf10	Ech1
Agt	Sash1	Aldh4a1	Cyp2j9	Lgr6	Gpc6
Gpr37l1	Adhfe1	Mtss1l	Nwd1	Serhl	Elovl5
Slc6a11	Gm2a	2310022B05Rik	Id3	Itga7	Dapp1
Acsbg1	Bmpr1b	Sugct	Sdc4	Oplah	Gm17455
Fads2	Scd2	Myo10	Acss1	Ip6k3	Kank1
Plxnb1	Cd81	Phyh	Cth	Slc13a3	Apoc1
Clu	Nat1	Plin2	Sardh	Pygm	Hist1h4j
Slc39a12	Smpdl3a	Pdlim4	Kirrel2	Ltbr	Cables1
Cbs	Oat	Prss50	Pm20d1	Cyp2d22	Acad11
Slc27a1	Rnase1	Id1	Ltbp1	Sdsl	Lonrf3
Gfap	Pxmp2	Erbb2	Msi1	Gstt1	Decr1
Atp1a2	Tnfrsf19	Loxl3	Fam69c	Heph	Acacb
Srebf1	Fjx1	Itpr2	Alpl	Selenbp1	Baalc
Pygb	Als2cl	Cnn3	Axl	Fzd2	Fgd6
Slc7a10	Eno1b	Aldh7a1	Hspa2	Tdgf1	Smo
Itih3	Gm973	Hrh1	Mt2	Cdc42ep4	Scd1
Gstm1	Ptprz1	Notch2	Sox2	Myoc	As3mt
Sparcl1	Tlr3	Abhd4	Slc13a5	Plcd1	Lrrc8a
Ndrg2	Myo6	Tmem176a	Phkg1	Plcb3	Inppl1
Cpe	Luzp2	Aldh1a1	Slc25a18	Klhdc7a	Cybrd1
Rgma	Timp4	Bcar3	Pard3b	Asrgl1	Stat5a
Tril	Pdpn	Thnsl2	2810459M11Rik	Hes5	Tead1
Tst	Myom3	Necap2	Tnc	Fzd10	Ston2
Nkain4	Mgst1	Acad12	Fam20a	Tnfrsf1a	Add3
Prodh	Serpine2	Rftn2	Rlbp1	Arhgef19	Gpr179
Dbx2	Acsl3	Hgf	F3	Mfsd2a	Yap1
Sfxn5	Ntrk2	Epx	Pdk4	Cntfr	Tspan7
Mmd2	Mt1	Btd	Ephx2	Gstt3	4921530L21Rik
Abhd3	Rarres1	Arhgef26	Lgi4	Smpd2	Rnf182
Timp3	Tnfaip8	Oaf	Agl	Gpam	Ptch1
Atp1b2	Nrarp	Eci1	Psd2	Gpc5	Kcnj16
Hepacam	Gabrg1	Acads	Grin2c	Idh2	Hbegf
Gjb6	Rgcc	Egfr	Sema4b	Slc8b1	Zfp36l2
Slc1a4	Soat1	Lrrc2	Paqr8	Lgals3bp	A730056A06Rik
Aqp4	Adk	Adcyap1r1	Slc25a34	S100a1	Laptm4a
Lxn	Lix1	Tns3	Palld	Chil1	Gm266
Glud1	Gdpd2	Slc2a12	Maob	Plekho2	Bbox1
Ncan	Ckb	Grm3	Cst3	Dspp	Ptn
Ttyh1	Ndp	Lpar4	Tlcd1	Sfrp5	A330048O09Rik
Lpin3	Smyd1	Fam213a	Cd63	Btbd17	Rgl3
Slc9a3r1	Rorb	Acadl	Egfl6	Gstk1	Mr1
Slc38a3	Hadhb	Abca1	Prdx6	Suclg2	Mdk
Kcne1l	4932438H23Rik	Hacl1	Ppara	Eps8	Frem2
Cyp4f14	Mamdc2	3110082J24Rik	Amot	Fads1	Gli3
A2m	Sox21	Gprc5b	Mertk	Padi2	Rgs20
Lrp4	Calr4	5930403L14Rik	Slc14a1	Olfr287	Id4
AI464131	Nek8	Eva1a	Lrig1	Tekt4	Aqp9
Mlc1	Msx2	Evc	Cd38	Sparc	Ttpa
Rdh5	Rbpms2	Fmo5	Kcnj10	Snta1	Pax6
Bcan	Echdc3	Elovl2	Ucp3	Cpt1a	Cbr3
Acsf2	D630039A03Rik	Dock1	Mcc	Sh3pxd2b	Glycam1
C4b	Slc6a1	A730036I17Rik	Ddo	Igdcc4	Nqo1
Gldc	Gm11627	Thrsp	Adora2b	Cyp4v3	Prkd1
Acot11	Micalcl	Ddah1	Slc7a2	Glipr2	Cxcl14
Slc2a4	Megf10	Mid1ip1	Cyp4f15	Ccdc8	Nat8
Pttg1ip	Mt3	Arhgap5			

Oligodendroglia

Gpr17	Tmem88B
Neu4	Bace1
Fa2H	Vcan
Ninj2	Olig3
Tmem163	Gpr62
C1Ql1	Slc25A38
Opalin	S1Pr5
Enpp6	Aspa
Gpr37L1	Zdhhc9
Gjc3	Tmem125
Sox10	Hapln2
Plp1	Gjb1
Tmem100	Ermn
Dusp15	Klk6
Omg	Cldn14
Pcdh15	Sp7
Mbp	Prkcz
Nkx6-2	Klhl2
Tnr	Olig2
Sgk2	Kif13B
Ptgds	Sec14L5
Hepacam	Galc
Cldn11	Fyn
St18	Cspg5
Cdo1	Slc25A47
Ephb1	Fasn
Nfe2L3	Eml1
Gpr37	Tmem63A
Cnp	Pde8A
Myrf	Dpy19L1
Enpp2	Il33
Arsg	Plekhh1
Tspan15	Slc25A29
Nnat	Nipal4
Cntf	Vldlr
Pllp	Epn2
Mag	Creb5
Aldoc	Anln
Bcas1	Etv5
Fgfr2	Trf
Pnpla2	Pou3F1
Kif6	
Lhfpl3	
Itgb4	
Mog	
Efnb3	
Kcnip3	
Dbndd2	
Tyro3	
Hdac11	
Elovl7	
Matn4	
Ascl1	
Sox8	
Sema4D	
Cspg4	
Arrdc2	
Olig1	
Itpr2	
Myo1D	
Mcam	
Adamts4	
Pex5L	
Npc1	
Pdgfra	
Slc25A19	
Sgk3	
Gjc2	
Gamt	
Lad1	
Ddc	

Neuronal

Gabbr2	Nicn1	Atp6V1H
Miat	Cxcr5	Cxcl1
Cck	Frrs1L	Nrsn2
Scrg1	Magee1	Zcchc7
Fmo1	Atp6V0A1	Cbarp
Cpne6	Scn8A	Spock3
Elavl3	Gprasp2	Cntnap2
Rbfox3	Safb2	Arhgdig
Ppp1R1B	Disp2	Zcchc12
Slc11A1	Rhbdd2	Tmem130
St8Sia3	Flywch1	Slc30A9
Dnm3	Cnrip1	Tmem59L
Cd59A	Rb1Cc1	Slc12A5
Pcsk2	Mecp2	Vsnl1
Slc1A1	Wdr60	Resp18
Npy	Tro	Snhg11
Nwd2	Myo5A	Snap25
Hap1	Rnpc3	Meg3
Chodl	Dipk1b	Pnisr
Slc6A17	Dtd1	Safb
Abat	Cdk5	Sfswap
Dlgap1	Rere	Rnf220
B3Gat2	Zmynd11	Dlg4
Sgip1	Prph	Ptpn11
Syne1	Syt5	Ogfod1
Dner	Tubb3	Gpatch8
Prune2	Car10	Necab3
Cacna2D2	Icam5	Zfp445
Dusp26	Shisa2	Chd6
Luzp2	Ndn	Nbea
Rasgrf1	Ptk2B	Fam181B
Vgf	Aqp1	Map7D2
Pcp4	Pnck	Gnai1
Eno2	Necab2	Gria4
Dzank1	Bex1	Trip11
Stmn2	Ccdc28B	Scn1A
Pclo	Stx1A	Cspp1
Tceal3	Pqbp1	Ppp1R14C
Ifit3	Igf1	Napg
Ddn	Epo	Copg1
Cacnb3	Ghsr	Arid4A
Tmem179	Chat	Gnl3L
Ppp1R9A	Omp	Fbxw7
Rgs17	Agrp	Pabpn1
Celf6	Th	Cacna2D1
Tmem191C	Aim2	Gad1
Rora	Csf3	Scn2B
Eml5	Avp	Zic1
Cplx1	Hoxc8	Gng2
Cacnb4	Sacs	Kcnma1
Matk	Tac2	Kcnk1
Hdac11	Nefh	Rgs7Bp
Akap8L	Tmem91	Aak1
Ncald	Mirg	Tesc
Scamp1	Ube2D2A	Ckmt1
Dgkb	Cacng2	Slc36A4
Ryr2	Ctxn2	Acsl4
Cmip	Tceal6	Chn1
Zrsr1	Hpca	Rims3
Pacsin1	Slc17A6	Synpr
Prox1	Pnmal1	Kcnc1
Prmt2	Nrn1	Fbln2
Camk2N1	Tff3	Pde1A
Rin1	Dync1I1	Matn2
Gpr162	Gabrg2	Calb1
Clstn3	Tceal5	Gria3
Kmt2C	Slc32A1	Gabra1
Bex2	Nefm	Grm5
Slc17A7	Nmt1	Isl1
Cttnbp2	Rit2	Nrgn
Calb2		

Progenitor/ Proliferation
Fzd9
Lmo3
Wnt5a
Bmi1
Slc1a3
Traf4
Efnb1
Serpine2
Zic1
Metrn
Lhx2
Hjurp
Sox11
Nes
Mki67
Kif2c
Nptx1
Cenpf
Ube2c
Top2A
Fzd1
Cenpk
Cdk1
Lgals1
Ccnd1
Cenpa
Igfbp3
Tnc
Pou3f1
Ccnd2
Igfbp2
Igfbp4

Supplementary Table. 1 | Gene panels for Astrocyte lineage, Oligodendrocyte lineage, Neuronal lineage cells and genes associated with progenitor/proliferation state. Healthy Astrocyte gene panel (n=429) was derived from meta-analysis of archival datasets (see Supplementary Fig. 2 and Source data for details). Oligodendrocyte lineage and Neuronal lineage gene panels were derived using PanglaoDB single cell datasets.

Step 1 — Access astrocyte RNA-Seq Data (n=8 data sets)

A Our work - **Sofroniew Lab (UCLA)** - Healthy mouse Spinal Cord from 73.12 GFAP (**1**) and Aldh1l1-CreERT2 - RiboTag (**2**)

B **Khahk Lab (UCLA)** - Healthy mouse Striatum (**3**), Hippocampus (**4**), Cortex (**5**) Aldh1l1-CreERT2 - RiboTag

C **Holt Lab (Leuven)** - Healthy mouse Brain - single cell RNA-Seq (**6**)

D **Barres Lab (Stanford)** - Healthy mouse Brain - immunopanned (**7**)

E **PanglaoDB** gene list for Astrocytes - aggregated database of single cell datsets (**8**)

Step 2 — Define astrocyte enriched genes (AEG)

A AEG = Log_2FC of RiboTag IP vs flow-through > 2.0 (FDR < 0.01)

B AEG = Log_2FC of RiboTag IP vs Input > 2.0 (FDR < 0.01)

C AEG = Genes that appear on "common" astrocyte gene list

D AEG = Log_2FC > 2.0 for Astrocytes compared with average expression for all other immunopanned cell types (FDR < 0.01)

E AEG = all genes on currated Astrocyte gene list

Data Set	1	2	3	4	5	6	7	8
**Total number of genes**	1386	1456	1096	873	899	345	944	62

Step 3 — Identify genes appearing in at least 5 of 8 lists

Total genes in Astrocyte gene list = 429 (Full gene list in source data)

Genes on 8 of 8 lists (n=15)

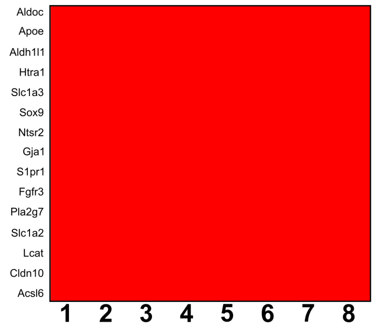

Genes on 7 of 8 lists (n=40)

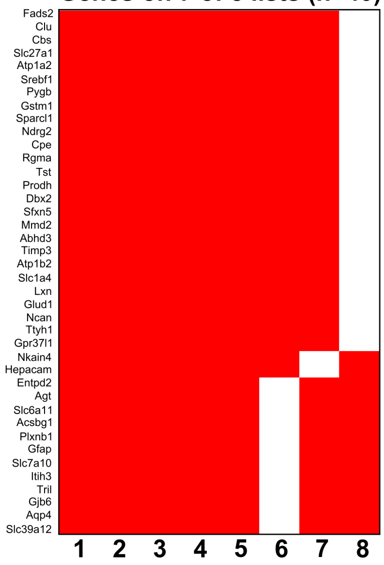

Genes on 6 of 8 lists (n=169)

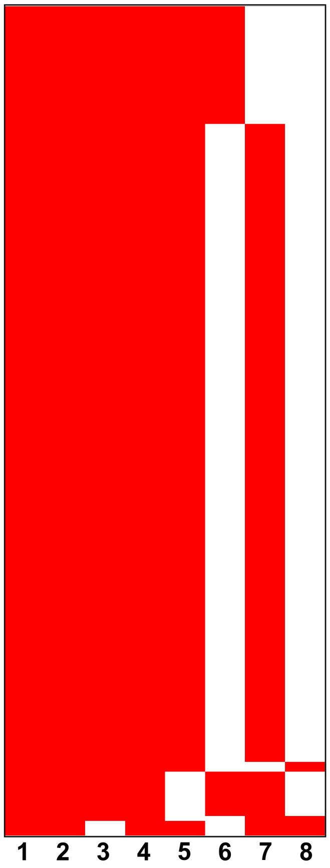

Genes on 5 of 8 lists (n=205)

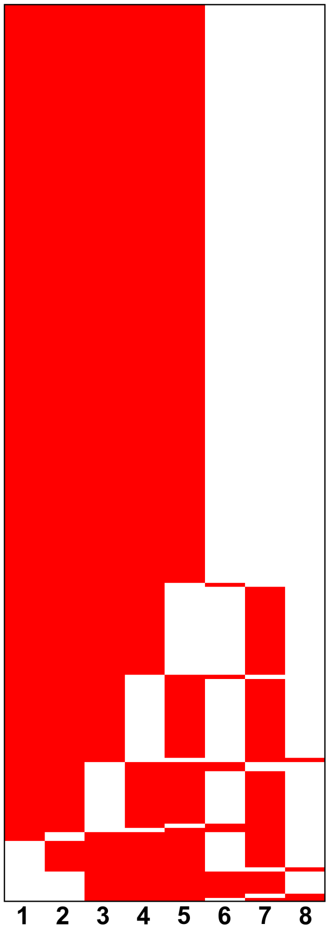

Supplementary Fig. 2 | Overview of workflow for generating Astrocyte gene panel by meta-analysis of archival datasets.

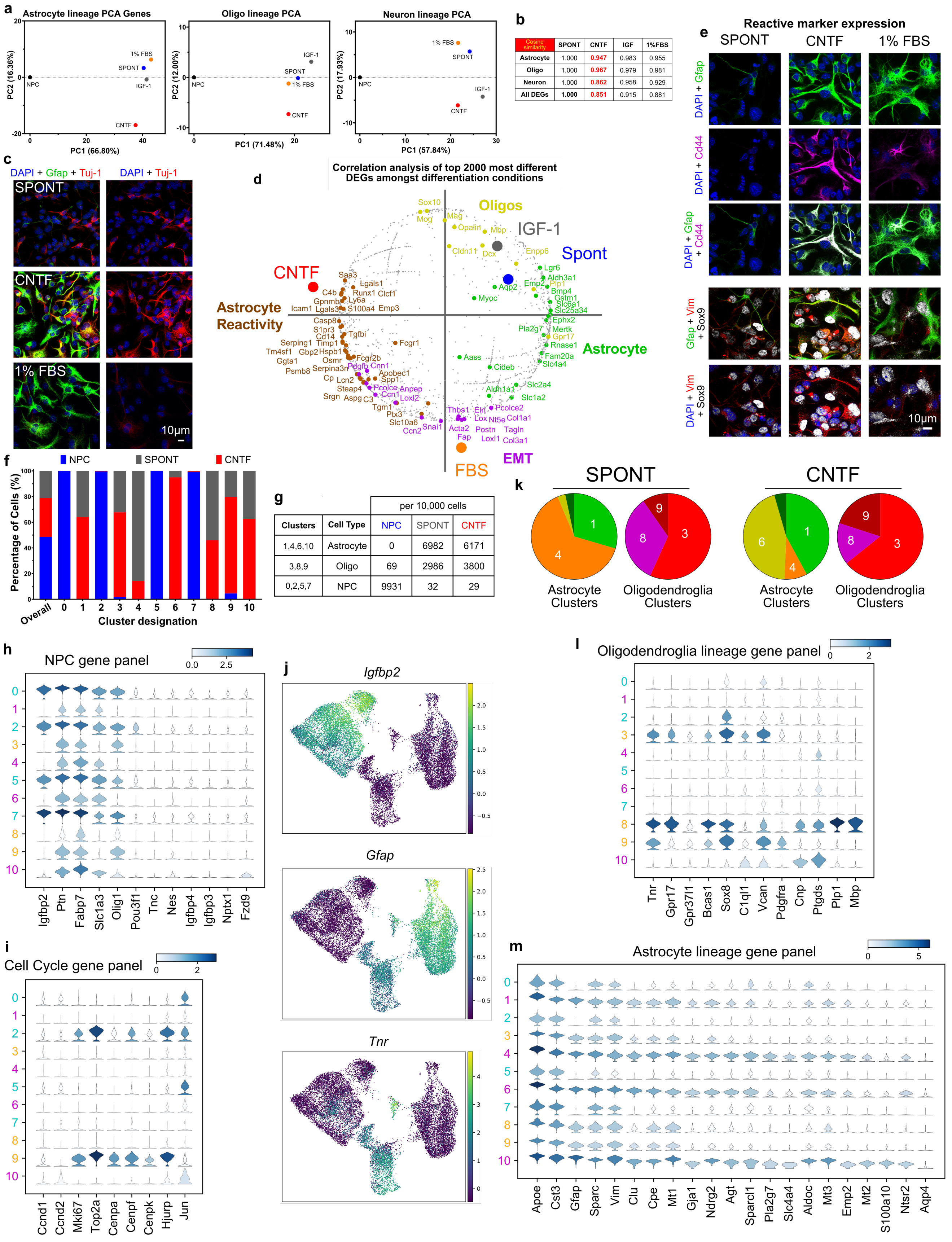

Cosine similarity	SPONT	CNTF	IGF	1%FBS
Astrocyte	1.000	0.947	0.983	0.955
Oligo	1.000	0.967	0.979	0.981
Neuron	1.000	0.862	0.958	0.929
All DEGs	1.000	0.851	0.915	0.881

		per 10,000 cells		
Clusters	Cell Type	NPC	SPONT	CNTF
1,4,6,10	Astrocyte	0	6982	6171
3,8,9	Oligo	69	2986	3800
0,2,5,7	NPC	9931	32	29

Supplementary Fig. 3 | NPC differentiate into astrocyte and oligodendroglia lineage cells *in vitro* with phenotype guided by specific molecules. a PCA for the differentiation conditions using the healthy Astrocyte, Oligodendroglia lineage and Neuron gene panels. **b** Table of cosine similarity comparisons for the differentiation conditions referenced to SPONT differentiation and analyzed using the neural cell type gene panels or all DEGs. CNTF treated samples were least similar to the SPONT differentiation across all analyzes. **c** ICC staining of differentiated cell conditions SPONT, CNTF and 1%FBS for pan neuronal marker Tuj-1 and counter stained with Gfap and DAPI. **d** Correlation analysis of the top 2000 most differential regulated DEGs amongst the 4 differentiation conditions by correlation PCA. CNTF treated samples correlated strongest with astrocyte reactivity genes, FBS treated samples with EMT genes and SPONT and IGF-1 correlated with higher expression of Oligodendroglia lineage and healthy astrocyte lineage genes. **e** ICC staining of differentiated cell conditions SPONT, CNTF and 1%FBS for reactivity markers Cd44 and Vimentin (Vim) and counter stained with Gfap

and DAPI. **f** Percentage of cells from NPC, CNTF and SPONT conditions in each of the 11 clusters (0-10) derived from single cell analysis. **g** Table of cell type groupings for the single cell analysis indicating the number of cells per 10,000 for each cell type from each condition. **h** Violin Plots displaying distribution of expression of canonical NPC genes across all cells in the each of cluster designations. Color scale indicates median expression values. **i** Violin Plots displaying distribution of expression of canonical cell cycle or proliferation genes across all cells in the each of cluster designations. Color scale indicates median expression values. **j** Gene expression values across all cells arranged in UMAP space for the genes that most uniquely define NPC (*Igfpb2*), Astrocyte (*Gfap*) and Oligodendroglia (*Tnr*) lineage cells. **k** Pie charts displaying the percentage of each astrocyte and oligodendroglia lineage cluster for the SPONT and CNTF treated samples. While the distribution of oligodendroglia lineage clusters was comparable across the two differentiation conditions, SPONT showed predominant cluster 4 astrocytes whereas CNTF showed predominant cluster 6 astrocytes. **l** Violin Plots displaying distribution of expression of oligodendroglia lineage genes across all cells in the each of cluster designations. Color scale indicates median expression values. **m** Violin Plots displaying distribution of expression of astrocyte lineage genes across all cells in the each of cluster designations. Color scale indicates median expression values.

Astrocyte Genes	SC Astros	SCI Astros	Spont	CNTF	IGF-1	1%FBS	NPC
Apoe	14805.1	18634.8	9949.9	10067.7	13946.7	12167.7	30.7
Mt1	7030.8	14600.0	2645.2	2353.3	4383.2	4926.3	48.7
Aldoc	5902.4	2015.2	2404.7	2141.3	2317.1	2149.9	231.1
Gfap	5173.0	6064.0	1935.1	5423.3	3361.7	4564.8	3.7
Atp1b2	4396.0	1999.7	732.4	973.4	635.5	1384.2	107.5
Ndrg2	3894.9	1879.8	2244.4	1456.8	2014.3	2485.4	26.8
Cst3	3182.3	2648.9	3417.4	3068.8	4205.8	4473.9	153.5
Clu	2346.4	2862.8	726.7	1772.0	1108.5	1187.5	8.8
Atp1a2	2315.2	872.8	174.6	35.0	174.1	260.9	5.0
Sparc	1729.4	1238.8	1088.8	1107.8	970.7	1301.0	314.1
S100b	1544.7	1495.3	71.7	43.5	144.1	64.6	9.0
Agt	1031.7	747.5	1140.8	587.9	1495.3	1077.7	11.8
S100a1	888.4	1382.5	5.8	9.3	9.8	6.3	1.2
Slc1a2	851.2	271.8	14.9	5.1	9.7	33.3	3.9
Gja1	581.2	213.4	267.7	114.3	212.9	339.9	21.6
Aldh1a1	545.8	362.9	18.0	14.6	31.8	71.7	0.7
Aqp4	535.1	483.0	13.0	24.5	14.1	28.6	0.3
Pla2g7	530.6	306.6	427.1	107.3	658.2	563.2	1.1
Gjb6	291.8	80.5	0.9	0.4	0.7	1.9	0.4
Slc7a10	247.5	45.3	0.4	0.4	0.4	0.3	0.6
Id3	229.3	508.4	131.1	169.1	219.7	248.8	94.0
Slc4a4	216.0	80.9	28.7	4.9	17.1	33.0	2.1
Fam107a	212.7	57.6	0.2	0.1	0.2	0.7	0.2
Mdk	189.2	140.0	0.3	0.2	0.3	0.1	0.1
Myoc	180.5	39.4	0.5	0.7	2.3	1.0	0.2
Slc1a3	161.2	67.3	121.7	85.8	82.6	193.1	160.8
Etnppl	65.4	23.8	0.0	0.0	0.0	0.1	0.1
Luzp2	59.8	24.7	0.1	0.0	0.1	0.1	0.0
Slc2a4	25.8	22.1	1.2	0.5	1.7	3.6	0.2

Supplementary Table 2 | Comparison of gene expression levels (average FPKM) for selected astrocyte genes in: (i) astrocytes from healthy mouse spinal cord (SC) *in vivo*, (ii) astrocytes after SCI *in vivo*, (iii) cells derived from NPC after spontaneous cell autonomous regulated differentiation *in vitro*, or (iv) cells derived from NPC treated with CNTF, 1% FBS, or IGF-1 to promote differentiation *in vitro*.

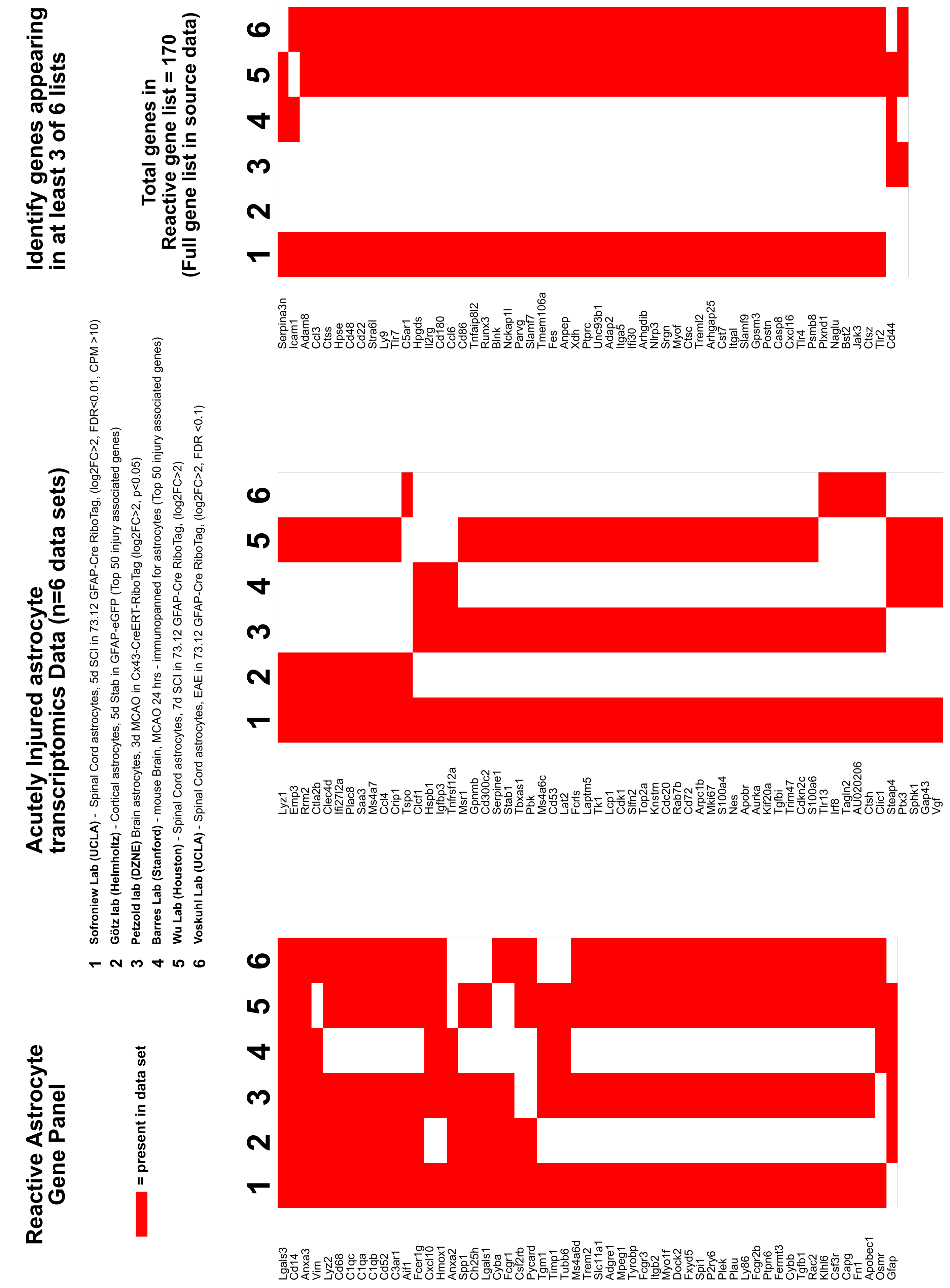

Supplementary Fig. 4 | Reactivity gene panel. Workflow for generating reactivity gene panel in astrocytes and list of reactivity gene panel (also available in source data).

a

EMT Gene Panel

EMT gene panel			
Abi3Bp	Dst	Lama1	Ptx3
Acta2	Ecm1	Lama2	Pvr
Adam12	Ecm2	Lama3	Qsox1
Anpep	Edil3	Lamc1	Rgs4
Aplp1	Efemp2	Lamc2	Rhob
Areg	Eln	Lgals1	Sat1
Basp1	Emp3	Lox	Scg2
Bdnf	Eno2	Loxl1	Sdc1
Bgn	Fap	Loxl2	Sdc4
Bmp1	Fas	Lrp1	Serpine1
Cadm1	Fbln1	Lrrc15	Serpine2
Cald1	Fbln2	Lum	Serpinh1
Calu	Fbln5	Magee1	Sfrp1
Cap2	Fbn1	Matn2	Sfrp4
Capg	Fbn2	Matn3	Sgcb
Ccn1	Fermt2	Mcm7	Sgcd
Ccn2	Fgf2	Mest	Sgcg
Cd44	Flna	Mfap5	Slc6A8
Cd59A	Fmod	Mgp	Slit2
Cdh11	Fn1	Mmp13	Slit3
Cdh2	Foxc2	Mmp14	Snai2
Cdh6	Fstl1	Mmp2	Sntb1
Col11A1	Fstl3	Mmp3	Sparc
Col12A1	Fuca1	Msx1	Spock1
Col16A1	Fzd8	Myl9	Spp1
Col1A1	Gadd45A	Mylk	Tagln
Col1A2	Gadd45B	Nid2	Tfpi2
Col3A1	Gas1	Nnmt	Tgfb1
Col4A1	Gem	Notch2	Tgfbi
Col4A2	Gja1	Nt5E	Tgfbr3
Col5A1	Glipr1	Ntm	Tgm2
Col5A2	Gpc1	Oxtr	Thbs1
Col5A3	Gpx7	P3H1	Thbs2
Col6A2	Grem1	Pcolce	Thy1
Col6A3	Htra1	Pcolce2	Timp1
Col7A1	Id2	Pdgfrb	Timp3
Col8A2	Igfbp2	Pdlim4	Tnc
Colgalt1	Igfbp3	Pfn2	Tnfaip3
Comp	Igfbp4	Plaur	Tnfrsf11B
Copa	Il15	Plod1	Tnfrsf12A
Crlf1	Il6	Plod2	Tpm1
Cthrc1	Inhba	Plod3	Tpm2
Cxcl1	Itga2	Pmepa1	Tpm4
Cxcl12	Itga5	Pmp22	Vcam1
Cxcl5	Itgav	Postn	Vcan
Dab2	Itgb1	Ppib	Vegfa
Dcn	Itgb3	Prrx1	Vegfc
Dkk1	Itgb5	Prss2	Vim
Dpysl3	Jun	Pthlh	Wipf1
			Wnt5A

Derived from EMT Gene Set Enrichment Analysis (GSEA) dataset from Molecular Signatures Database (Msigdb) with human specific genes removed

b

Overlap of Astrocyte State Gene Panels

Astrocytes & Reactivity	Reactivity & EMT	Astrocytes & EMT	In all 3 lists
C4b	Anpep	Ccn1	Vim
Gfap	Capg	Gja1	
	Cd44	Htra1	
	Emp3	Mmp14	
	Fn1	Notch2	
	Igfbp3	Pdlim4	
	Itga5	Sdc4	
	Lgals1	Serpine2	
	Postn	Sparc	
	Ptx3	Timp3	
	Serpine1	Tnc	
	Spp1	Vcam1	
	Tgfb1		
	Tgfbi		
	Timp1		
	Tnfrsf12a		

Supplementary Table 3 | EMT gene panel and overlapping genes between the three astrocyte state gene panels. a. EMT gene panel. **b.** Overlap of the astrocyte state gene panels.

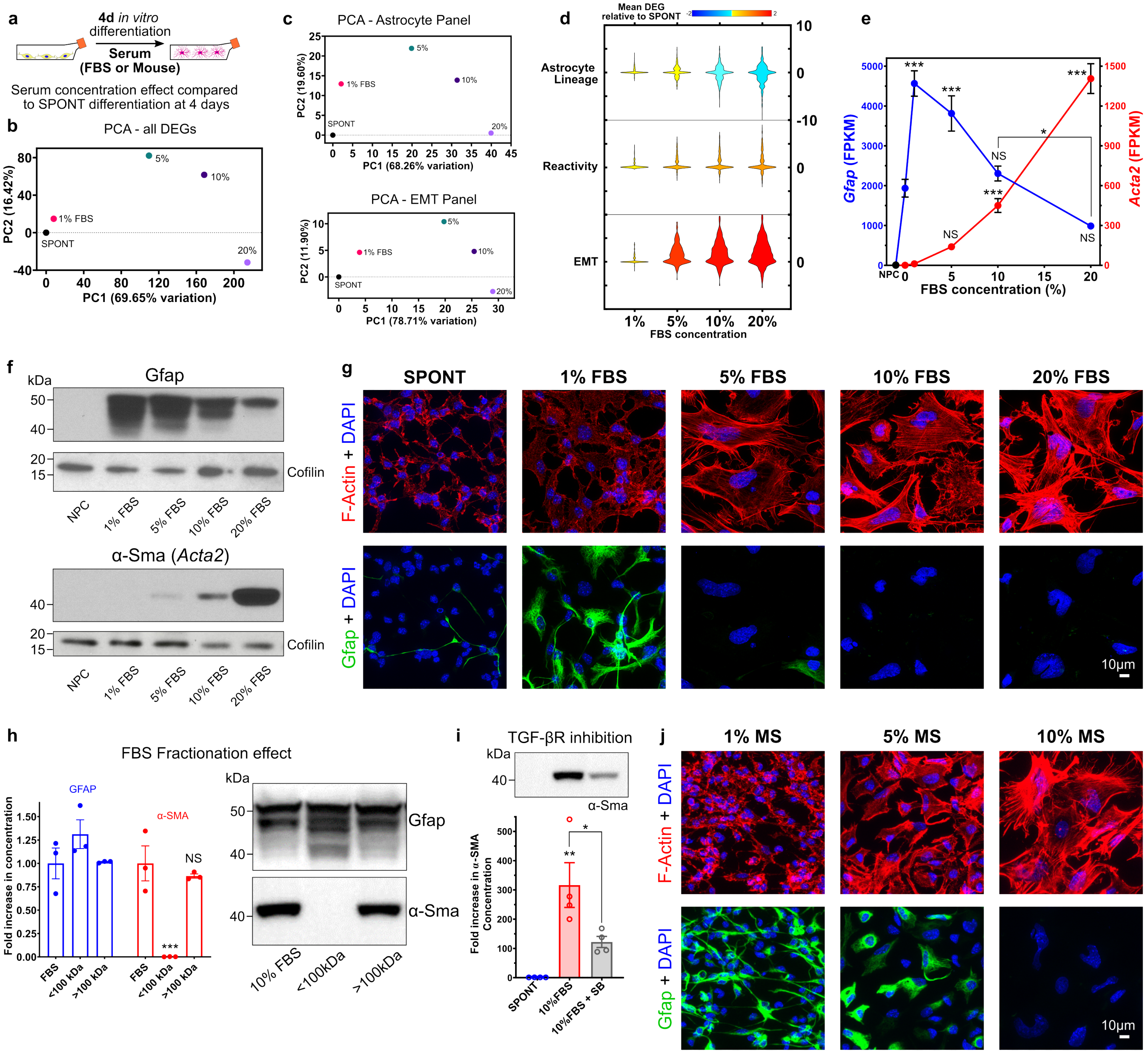

Supplementary Fig. 5 | Serum exposure induces concentration dependent myofibroblast-like phenotypes in NPC *in vitro*. a Schematic summarizing experimental approach for evaluating serum (Fetal Bovine Serum (FBS) and Mouse Serum (MS)) exposure effects on NPC differentiation *in vitro* by, ICC, WB and Bulk RiboTag. **b** PCA of all DEGs associated with exposure to increasing concentrations of FBS indicating a strong FBS concentration dependent divergence away from SPONT differentiation. **c** PCA of DEGs from healthy Astrocyte and EMT gene panels for FBS treated NPC showing comparable FBS concentration dependent divergence away from SPONT differentiation as observed in b). **d** Violin plot of DEGs for astrocyte state gene panels for FBS treated NPC showing a prominent FBS dependent increase in EMT gene expression and a concurrent decrease in healthy astrocyte gene expression. **e** FBS concentration dependent changes in Gfap and Acta2 gene expression measured in FPKM. N=4 per group *** p-value < 0.0001, * p-value < 0.02, NS = Not significant. One way ANOVA with Tukey multiple comparison test for Gfap and Acta2 run independently, comparisons made with SPONT unless indicated. **f** Western blot (WB) for Gfap and α-Sma showing FBS concentration dependent expression changes. Gfap protein expression decreases with higher FBS concentrations and is associated with loss of lower molecular weight isoform bands. α-Sma protein expression increases with FBS concentration exponentially. Cofilin used as WB and protein concentration reference. **g** ICC staining of FBS treated NPC for Phalloidin (F-Actin), which shows myofibroblast-like phenotype, and Gfap. ICC staining shows similar FBS concentration dependent effects as in e) and f). **h** Quantification of WB for Gfap and α-Sma for FBS and fractionated FBS treatment showing higher molecular weight species (>100 kDa) in FBS stimulated α-Sma expression. N=3 per group *** p-value = 0.0002, NS = Not significant. One way ANOVA with Tukey multiple comparison. **i** Quantification of WB for FBS induced α-Sma expression following TGF-βR inhibition by SB431542 (SB). Comparisons made between SPONT and 10% FBS with and without concurrent TGF-βR inhibition by SB. N=4 per group, ** p-value = 0.0022, * p-value = 0.0351, NS = Not significant. One way ANOVA with Tukey multiple comparison. **j** ICC staining of Mouse serum (MS) treated NPC for Phalloidin (F-Actin) and Gfap showing a conserved serum dependent effect on myofibroblast-like phenotype with increased serum concentration exposure. All bar graphs are mean ± s.e.m. with individual datapoints overlayed.

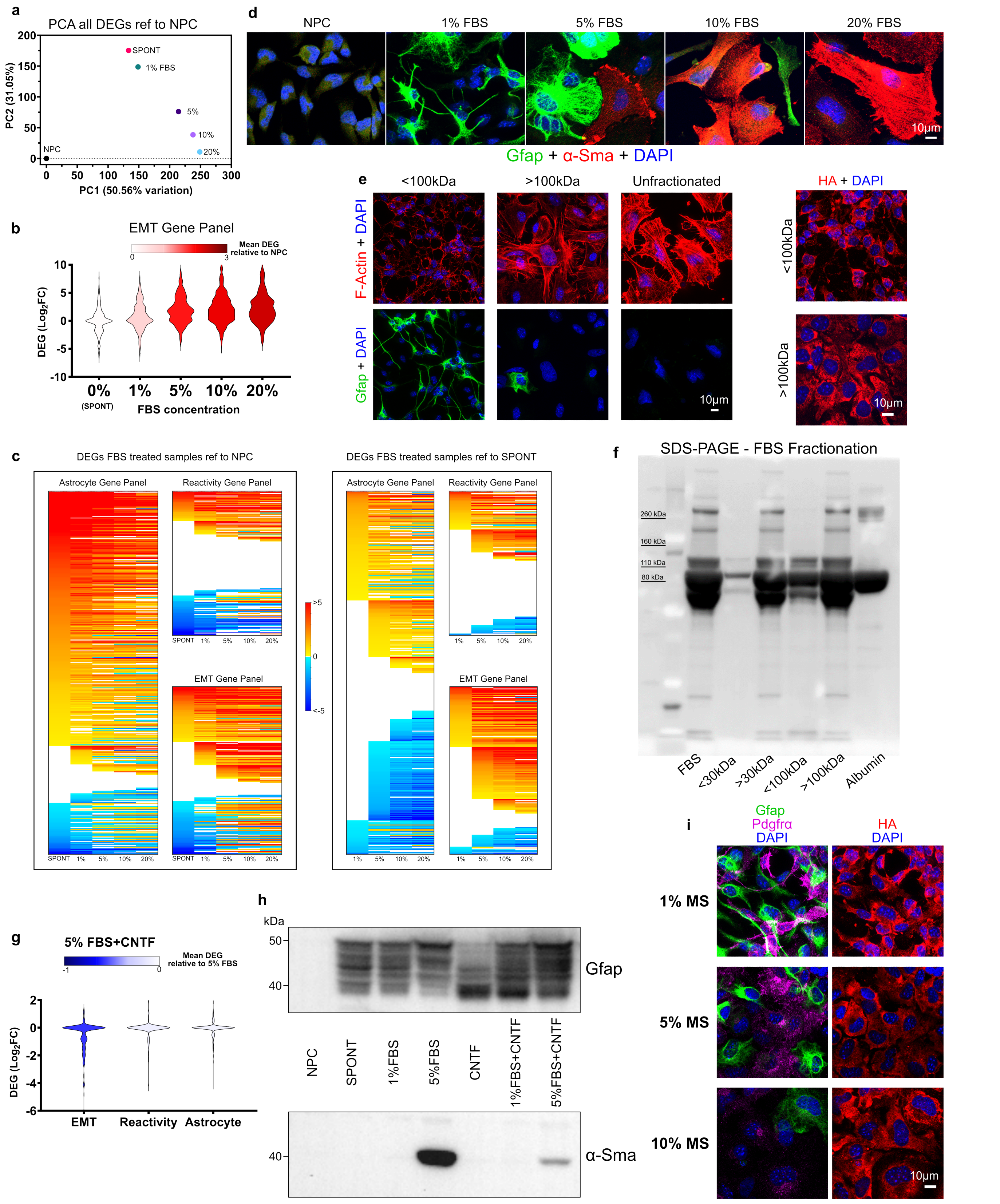

Supplementary Fig. 6 | Serum exposure evokes myofibroblast-like phenotypes in NPC *in vitro.* a PCA of all DEGs for SPONT and increasing concentrations of FBS referenced to NPC showing increased divergence from NPC with increased FBS concentration. **b** Violin Plot of DEGs for SPONT and increasing concentrations of FBS referenced to NPC for EMT gene panel genes showing a concentration dependent increase in expression of EMT gene panel genes. **c** Heatmaps displaying DEGs for increasing concentrations of FBS referenced to either NPC or SPONT separated into astrocyte, reactivity and EMT gene panels. **d** ICC staining of NPC exposed to increasing concentrations of FBS showing changes in Gfap and α-Sma expression as a function of FBS concentration. Cells counterstained with DAPI. **e** ICC staining of NPC exposed to FBS fractionated by molecular size cut-off centrifugal filters. NPC treated with the <100 kDa fraction, >100kDa fraction and unfractionated FBRS and stained with phalloidin to detect F-Actin expression and counter-stained with Gfap and DAPI. NPC treated with fractionated FBS samples also stained with HA and DAPI. **f** SDS-PAGE of FBS and fractionated samples stained with SYPRO Ruby Protein Gel Stain showing the effectiveness of molecular size cut-off centrifugal

filters (30kDa and 100kDa) to separate out higher molecular weight constituents. Unfractionated FBS and albumin used as controls. **g** Violin Plot of DEGs for NPC treated with 5%FBS and CNTF referenced to 5%FBS alone showing the effect of CNTF on attenuating expression of EMT genes. **h** Western blot of FBS and CNTF treated samples stained for Gfap and α-Sma showing CNTF and FBS dependent effects on expression of Gfap Isoforms and α-Sma. **i** ICC staining of NPC exposed to increasing concentration of mouse serum (MS) showing decreased expression of Gfap and Pdgfr-α and altered cell size and morphology indicated by HA staining. Note that the heightened myofibroblast phenotypes caused by increasing concentrations of FBS involved elevated expression of canonical EMT transcription factors (*Snai1*, *Snai2, Twist1*), myofibroblast cytoskeletal proteins (*Acta2, Tagln*), profibrotic growth factors (*Ccn2* (Ctgf), *Tgfbi* (Tgf-β), *Il6*) and fibrotic extracellular matrix proteins (*Fap, Col1a1, Fn1, Thbs1*). The FBS concentration dependent downregulation in astrocyte genes was most apparent for genes associated with healthy astrocyte functions such as molecular transport and included *Slc6a11, Atp1a2, Rlbp1, Slc6a1, Kcnj1, Agt* and *Pla2g7*. Canonical astrocyte gene *Gfap* also showed a strong serum concentration dependent decrease in expression that directly opposed the trend in expression for canonical EMT associated gene *Acta2* (smooth muscle actin (α-Sma)) which increased exponentially.

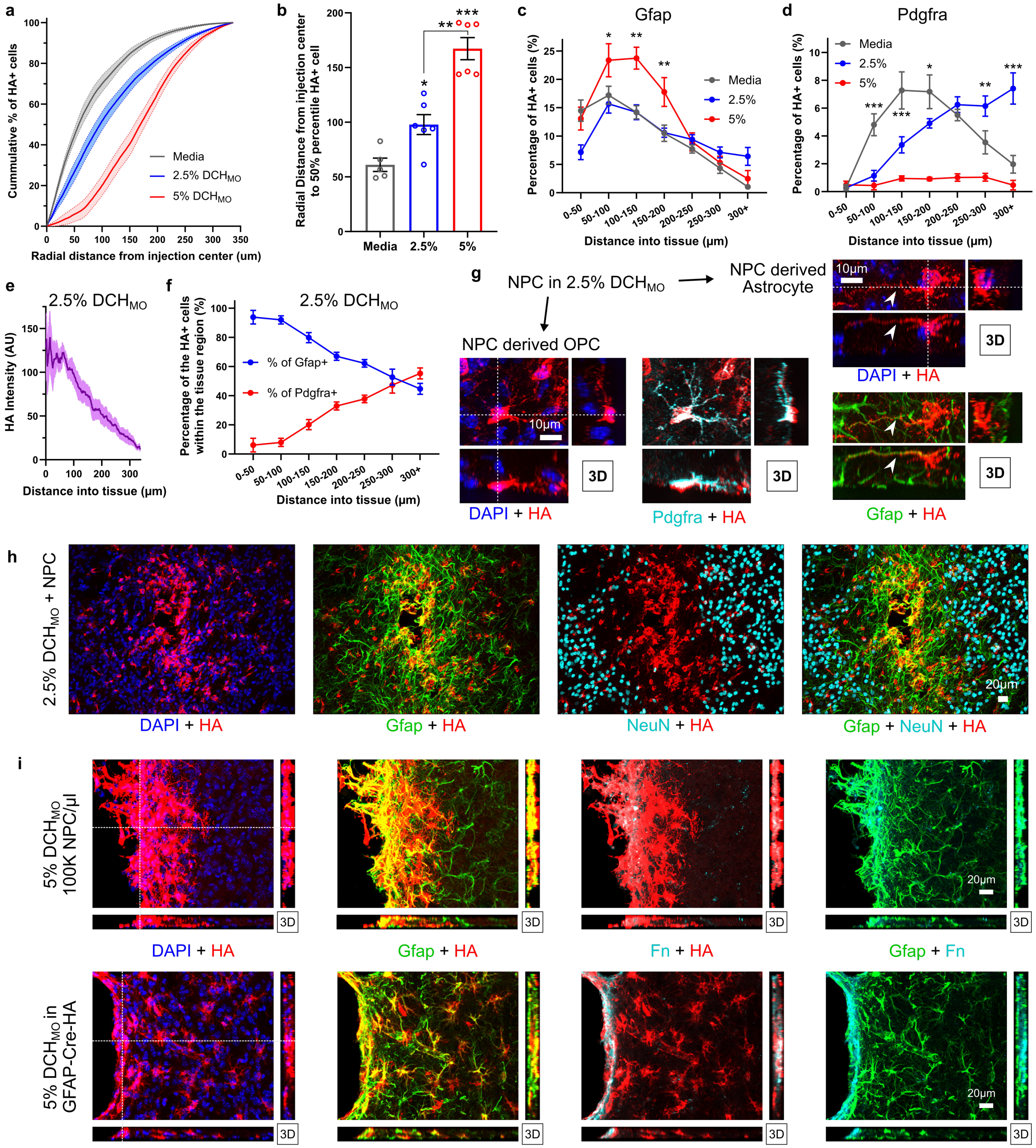

Supplementary Fig. 7 | NPC grafted into healthy mouse striatum differentiate into astrocyte and oligodendroglia lineage cells in a spatial and carrier dependent manner. a Quantification of the cumulative spatial distribution of HA+ cells, referenced from the center of injection, for media and DCH_{MO} hydrogel carrier samples. For each sample the darkened line represents the mean and shaded area indicates standard error of mean (SEM) (N=6 for both hydrogels, N=5 for Media). **b** Quantification of the Radial distance to the 50th percentile HA+ cell, referenced from the center of injection, for media and DCH_{MO} hydrogel carrier samples. (Mean + SEM) (N=6 for both hydrogels, N=5 for Media). *** p-value < 0.0001, ** p-value < 0.0002, * p-value < 0.032, One way ANOVA with Tukey multiple comparison test. **c** Percentage distribution of HA-positive and Gfap-positive cells within define spatial zones, referenced from the center of injection, for media and DCH_{MO} hydrogel carrier samples. (Mean + SEM) (N=6 for both hydrogels, N=5 for Media). *** p-value < 0.0001, ** p-value < 0.005, * p-value < 0.03, One way ANOVA with Tukey multiple comparison test. **d** Percentage distribution of HA-positive and Pdgfrα-postive cells within define spatial zones, referenced from the center of injection, for media and DCH_{MO} hydrogel carrier samples. (Mean + SEM) (N=6 for both hydrogels, N=5 for Media). *** p-value < 0.0001, ** p-value < 0.005, * p-value < 0.03, One way ANOVA with Tukey multiple comparison test. **e** IHC Intensity trace of HA positive grafted cells from 2.5% DCH_{MO} carrier group showing localization of graft within a 350 μm radius of injection center. **f** Distribution of the Percentage of total HA-positive cells that reside within specified spatial zones that are Pdgfra positive and Gfap positive. The proportion of Pdgfra-positive grafted cells increases radially from the injection center and is the predominant differentiated cell type in radial zones greater than 300 μm. **g** High magnification 3D images showing the morphology and Pdgfra-positive and Gfap positive cells that adopt individual domains in neural tissue. **h** Survey Immunohistochemistry (IHC) images of grafted HA-positive NPC in healthy striatum stained for astrocytes (Gfap) and

neurons (NeuN) at 2 weeks after NPC transplantation. HA-positive grafted NPC do not express NeuN indicating they do not differentiate into Neurons in healthy striatum at 2 weeks. **i** High magnification 3D view showing HA-positive grafted NPC in 5% DCH_{MO} hydrogel carrier showing formation of Gfap-positive borders by grafted NPC that express Fibronectin (FN). Host astrocyte borders are formed around 5% DCH_{MO} hydrogel carrier that are morphologically similar to the borders formed by grafted NPC and also expressed Fibronectin at the border interface. All bar graphs are mean ± s.e.m. with individual datapoints overlayed. AU = arbitrary units.

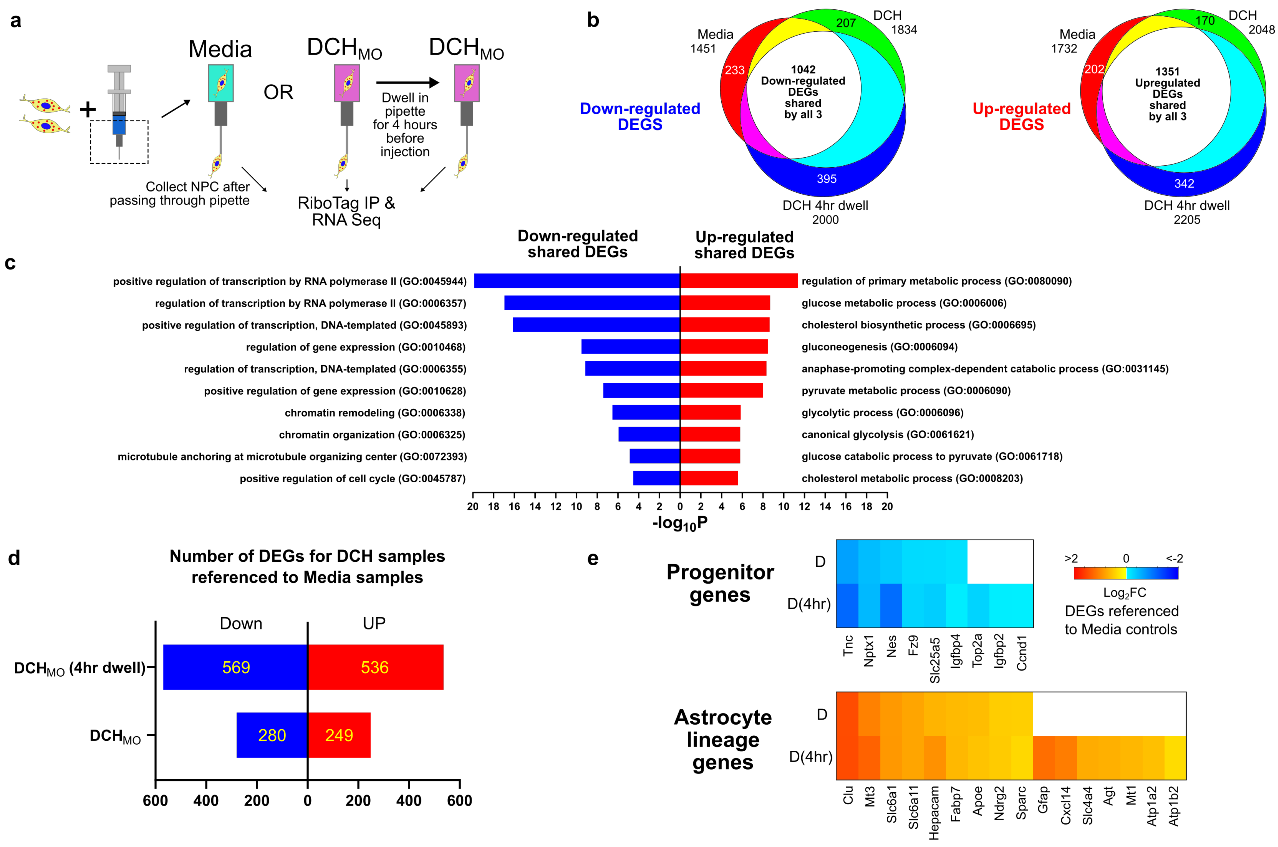

Supplementary Fig. 8 | NPC injected through glass pipettes are minimally altered by the carrier vehicle used. a Schematic detailing the experimental paradigm to test the effect of injection through pulled glass pipettes in media or hydrogel carrier on NPC phenotype. In one group, NPC loaded into hydrogel carriers were allowed to dwell in the pipette for 4 hours before being passed through the pipette to evaluate changes to NPC phenotype taking place over a typical transplantation session. **b** Ven diagram of Upregulated and Downregulated DEGs for NPC passed through pipettes in media or hydrogel or allowed to dwell in hydrogel for 4 hours showing high overlap of shared DEGs across the three conditions. **c** Gene Ontology (GO) term analysis of the shared Up-regulate and Down-regulated DEGs. Shared down-regulated DEGs indicate an overall decrease in transcription and cell cycle processes as a result of injection through pipettes. Shared Up-regulated DEGs suggests increased metabolic processes in NPC upon injection through pulled glass pipettes. **d** Total number of hydrogel specific upregulated and downregulated DEGs when referenced to NPC in media samples (FDR <0.01). The 4-hour dwell in the pipette effectively doubles the number of DEGs. **e** Heat map of Astrocyte and Progenitor genes altered in hydrogel samples referenced to NPC in media samples. NPC in hydrogel carriers show an increase in astrocyte gene expression and a decrease in progenitor gene expression with the 4-hour dwell enhancing both effects. Note that there were 1351 shared upregulated genes that were consistent with increased metabolic processes as assessed by Gene Ontology term enrichment, whereas 1042 shared downregulated genes suggested a reduction in cell cycle and transcription processes. Comparisons of hydrogel and media carrier samples identified a modest increase in astrocyte differentiation signature in the hydrogel group with upregulation of astrocyte lineage genes *Clu, Mt3, Slc6a1, Hepacam* among others and a concurrent downregulation of some progenitor genes such as *Igfbp4, Tnc, Nes* and *Slc25a5*. Persistent retention of cells in the pipette for 4 hours further increased the expression of these astrocyte genes, increased expression of other astrocyte genes (including Gfap), and caused further downregulation of progenitor genes which now included *Top2a, Igfbp2* and *Ccnd1*, suggesting that the cells may undergo some differentiation over the course of a standard injection session and that astrocyte phenotypes emerge more quickly than other definable phenotypes. However, despite these apparent DEGs caused by dwell time differences in the pipette, we observed no obvious trend among animals that received transplants first or last in an injection session suggesting these small variations in DEGs may not significantly impact overall graft outcomes *in vivo*.

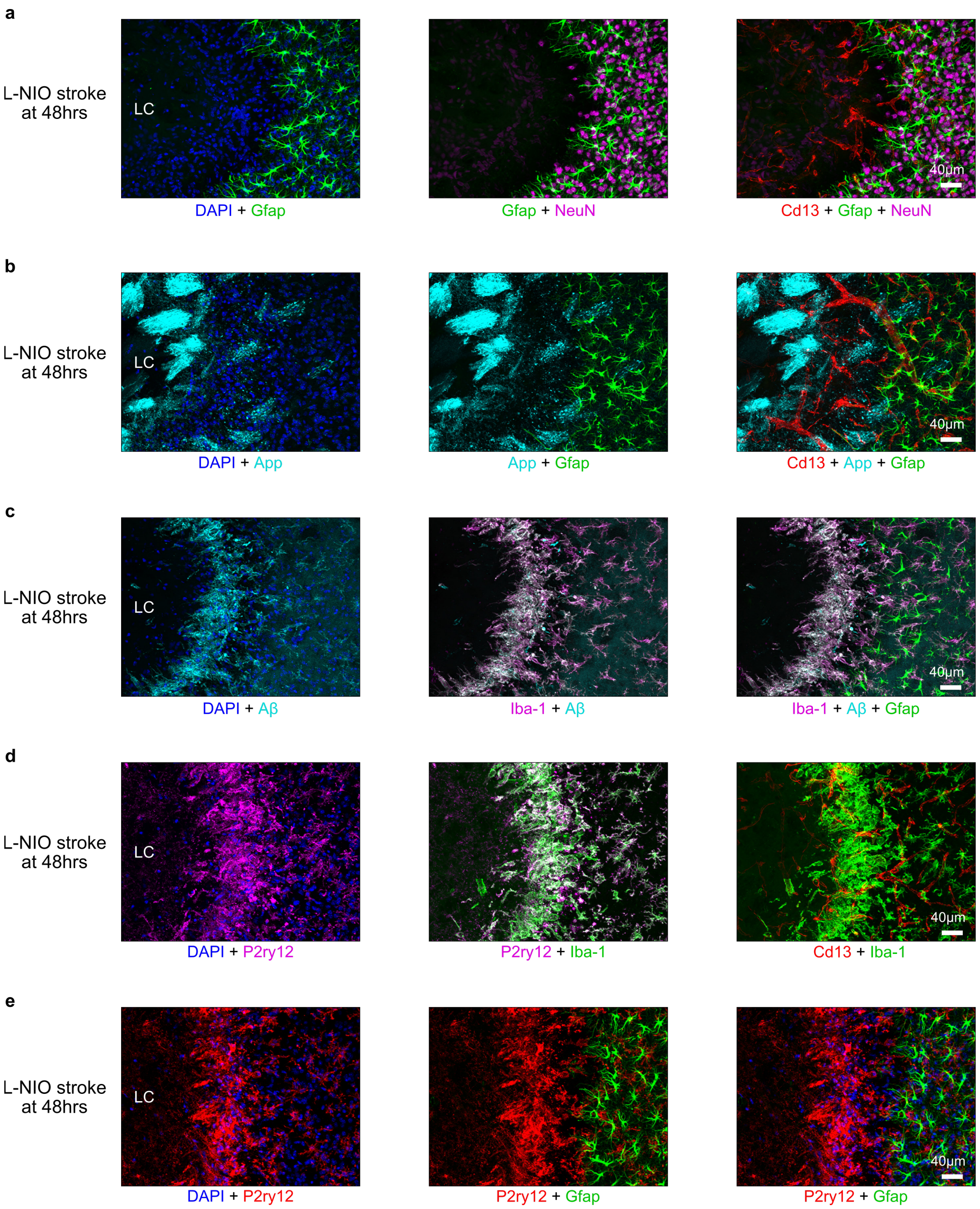

Supplementary Fig. 9 | Survey IHC images of striatal stroke lesion cores (LC) at 48 hours after injury induced by L-NIO injection. a Stroke lesion cores (LC) are defined by infarcted tissue that shows complete loss of neurons (NeuN) and astrocytes. **b** Stroke LC show accumulation of axon damage (App) that is isolated to the LC at 48 hours. **c & d** Accumulated amyloid precursor protein (App) is enzymatically converted to Aβ fragments that colocalize with Iba-1- and P2ry12- positive microglia and demarcates the border of the forming lesion. **e** At 48 hours after stroke injury the microglia front (P2yr12-positive) of the border precedes the zone of viable astrocytes that have only just initiated the process to alter their phenotype necessary to reorganize and form the astrocyte border.

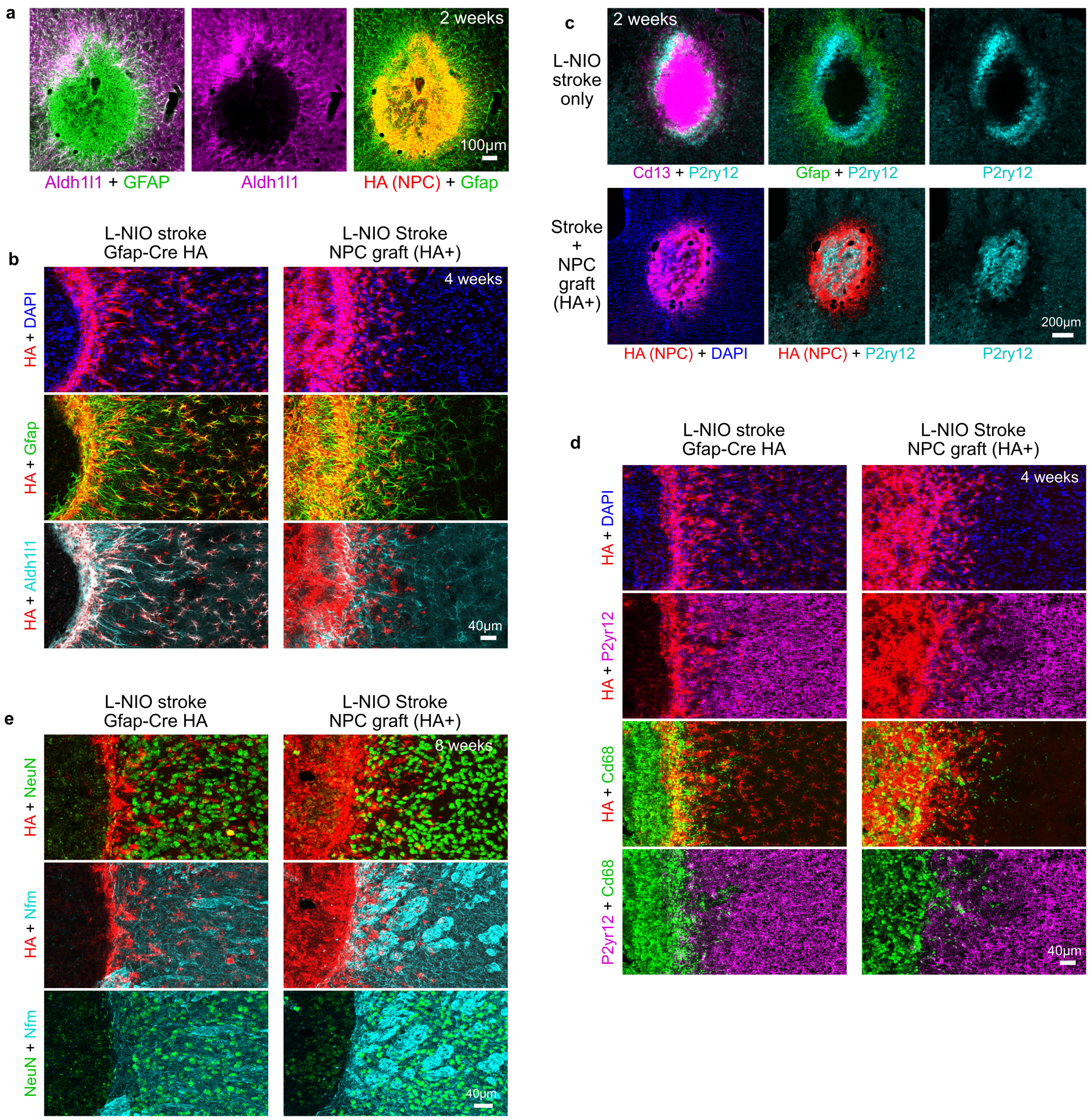

Supplementary Fig. 10 | NPC grafted into striatal stroke generate astroglia that reduce fibrotic scar and bridge lesions but do not repopulate lesions with neural tissue. a Survey IHC images showing that NPC differentiate into Gfap-positive astroglia upon grafting into stroke lesions but do not express mature astrocyte markers such as Aldh1l1. **c** Detailed lesion interface IHC images of L-NIO stroke lesions at 4 weeks after stroke in 73.12 Gfap-Cre HA mice. HA-positive host astrocytes that express Gfap and Aldh1l1 form discrete borders with non-neural lesion cores. NPC grafting into L-NIO strokes comprehensively fill the lesion with Gfap-positive cells, but only grafted cells located at the host neural tissue interface begin to express Aldh1l1 at 4 weeks. **d** Survey IHC image showing NPC treated stroke lesions transiently containing P2yr12-positive CNS derived microglia at 2 weeks whereas untreated stroke lesions do not contain P2yr12-positive microglia in the lesion core with Cd13-positive macrophages and fibroblasts instead predominating in this lesion compartment. **e** Detailed IHC images showing an NPC graft induced change in Cd68-positive immune cells within stroke lesion cores at 4 weeks compared with untreated strokes made in Gfap-Cre HA mice that have HA-positive host astrocytes. P2yr12-positive microglia are no longer detected within the graft. **f** Detailed IHC images showing that NPC grafting into stroke lesions does not provoke neuronal repopulation or striatal axon regrowth into lesion cores by 8 weeks.

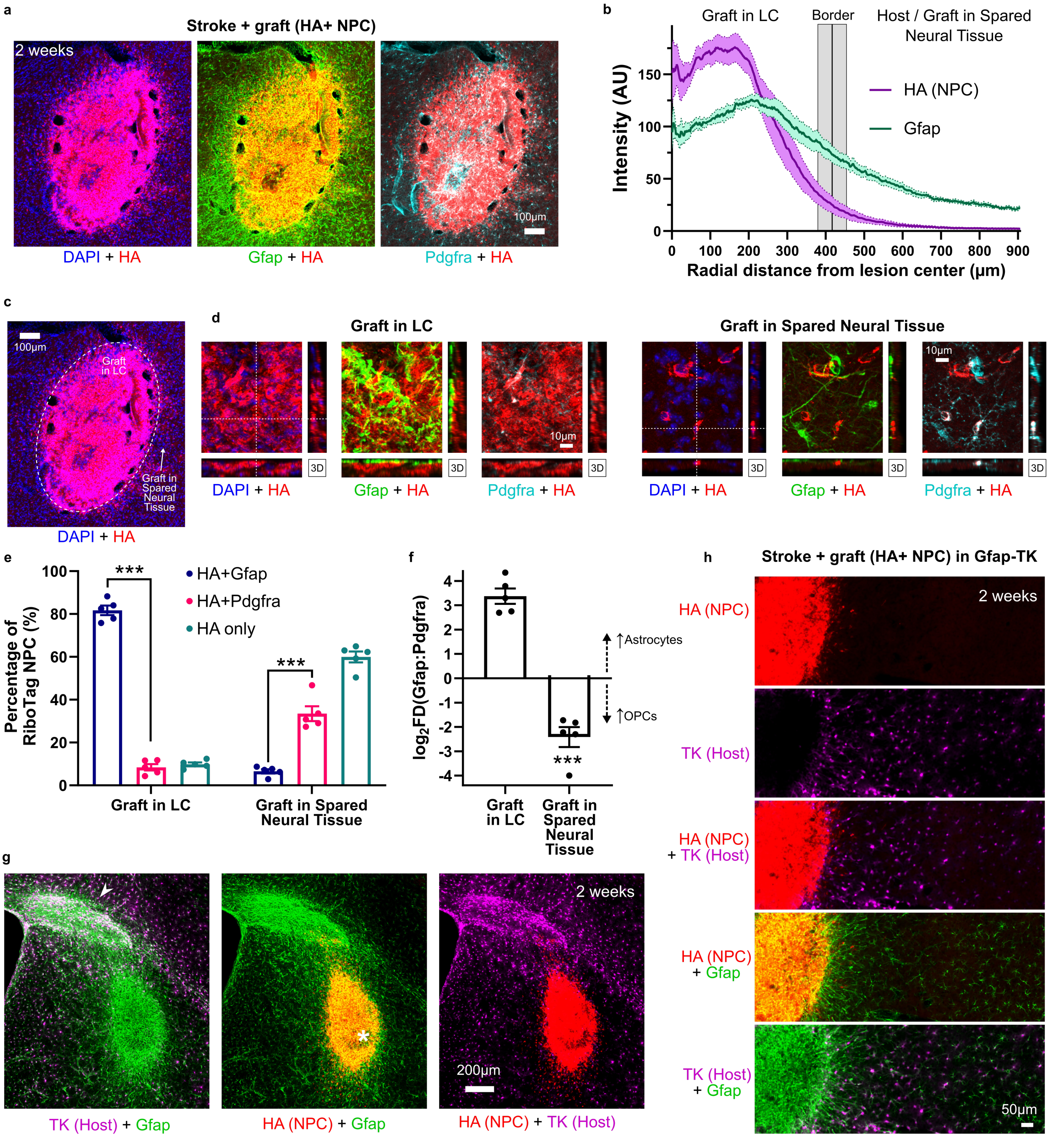

Supplementary Fig. 11 | Distribution of NPC phenotypes upon grafting into striatal stroke. a Survey image of NPC grafts in stroke at 2 weeks. NPC grafted into strokes remain local at lesions and differentiate into glia that express either the astroglia marker Gfap or OPC marker Pdgfra. **b** Intensity trace of HA and Gfap for NPC grafts in stroke as mean ± s.e.m. (s.e.m represented as shaded region) (N=5). Grafted NPC remain local within lesion cores with 95% of grafted cells persisting within an average radius of approximately 400 μm from the center of the lesion. **c.** Survey image of NPC grafts in stroke at 2 weeks showing that grafted cells can be separated into two distinct phenotypes depending on whether they reside: (i) within the LC (~95% of total grafted cells) or (ii) in spared neural tissue (~5% of grafted cells). **d** Detailed higher magnification 3D imaging showing that NPC grafts in LC are dense overlapping and express Gfap whereas grafted cells residing in preserved neural tissue adopt individual domains and largely express Pdgfra. **e**. Quantification of percentage of grafted cells expressing Gfap or Pdgfra or neither marker in LC or in spared neural tissue. (Mean + SEM) (N=5). *** p-value < 0.0001, Two-way ANOVA with Tukey multiple comparison test. **f.** Log$_2$FC differences in Gfap/Pdgfra expression ratio for grafted cells in LC and spared neural tissue. Grafts show proportionally higher expression of astrocyte marker Gfap in lesion core whereas grafts residing in preserved neural tissue express Pdgfra in great abundance. (Mean ± s.e.m.) (N=5). *** p-value < 0.0001, Students t-test, two-sided. **g** Survey IHC images of NPC grafted stroke lesions at 2 weeks in GFAP-TK mice showing NPC

graft contribution to stroke lesion remodeling (*). Host astrocytes have minimal contribution to stroke lesion remodeling but have responded to tissue disruption from pipette insertion (➤) located away from the lesion. **h** detailed IHC images showing that NPC grafting into stroke lesions localize to lesions and assume the astrocyte border forming roles in lieu of host astrocytes (TK-positive cells) which instead persist in preserved neural tissue. All bar graphs are mean ± s.e.m. with individual datapoints overlayed.

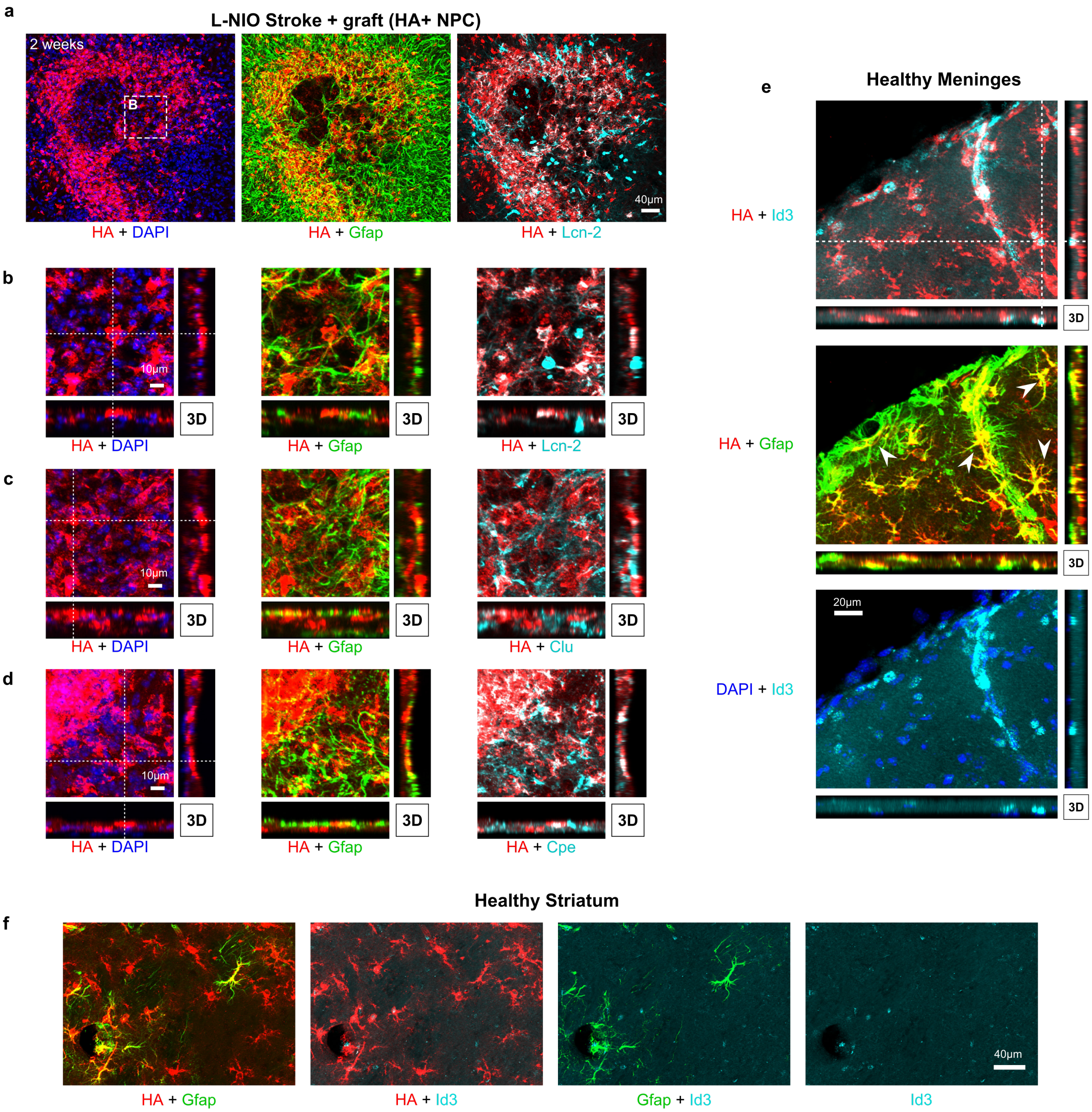

Supplementary Fig. 12 | Astroglia derived from NPC grafted into CNS lesions become border-forming astroglia. a Survey IHC imaging of NPC grafted into L-NIO stroke lesions showing Gfap-positive, HA-positive grafted cells expressing the astrocyte reactivity marker Lipocalin-2 (Lcn-2) in cells forming borders around lesion cores. **b** Detailed, higher magnification 3D imaging showing co-localization of Lcn-2 in Gfap-positive, HA-positive grafted cells in stroke. **c** Detailed, higher magnification 3D imaging showing co-localization of astrocyte reactivity marker Clusterin (apolipoprotein J) (Clu) in Gfap-positive, HA-positive grafted cells in stroke. **d** Detailed, higher magnification 3D imaging showing co-localization of astrocyte marker Carboxypeptidase E (Cpe) in Gfap-positive, HA-positive grafted cells in stroke. **e** Detailed, higher magnification 3D imaging showing co-localization of Id3 in HA-positive host astrocytes. Several Id-3, HA and Gfap colocalized host astrocytes are indicated by overlayed arrows. **f** Survey IHC imaging of healthy mouse striatum showing relatively low expression of Id3 in protoplasmic astrocytes in the striatum. Astrocytes that form the glia limitans around blood vessels in the striatum show higher Id3 expression.

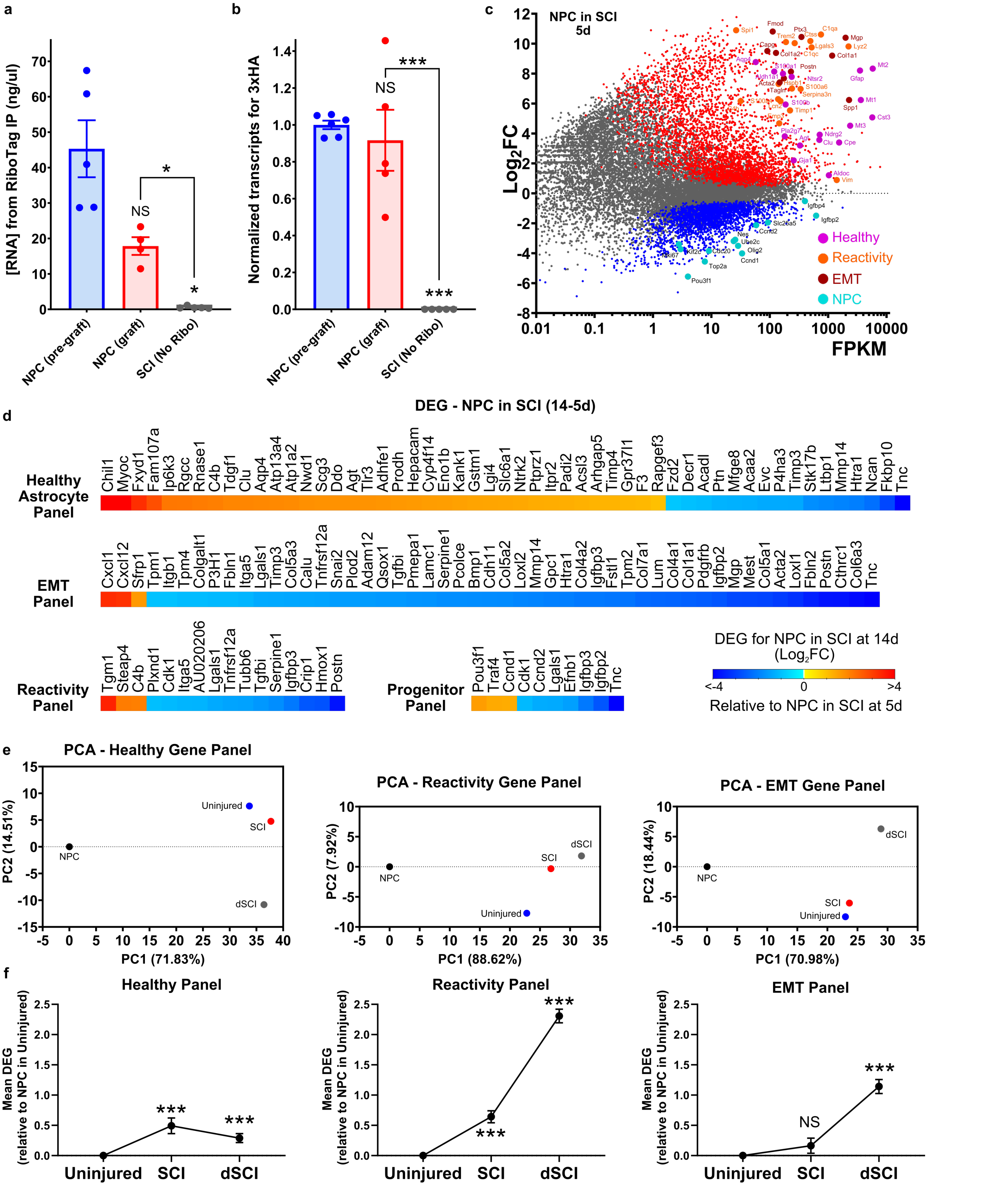

Supplementary Fig. 13 | RiboTag transcriptomics on NPC transplanted into subacute crush spinal cord injury reveals an astroglial border phenotype that evolves over time and is altered by lesion severity. a Concentration of RNA recovered following RiboTag Immunoprecipitation and mRNA purification from NPC before grafting and 5 days after grafting into a 2-day crush SCI lesion. RNA recovered from NPC after grafting is not significantly reduced compared to pre-graft levels. There is minimal recovery of RNA from SCI lesions that did not receive a graft of HA-positive cells (SCI (no RiboTag)) suggesting high specificity towards recovery of grafted cell RNA using the RiboTag methods. N=5 for NPC (pre-graft) and SCI (No Ribo), N=4 for NPC (graft), * p-value < 0.02, NS = not significant, Welch ANOVA assuming non-equal standard deviation with Dunnett's T3 multiple comparison. **b** Normalized counts of 3xHA transcripts from RNA-Seq data sets for pre-graft NPC and NPC recovered at 5 days after grafting into a 2-day crush SCI lesion showing no statistical differences in the frequency of detection of 3xHA transcripts in samples recovered from the graft compared to the pre-graft sample that contains 100% HA-positive cells. No 3xHA transcripts were detected in samples recovered from SCI tissues that did not

receive a graft of HA-positive cells. N=5 for all groups, *** p-value < 0.0001, NS = not significant, One way ANOVA with Tukey multiple comparison test. **c** MA plot of FPKM levels versus DEGs for NPC grafted into 2-day crush SCI lesions (N=5 mice). Significant Up (red) and Down (Blue) DEGs have FDR <0.01. Select DEGs for healthy astrocytes, reactivity, EMT and progenitor gene panel genes are indicated. **d** Heatmaps of all healthy astrocyte, reactivity, EMT and NPC/progenitor gene panel DEGs detected in NPC grafts recovered at 14 days after grafting when compared with NPC grafts recovered at 5 days after grafting into 2-day SCI lesions (N=5 for both time points). DEGs have FDR <0.01. NPC recovered at 14 days show overall net increase in expression of healthy astrocyte genes and a decrease in expression of EMT genes compared with the grafts recovered at 5 days. (N=5 mice per group). **e** PCA of healthy astrocyte, reactivity and EMT gene panels for NPC grafted into different tissue environments (uninjured, SCI and double crush SCI (dSCI)). (N=5 mice per group). **f** Plots of Mean DEGs for healthy astrocyte, reactivity and EMT gene panels for NPC grafts recovered from SCI and dSCI referenced to NPC grafts recovered from uninjured spinal cord tissue. (N=5 mice per group) *** p-value < 0.0001, NS = not significant, One way ANOVA with Tukey multiple comparison test. All bar graphs are mean ± s.e.m. with individual datapoints overlayed.

Healthy Genes

Gene	NPC		Astrocytes	
Mlc1	110.1<br>498.5	↑	728.9<br>483.5	↓
Atp1b2	25.9<br>1361.0	↑	4396.0<br>1999.7	↓
Bcan	144.8<br>184.4	↑	464.3<br>186.6	↓
Sparcl1	100.8<br>717.1	↑	4116.2<br>1128.2	↓
Itih3	0.1<br>49.2	↑	282.6<br>52.8	↓
Gpr37l1	1.8<br>573.3	↑	878.3<br>629.0	↓
Cpe	69.1<br>2732.7	↑	2548.7<br>1590.9	↓
Timp4	16.2<br>171.7	↑	279.4<br>178.8	↓
Ntsr2	0.7<br>410.8	↑	885.8<br>496.5	↓
Sparc	564.4<br>1066.1	↑	1729.4<br>1238.8	↓
Ndrg2	11.8<br>715.3	↑	3894.9<br>1879.8	↓
Aldoc	386.1<br>771.2	↑	5902.4<br>2015.2	↓
Atp1a2	2.4<br>398.0	↑	2315.2<br>872.8	↓
Lrig1	38.8<br>83.5	↑	128.0<br>81.7	↓
Agt	11.0<br>994.7	↑	1031.7<br>747.5	↓
Sdc4	49.8<br>391.4	↑	606.2<br>442.1	↓
Cst3	181.5<br>5497.3	↑	3182.3<br>2648.9	↓
Gja1	54.2<br>324.4	↑	581.2<br>213.4	↓
Slc25a18	65.8<br>163.0	↑	383.0<br>185.4	↓
Eva1a	0.2<br>14.7	↑	87.8<br>16.1	↓
S1pr1	1.5<br>46.9	↑	178.5<br>55.3	↓
Slc6a11	1.8<br>55.8	↑	763.1<br>117.8	↓
Fam107a	0.0<br>44.3	↑	212.7<br>57.6	↓
Lix1	0.3<br>19.1	↑	27.3<br>18.8	↓
Hspa2	5.3<br>38.2	↑	42.2<br>38.4	↓
Hepacam	1.9<br>192.6	↑	381.7<br>249.4	↓
Acsl3	44.2<br>67.2	↑	283.6<br>78.1	↓
Id4	0.7<br>139.0	↑	200.3<br>110.0	↓
Pla2g7	0.9<br>187.4	↑	530.6<br>306.6	↓
Kcnj10	12.5<br>148.9	↑	797.1<br>347.2	↓
Gprc5b	29.6<br>87.2	↑	258.7<br>114.1	↓
Tspan7	66.1<br>128.8	↑	607.7<br>217.5	↓
Ttyh1	5.4<br>218.4	↑	1019.9<br>569.0	↓
Slc1a2	1.7<br>85.3	↑	851.2<br>271.8	↓
Ntrk2	2.0<br>115.8	↑	155.0<br>80.4	↓
Sfxn5	11.9<br>97.7	↑	246.0<br>138.5	↓
Smox	45.8<br>73.1	↑	146.5<br>81.0	↓
Mfsd2a	12.1<br>49.0	↑	115.9<br>35.5	↓
Mmd2	1.4<br>111.6	↑	381.2<br>207.3	↓
Cd81	547.8<br>897.6	↑	1781.1<br>1469.8	↓
Myoc	0.1<br>182.2	↑	180.5<br>39.4	↓
Pygb	19.1<br>89.7	↑	254.5<br>136.7	↓
2310022B05Rik	22.0<br>54.3	↑	203.1<br>78.5	↓
Slc6a1	13.7<br>42.3	↑	418.6<br>98.5	↓
Phkg1	0.7<br>27.0	↑	62.9<br>32.0	↓
Fabp7	1039.7<br>557.8	↓	336.6<br>624.6	↑
Id3	290.5<br>451.8	↑	229.3<br>508.4	↑
Clu	21.1<br>2449.5	↑	2346.4<br>2862.8	↑
Vim	600.4<br>1178.9	↑	163.3<br>1096.2	↑
Mt2	19.2<br>6815.3	↑	3879.4<br>7885.2	↑

Reactivity Genes

Gene	NPC		Astrocytes	
Tgm1	0.03<br>82.2	↑	0.1<br>35.9	↑
Ptx3	0.2<br>443.8	↑	2.4<br>72.7	↑
Cd52	0.0<br>35.6	↑	0.9<br>23.6	↑
Lyz2	0.6<br>383.8	↑	4.1<br>380.5	↑
Tyrobp	0.1<br>85.0	↑	1.7<br>77.4	↑
Fcer1g	0.1<br>128.0	↑	2.1<br>61.5	↑
C1qc	0.2<br>159.0	↑	3.5<br>132.0	↑
Laptm5	0.05<br>85.3	↑	1.3<br>24.5	↑
Fcrls	0.0<br>23.2	↑	0.4<br>14.5	↑
C1qa	0.2<br>193.0	↑	6.2<br>181.4	↑
Trem2	0.1<br>51.0	↑	0.8<br>43.6	↑
C1qb	0.03<br>26.6	↑	0.9<br>26.5	↑
Fcgr2b	0.1<br>115.3	↑	1.7<br>39.1	↑
Ctss	0.2<br>150.9	↑	5.3<br>127.7	↑
Steap4	0.2<br>38.2	↑	0.2<br>23.9	↑
Ly86	0.0<br>32.9	↑	1.4<br>19.3	↑
Mpeg1	0.1<br>42.4	↑	0.8<br>31.2	↑
Postn	0.1<br>14.9	↑	0.3<br>24.8	↑
Gpnmb	0.2<br>110.1	↑	3.7<br>91.0	↑
Fxyd5	0.1<br>37.1	↑	0.9<br>20.6	↑
Fcgr3	0.0<br>20.1	↑	0.7<br>13.2	↑
S100a4	0.9<br>394.4	↑	26.0<br>415.7	↑
Timp1	2.4<br>274.8	↑	5.8<br>270.9	↑
Serpina3n	2.8<br>679.5	↑	59.6<br>1220.2	↑
Cd68	0.8<br>94.8	↑	1.2<br>50.1	↑
Cyba	0.04<br>24.9	↑	12.1<br>85.1	↑
Aif1	0.0<br>12.9	↑	1.1<br>12.8	↑
Ctsh	0.04<br>23.0	↑	4.5<br>33.2	↑
Lgals3	0.1<br>85.8	↑	36.5<br>224.9	↑
S100a6	0.8<br>243.6	↑	289.6<br>1762.5	↑
C4b	0.2<br>118.8	↑	40.4<br>113.7	↑
Osmr	0.2<br>30.5	↑	2.1<br>19.2	↑
Anxa3	0.4<br>23.3	↑	2.6<br>65.3	↑
Hspb1	3.3<br>308.9	↑	76.8<br>872.8	↑
Rab7b	0.2<br>13.9	↑	1.4<br>15.5	↑
Capg	0.3<br>28.8	↑	19.9<br>106.2	↑
Unc93b1	0.4<br>20.6	↑	2.6<br>22.8	↑
Vgf	0.2<br>10.1	↑	2.2<br>13.8	↑
Gfap	10.4<br>3404.3	↑	5173.0<br>6064.0	↑
Tgfb1	0.9<br>22.6	↑	0.9<br>11.2	↑
Cxcl10	1.1<br>14.4	↑	4.5<br>94.0	↑
Gpsm3	0.4<br>21.0	↑	2.4<br>10.9	↑
Emp3	2.3<br>89.7	↑	13.3<br>79.7	↑
Tgfbi	0.8<br>12.6	↑	1.6<br>19.0	↑
Spp1	6.7<br>160.0	↑	16.1<br>109.5	↑
Crip1	0.9<br>12.6	↑	11.5<br>101.3	↑
Gap43	15.6<br>145.1	↑	24.7<br>156.2	↑
Fn1	12.1<br>46.0	↑	1.0<br>13.9	↑
Tagln2	21.9<br>65.9	↑	10.4<br>88.9	↑
Lcn2	2.1<br>6738.8	↑	1.2<br>678.5	↑

EMT Genes

Gene	NPC		Astrocytes	
Ptx3	0.2<br>443.8	↑	2.4<br>72.7	↑
Postn	0.1<br>14.9	↑	0.3<br>24.8	↑
Fmod	0.2<br>133.2	↑	6.9<br>109.6	↑
Mgp	0.5<br>230.4	↑	4.0<br>78.7	↑
Timp1	2.4<br>274.8	↑	5.8<br>270.9	↑
Col3A1	0.4<br>121.7	↑	3.5<br>49.5	↑
Col1A1	0.6<br>171.0	↑	4.7<br>71.9	↑
Dcn	0.2<br>76.7	↑	2.1<br>14.1	↑
Mfap5	0.01<br>11.9	↑	8.4<br>14.0	↑
Ecm1	1.5<br>105.3	↑	3.0<br>65.6	↑
Col6A2	5.6<br>118.7	↑	0.7<br>35.0	↑
Col1A2	0.1<br>36.5	↑	25.8<br>58.0	↑
Rgs4	0.1<br>11.7	↑	2.3<br>10.3	↑
Capg	0.3<br>28.8	↑	19.9<br>106.2	↑
Bgn	0.8<br>116.6	↑	24.6<br>71.6	↑
Tgfb1	0.9<br>22.6	↑	0.9<br>11.2	↑
Mylk	0.4<br>21.7	↑	3.8<br>19.3	↑
Emp3	2.3<br>89.7	↑	13.3<br>79.7	↑
Cd59A	0.3<br>32.7	↑	22.4<br>38.2	↑
Tagln	0.6<br>23.7	↑	18.1<br>90.5	↑
Tgfbi	0.8<br>12.6	↑	1.6<br>19.0	↑
Spp1	6.7<br>160.0	↑	16.1<br>109.5	↑
Sfrp4	0.6<br>11.7	↑	2.1<br>10.8	↑
Aplp1	4.3<br>206.0	↑	587.6<br>1022.5	↑
Loxl2	0.6<br>12.0	↑	4.3<br>15.7	↑
Pmepa1	1.3<br>40.6	↑	19.8<br>37.6	↑
Fn1	12.1<br>46.0	↑	1.0<br>13.9	↑
Eno2	0.7<br>25.0	↑	16.0<br>19.1	↑
Fas	3.3<br>26.6	↑	4.4<br>16.9	↑
Gadd45B	1.9<br>33.6	↑	21.2<br>28.2	↑
Pdlim4	7.4<br>86.7	↑	79.1<br>155.7	↑
Vcam1	1.1<br>27.4	↑	29.9<br>23.7	↑
Gadd45A	9.7<br>32.1	↑	5.3<br>28.3	↑
Eln	10.1<br>17.5	↑	3.3<br>27.4	↑
Ccn1	9.7<br>29.0	↑	11.5<br>54.7	↑
Tgm2	5.1<br>37.2	↑	8.0<br>15.5	↑
Sat1	15.4<br>108.3	↑	64.3<br>121.6	↑
Vim	600.4<br>1178.9	↑	163.3<br>1096.2	↑
Ntm	0.6<br>18.7	↑	175.9<br>62.4	↓
Col16A1	10.5<br>22.9	↑	7.0<br>36.3	↑
Dpysl3	12.7<br>47.7	↑	4.2<br>11.2	↑
Thy1	12.6<br>64.6	↑	103.8<br>189.2	↑
Slc6A8	10.0<br>64.5	↑	58.2<br>81.2	↑
Mmp14	46.9<br>222.6	↑	61.8<br>115.2	↑
Myl9	11.3<br>45.8	↑	17.1<br>35.5	↑
Timp3	4.1<br>54.8	↑	119.5<br>70.4	↓
Fuca1	10.4<br>32.4	↑	30.3<br>67.6	↑
Mmp2	115.2<br>60.1	↓	1.3<br>16.6	↑
Itgb5	5.9<br>37.0	↑	44.5<br>44.2	-
Acta2	0.7<br>18.4	↑	1.2<br>6.8	↑

Progenitor Genes

Gene	NPC		Astrocytes	
Ube2c	353.2<br>11.9	↓	0.7<br>11.6	↑
Cdk1	99.8<br>4.1	↓	0.2<br>3.5	↑
Kif2c	47.2<br>2.0	↓	0.1<br>0.8	↑
Cenpa	80.7<br>5.0	↓	0.4<br>4.2	↑
Top2A	97.1<br>6.3	↓	0.3<br>3.2	↑
Pou3f1	239.2<br>10.7	↓	3.6<br>22.4	↑
Mki67	19.7<br>2.0	↓	0.1<br>1.0	↑
Lhx2	25.8<br>2.7	↓	0.02<br>0.1	↑
Igfbp3	624.6<br>55.6	↓	2.7<br>17.7	↑
Ccnd1	543.2<br>77.4	↓	11.9<br>81.4	↑
Nes	193.0<br>11.3	↓	4.3<br>15.1	↑
Cenpf	11.8<br>1.6	↓	0.1<br>0.6	↑
Lgals1	1642.9<br>313.6	↓	103.3<br>465.1	↑
Cenpk	12.9<br>0.6	↓	0.2<br>0.5	↑
Tnc	79.5<br>6.2	↓	4.4<br>10.0	↑
Sox11	25.3<br>2.6	↓	0.6<br>1.3	↑
Traf4	119.7<br>27.1	↓	5.8<br>14.3	↑
Efnb1	18.3<br>5.0	↓	2.2<br>5.5	↑
Ccnd2	157.6<br>19.7	↓	24.0<br>34.5	↑
Igfbp2	2878.8<br>81.8	↓	85.5<br>97.9	↑
Hjurp	2.1<br>0.6	↓	0.3<br>0.3	↑
Wnt5a	11.5<br>5.2	↓	2.9<br>3.8	↑
Metrn	129.8<br>106.2	↓	173.3<br>142.9	↓
Slc1a3	177.9<br>166.9	↓	161.2<br>67.3	↓
Zic1	13.3<br>9.3	↓	4.6<br>3.1	↓
Bmi1	25.6<br>8.2	↓	8.7<br>7.6	↓
Serpine2	1071.8<br>549.7	↓	595.0<br>449.3	↓
Fzd9	113.0<br>44.4	↓	24.5<br>14.8	↓
Fzd1	60.5<br>23.3	↓	27.3<br>13.8	↓
Igfbp4	1169.2<br>294.1	↓	127.8<br>69.6	↓
Nptx1	90.9<br>3.1	↓	1.8<br>1.1	↓
Lmo3	9.9<br>1.0	↓	3.8<br>1.6	↓

Key

Gene	NPC		Astrocytes	
Gene Symbol	NPC (Pre-graft) FPKM<br>NPC in SCI FPKM	↓ = DOWN regulated in SCI condition	Astros (Healthy) FPKM<br>Astros in SCI FPKM	↑ = UP regulated in SCI condition

Supplementary Fig. 14 | Gene expression levels by FPKM values for select astrocyte state genes (healthy, reactivity and EMT) and progenitor genes for NPC grafted into SCI compared with expression levels for host astrocytes in healthy and SCI. Red upward arrows (↑) indicates an UP-regulation of expression for that gene in SCI while a blue downward arow (↓) indicates DOWN-regulated expression of that specific gene. Analysis of FPKM values reveals convergent changes in healthy astrocyte genes, congruent changes in reactivity and EMT genes.

Astrocyte Neural tissue support function genes

	Gene	NPC		Astrocytes	
Synapse Maintenance	Sparcl1	100.8<br>717.1	↑	4116.2<br>1128.2	↓
	Sparc	564.4<br>1066.1	↑	1729.4<br>1238.8	↓
	Gpc5	0.5<br>0.7	↑	70.2<br>13.5	↓
	Grm3	0.8<br>0.8	-	7.0<br>1.5	↓
	Grin2c	0.4<br>0.2	↓	17.9<br>3.1	↓
Channels	Kcnj10	12.5<br>148.9	↑	797.1<br>347.2	↓
	Kcnj16	0.2<br>9.2	↑	140.3<br>35.5	↓
	Gja1	54.2<br>324.4	↑	581.2<br>213.4	↓
	Gjb6	0.1<br>32.1	↑	291.8<br>80.5	↓
Astrocyte enriched Transporters	Atp1a2	2.4<br>398.0	↑	2315.2<br>872.8	↓
	Dio2	0.1<br>2.6	↑	17.7<br>3.8	↓
	Slc1a2	1.7<br>85.3	↑	851.2<br>271.8	↓
	Slc4a4	1.8<br>42.9	↑	216.0<br>80.9	↓
	Slc6a1	13.7<br>42.3	↑	418.6<br>98.5	↓
	Slc6a11	1.8<br>55.8	↑	763.1<br>117.8	↓
	Slc7a10	0.3<br>3.3	↑	247.5<br>45.3	↓
	Slc13a5	0.2<br>1.5	↑	8.0<br>1.5	↓
	Slc25a18	65.8<br>163.0	↑	383.0<br>185.4	↓
	Slc27a1	35.7<br>45.6	↑	125.4<br>50.9	↓
	Slc39a12	0.4<br>29.8	↑	220.1<br>70.5	↓

Key

Gene	NPC		Astrocytes	
Gene Symbol	NPC (Pre-graft) FPKM<br>NPC in SCI FPKM	↓ = **DOWN** regulated in SCI condition	Astros (Healthy) FPKM<br>Astros in SCI FPKM	↑ = **UP** regulated in SCI condition

Supplementary Fig. 15 | Changes after SCI in gene expression levels by FPKM values for select healthy astrocyte genes associated with specialized neural tissue support functions for grafted NPC and host astrocytes. Red upward arrows (↑) indicates an UP-regulation of expression for that gene in SCI while a blue downward arow (↓) indicates DOWN-regulated expression of that specific gene. There is reduced expression of genes associated with specialized neuronal support functions in host astrocytes following SCI. There is generally increased expression of these same genes in NPC upon grafting into SCI however the FPKM levels for all genes failed to reach the levels of host astrocytes in either uninjured or SCI.

Top 50 healthy astrocyte genes upregulated by NPC at least $Log_2FC>5$ upon grafting that were not altered by tissue environment

Healthy Astrocyte Genes	DEG Log2FC relative to Pre-graft NPC		
	Uninjured	SCI	dSCI
Pm20d1	11.6	10.9	12.2
Ucp3	11.1	9.7	10.6
Etnppl	11.1	9.8	10.6
Fam107a	9.4	10.0	9.3
Cyp2d22	9.2	8.6	9.3
Lgi4	9.2	9.6	9.2
Itih3	9.2	9.3	8.6
**Gjb6**	**8.8**	**9.0**	**9.4**
Ntsr2	8.7	9.2	7.8
Aldh1a1	8.7	9.0	8.6
**Aqp4**	**8.6**	**9.9**	**9.7**
Ddo	8.5	9.3	8.6
C4b	8.4	9.3	9.0
Paqr6	8.4	7.9	7.9
Slc14a1	8.3	9.1	8.6
Entpd2	8.2	8.7	9.0
Padi2	8.0	8.5	7.5
Hsd11b1	7.8	6.6	7.6
**Gfap**	**7.8**	**8.4**	**7.4**
Baalc	7.8	6.9	7.1
Gpr37l1	7.7	8.4	7.2
Slc25a34	7.4	7.5	7.3
S100a1	7.4	7.5	7.8
Bbox1	7.2	7.7	7.2
**Atp1a2**	**7.0**	**7.5**	**6.9**
D630039A03Rik	7.0	6.7	6.3
Psd2	7.0	6.4	6.4
**Pla2g7**	**7.0**	**7.8**	**7.1**
4932438H23Rik	6.8	7.1	6.3
Atp13a4	6.7	6.1	5.8
Tmem100	6.7	7.1	6.5
Dapp1	6.7	7.5	8.0
**Aqp9**	**6.7**	**6.9**	**6.9**
**S100b**	**6.7**	**6.8**	**6.1**
Myh15	6.5	6.3	5.5
Sfrp5	6.5	6.4	7.1
Maob	6.5	5.6	6.7
Elf5	6.4	6.4	6.7
Clu	6.3	6.9	6.4
Kcnj16	6.1	5.7	5.2
Slc39a12	6.1	6.2	5.9
Lrrc2	6.1	5.9	6.1
P4ha3	6.1	6.7	6.3
Slc1a2	6.0	5.8	5.8
Hepacam	6.0	6.7	5.9
Pdk4	5.9	5.5	6.2
Scg3	5.8	6.3	5.8
Echdc3	5.8	5.6	6.3
Gm973	5.4	5.6	5.1
Atp1b2	5.3	5.8	5.0

Supplementary Table 4 | Healthy astrocyte genes upregulated in NPC upon grafting not altered significantly by tissue environment.

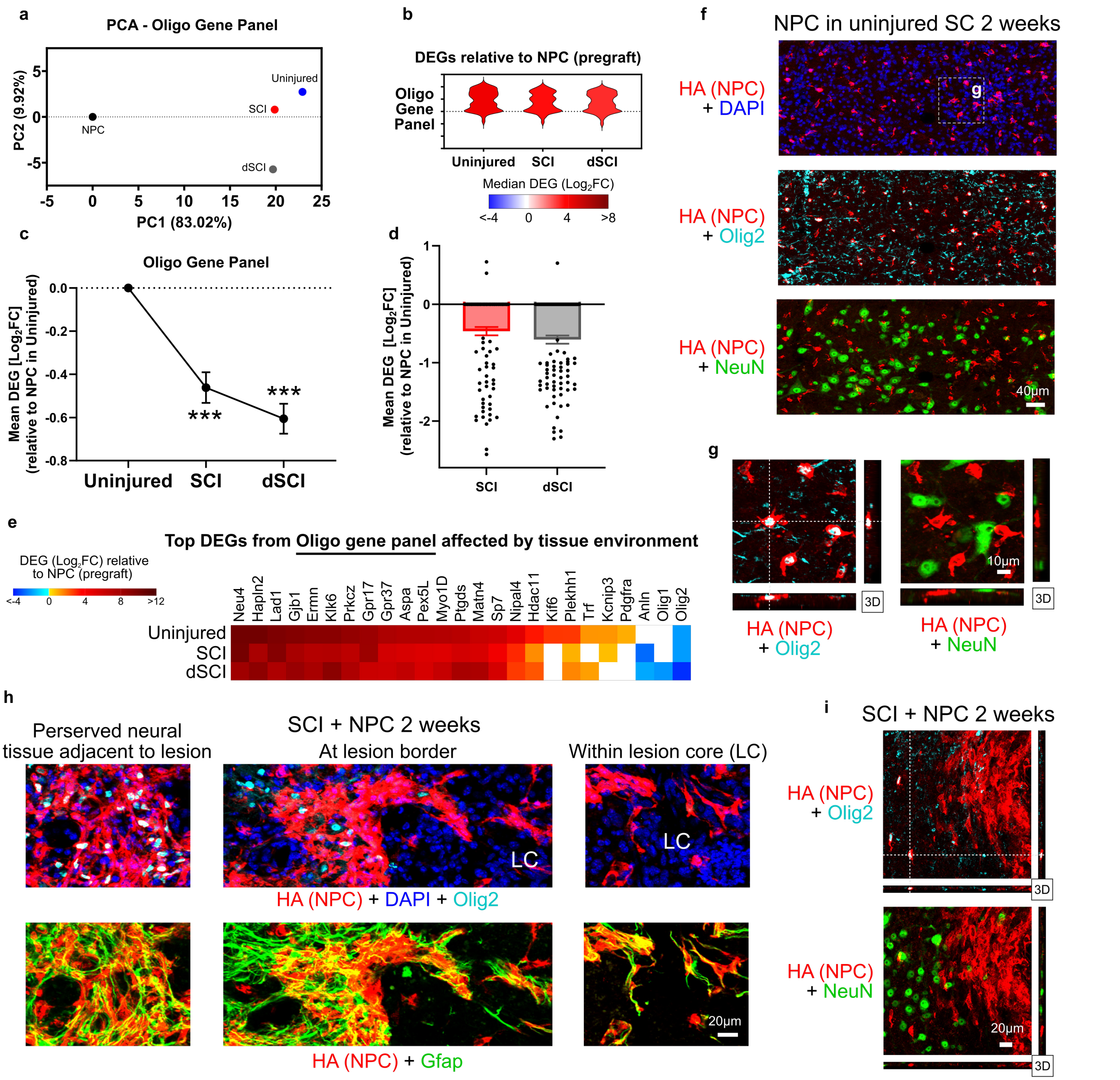

Supplementary Fig. 16 | NPC grafted into uninjured spinal cord tissue show an elevated oligodendroglia transcriptional profile compared to grafts made into SCI lesions. a PCA of oligodendroglia gene panels for NPC grafted into different tissue environments (uninjured, SCI and double crush SCI (dSCI)). (N=5 mice per group). **b** Violin plots of DEGs for NPC grafted into different tissue environments for the Oligodendroglia gene panel. **c** Plot of Mean DEGs for Oligodendroglia gene panel for NPC grafts recovered from SCI and dSCI referenced to NPC grafts recovered from uninjured spinal cord tissue. Graph shows mean ± s.e.m. **d** Mean DEGs for Oligodendroglia gene panel for NPC grafts recovered from SCI and dSCI referenced to NPC grafts recovered from uninjured spinal cord tissue showing distribution of individual DEGs as overlayed points. Graph shows mean ± s.e.m. For c, d (N=5 mice per group) *** p-value < 0.0001, NS = not significant, One-way ANOVA with Tukey multiple comparison test. **e** Heat map of top DEGs from oligodendroglia gene panel altered by tissue environment. **f** Survey IHC image of NPC grafted into uninjured healthy spinal cord. **g** Detailed image 3D IHC image of NPC grafts in uninjured healthy spinal cord showing NPC progeny that are Olig2 positive OPC-like cells. **h** Survey IHC images showing NPC within the three compartments of SCI lesions. Olig2 grafts were found in volumes of preserved neural tissue but not within the lesion. **i** Detailed 3D IHC image showing Olig2 positive NPC in neural tissue beds (NeuN-positive) but no Olig2-postive NPC in the graft derived astroglia border. with individual datapoints overlayed.

Uncropped Western Blots

Supplementary Fig. 1h

Fabp7

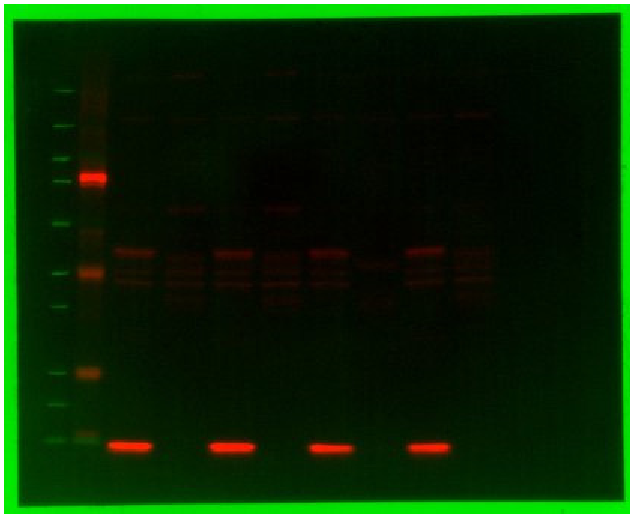

Sox9

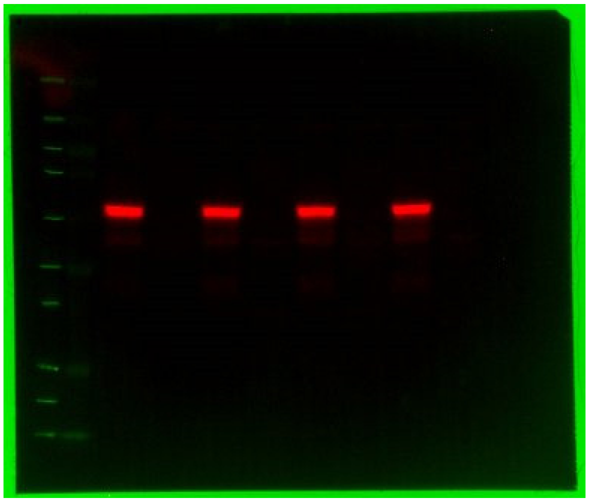

Nestin

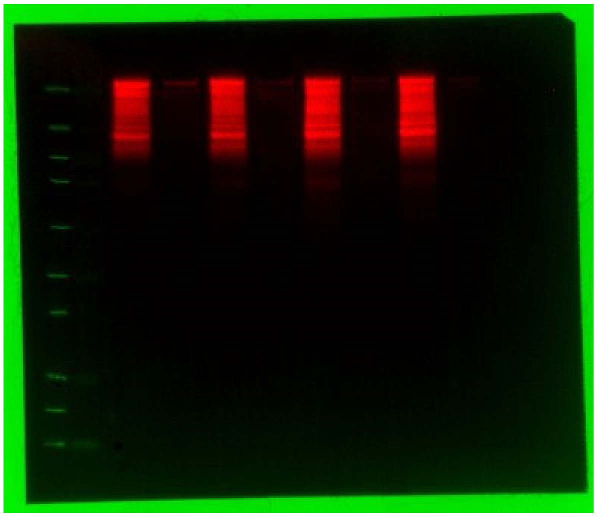

Nanog

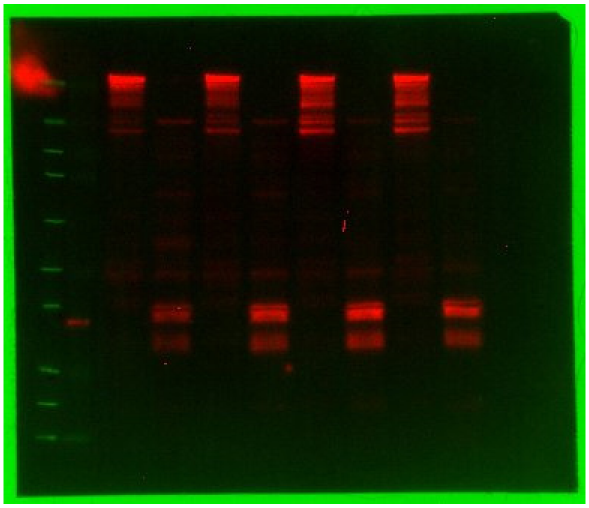

Dppa4

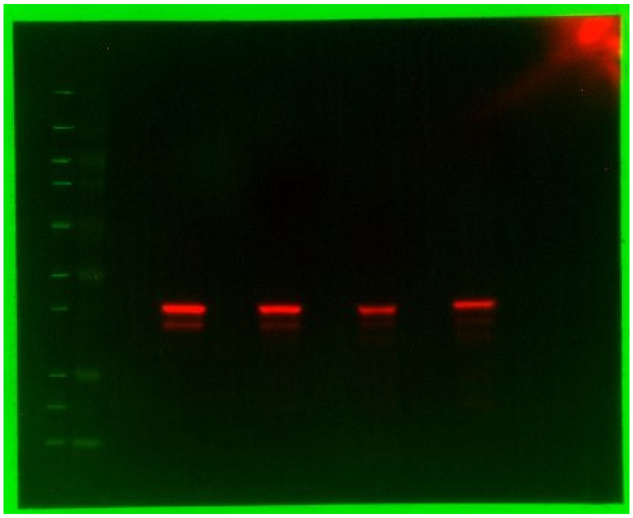

Oct4

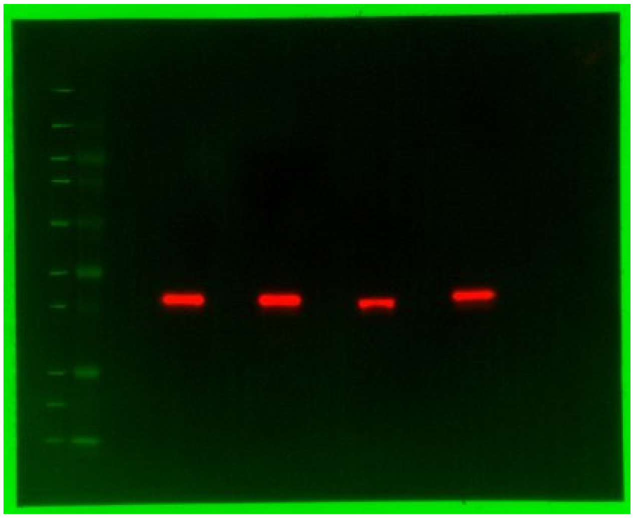

Supplementary Fig. 5f

Gfap

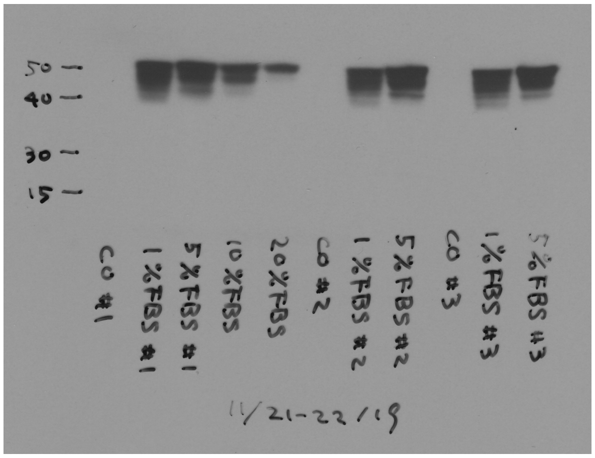

Cofilin

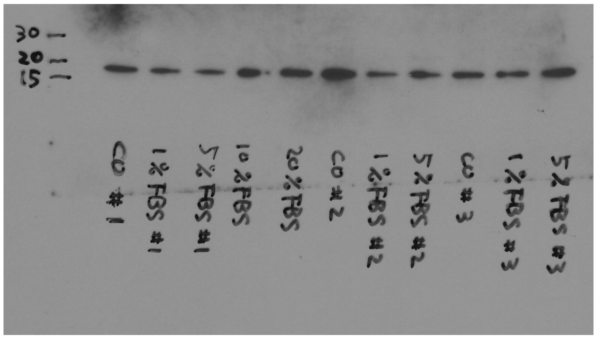

α-Sma

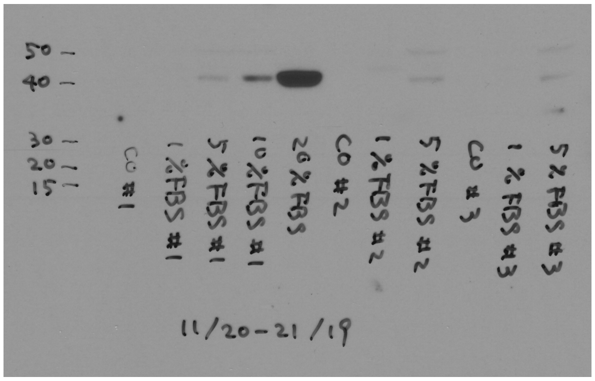

Cofilin

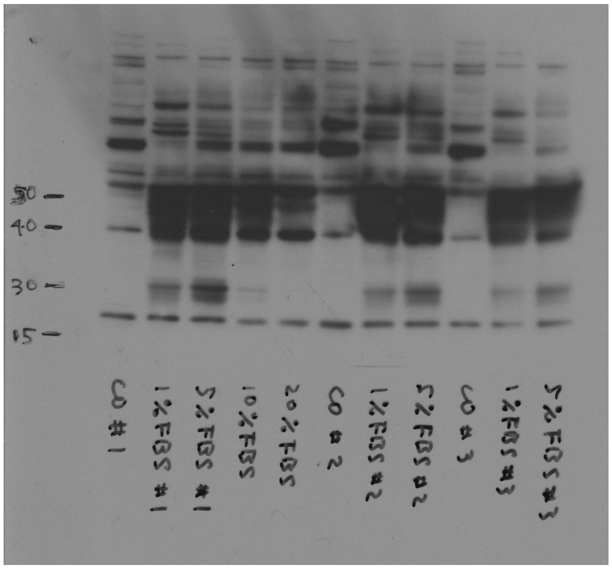

Supplementary Fig. 5h

Gfap

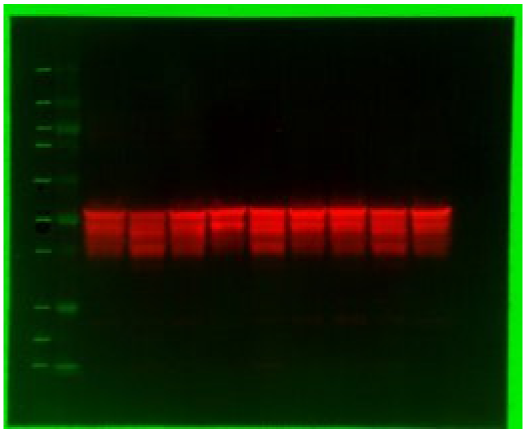

α-Sma

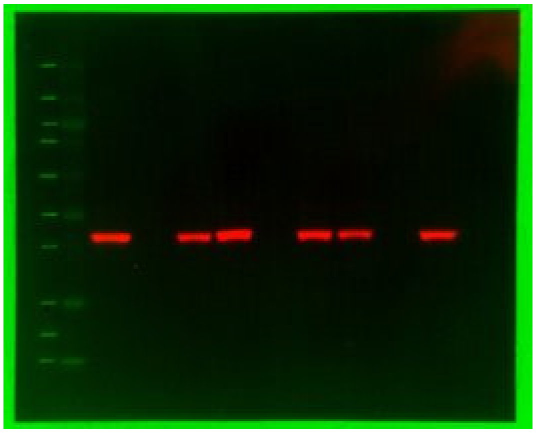

Supplementary Fig. 5i

α-Sma

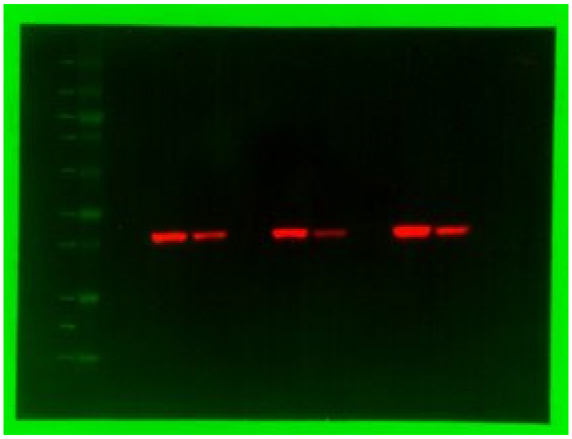

Supplementary Fig. 6h

Gfap

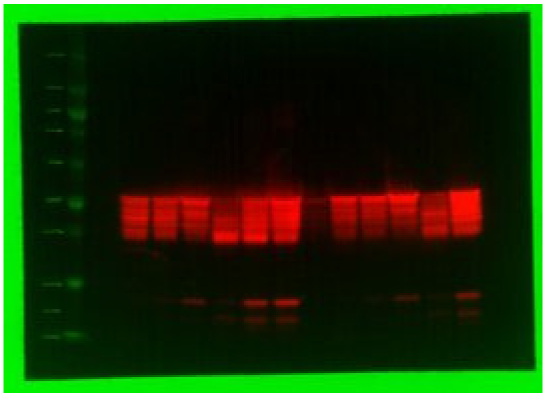

α-Sma

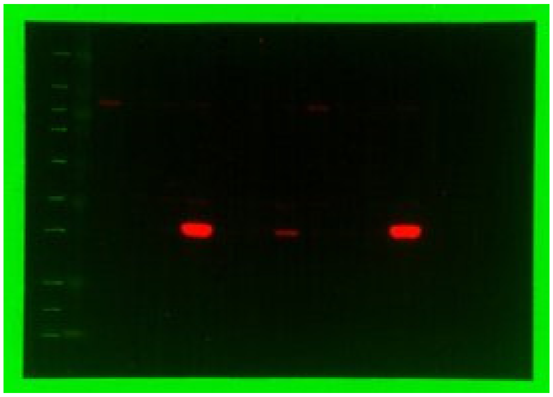

Supplement: Supplementary file 1 — Supplementary Information [file 41467_2022_33382_MOESM1_ESM.pdf]
