# Supplementary material for: Lesion environments direct transplanted neural progenitors towards a wound repair astroglial phenotype in mice
Source: Nat Commun. 2022 Sep 28;13:5702. doi: 10.1038/s41467-022-33382-x (PMC9519954; doi:10.1038/s41467-022-33382-x)
Supplement: Supplementary file 2 — Reporting Summary [file 41467_2022_33382_MOESM2_ESM.pdf]

nature portfolio

Corresponding author(s): Timothy M O'Shea and Michael V Sofroniew

Last updated by author(s): Aug 22, 2022

Reporting Summary

Nature Portfolio wishes to improve the reproducibility of the work that we publish. This form provides structure for consistency and transparency in reporting. For further information on Nature Portfolio policies, see our Editorial Policies and the Editorial Policy Checklist.

Statistics

For all statistical analyses, confirm that the following items are present in the figure legend, table legend, main text, or Methods section.

n/a	Confirmed	
☐	☒	The exact sample size (*n*) for each experimental group/condition, given as a discrete number and unit of measurement
☐	☒	A statement on whether measurements were taken from distinct samples or whether the same sample was measured repeatedly
☐	☒	The statistical test(s) used AND whether they are one- or two-sided<br>*Only common tests should be described solely by name; describe more complex techniques in the Methods section.*
☒	☐	A description of all covariates tested
☐	☒	A description of any assumptions or corrections, such as tests of normality and adjustment for multiple comparisons
☐	☒	A full description of the statistical parameters including central tendency (e.g. means) or other basic estimates (e.g. regression coefficient) AND variation (e.g. standard deviation) or associated estimates of uncertainty (e.g. confidence intervals)
☐	☒	For null hypothesis testing, the test statistic (e.g. *F*, *t*, *r*) with confidence intervals, effect sizes, degrees of freedom and *P* value noted<br>*Give P values as exact values whenever suitable.*
☒	☐	For Bayesian analysis, information on the choice of priors and Markov chain Monte Carlo settings
☒	☐	For hierarchical and complex designs, identification of the appropriate level for tests and full reporting of outcomes
☒	☐	Estimates of effect sizes (e.g. Cohen's *d*, Pearson's *r*), indicating how they were calculated

Our web collection on statistics for biologists contains articles on many of the points above.

Software and code

Policy information about availability of computer code

Data collection	Microsoft Excel for Microsoft Office 365 ProPlus, Imaris 9.2 (Bitplane), or Zen 3.1 (Blue Edition) (Zeiss), and Aperio Imagescope v12.3 (Leica) were commercially available software used in data collection.
Data analysis	Microsoft Excel for Microsoft Office 365 ProPlus, NIH Image J (1.51), G*Power Software V 3.1.9.2., Prism 9 (GraphPad Software Inc), XLStat Basic 2020.3.1 (Addinsoft Inc), Galaxy (https://usegalaxy.org/) to use the following tools - Trimmomatic (Galaxy Version.38.0), HISAT2 (Galaxy Version 2.1.0+galaxy4), featureCounts (Galaxy Version 1.6.3+galaxy2), EdgeR (Galaxy Version 3.34.0+galaxy1), ScanPy Galaxy Suite, and ChemDraw 18.2 were commercially available software used in data analysis.

For manuscripts utilizing custom algorithms or software that are central to the research but not yet described in published literature, software must be made available to editors and reviewers. We strongly encourage code deposition in a community repository (e.g. GitHub). See the Nature Portfolio guidelines for submitting code & software for further information.

Data

Policy information about availability of data

All manuscripts must include a data availability statement. This statement should provide the following information, where applicable:

- Accession codes, unique identifiers, or web links for publicly available datasets
- A description of any restrictions on data availability
- For clinical datasets or third party data, please ensure that the statement adheres to our policy

All data generated for this study are included in the main and supplementary figures. For all quantitative figures, files of source data of individual values as well as the results of statistical tests are provided with the paper. Other data that support the findings of this study are available on reasonable request from the corresponding author. RiboTag RNA-Seq and Single-cell RNA-seq data have been deposited at Gene Expression Omnibus (GEO) and are publicly available as of the

date of publication with Accession numbers 194319.

To generate the Astrocyte state gene panels used in the paper the following datasets were accessed and analyzed from the National Center for Biotechnology Information (NCBI) Gene Expression Omnibus (GEO): GSE199149, GSE94010, GSE84540, GSE114000, GSE52564, GSE18765, GSE66370, GSE103783, GSE35338, GSE153721, GSE100329.

Field-specific reporting

Please select the one below that is the best fit for your research. If you are not sure, read the appropriate sections before making your selection.

- [x] Life sciences
- [ ] Behavioural & social sciences
- [ ] Ecological, evolutionary & environmental sciences

For a reference copy of the document with all sections, see nature.com/documents/nr-reporting-summary-flat.pdf

Life sciences study design

All studies must disclose on these points even when the disclosure is negative.

Sample size	For in vivo experiments the animal group sizes were calculated to provide at least 80% power when using the following parameters: probability of type I error (alpha) = .05, a conservative effect size of 0.25, 2-5 treatment groups with multiple measurements obtained per replicate. For in vitro experiments sample sizes were calculated to provide at least 80% power when using the following parameters: probability of type I error (alpha) = .05, a conservative effect size of 0.25, 2-4 experimental groups with multiple measurements obtained per replicate. For scRNA-seq, we obtained 3 samples of the different experimental groups and were sufficient as we obtained thousands of cells in each sample. For all experiments performed as part of this paper the groups sizes are reported. For all other experiments no specific power analysis was used with samples sizes determined by following general standards of the field.
Data exclusions	No data were excluded from analysis.
Replication	In vivo experiments that involved injections of NPC formulations, stroke inducing agent L-NIO and application of SCI crush injury were repeated independently at least three times in different cohorts of mice across a three-year period with similar results. Key data generated from the immunohistochemistry analysis were repeated independently by two co-authors. In vitro cell culture studies were replicated at least three times in different cell stocks with all experiments having 3-4 independent biological replicates to ensure reproducibility. All replications were successful.
Randomization	Animals were randomly assigned numbers and thereafter were evaluated blind to experimental condition. Across all experiments animals were randomized for weight, age and sex. For in vitro studies, imaging fields of view were chosen randomly.
Blinding	Animals were randomly assigned numbers and thereafter were evaluated blind to experimental condition throughout RiboTag immunoprecipitation and RNA extraction, immunohistochemical processing and imaging. For in vitro studies, western blotting, RNA extraction, immunohistochemical processing and imaging processing were performed blinded to experimental condition.

Reporting for specific materials, systems and methods

We require information from authors about some types of materials, experimental systems and methods used in many studies. Here, indicate whether each material, system or method listed is relevant to your study. If you are not sure if a list item applies to your research, read the appropriate section before selecting a response.

Materials & experimental systems

n/a	Involved in the study	
[ ]	[x]	Antibodies
[ ]	[x]	Eukaryotic cell lines
[x]	[ ]	Palaeontology and archaeology
[ ]	[x]	Animals and other organisms
[x]	[ ]	Human research participants
[x]	[ ]	Clinical data
[x]	[ ]	Dual use research of concern

Methods

n/a	Involved in the study	
[x]	[ ]	ChIP-seq
[x]	[ ]	Flow cytometry
[x]	[ ]	MRI-based neuroimaging

Antibodies

Antibodies used	The primary antibodies used in this study were: rabbit Hemagglutinin (HA) (1:1000, Sigma #H6908); goat HA (1:800, Novus, NB600-362); rabbit alpha-smooth muscle actin (α-Sma) (1:200, Novus, NB600-531); rabbit anti-Gfap (1:1000, Dako/Agilent, GA524); rat anti-Gfap (1:1000, Thermofisher, #13-0300); rabbit anti NeuN (1:1000, Abcam, ab177487); guinea pig anti-NeuN (1:1000, Synaptic Systems, 266 004); goat anti-Cd13 (1:200, R&D systems, AF2335); rat anti-Galectin-3 (1:200, Invitrogen, 14-5301-82); rabbit anti-Fibronectin (1:500, Millipore, Cat#AB2033); rat anti-Cd68 (1:1000, AbDserotec-BioRad, MCA1957); rabbit anti-Iba-1 (1:800, Wako, 019-19741); guinea pig anti-Iba-1 (1:800, Synaptic systems, 234 004); rabbit anti-P2ry12 (1:500, Anaspec, AS-55043A); goat anti-Pdgfr-α (1:500, R&D systems, AF1062); goat anti-Nestin (1:500, R&D, AF2736); goat anti-Oct4 (1:500, R&D systems, AF1759);

goat anti-Sox9 (1:500, R&D systems, AF3075); rabbit Aldh1l1 (1:1000, Abcam, Ab87117); rabbit anti-Amyloid Beta (Aβ) (1:200, Abcam, Ab2539); rabbit anti-Amyloid precursor protein (App) (1:200, abcam, ab32136); goat anti-Carboxypeptidase E/CPE (Cpe) (1:200, R&D systems, AF3587); goat anti-Lipocalin-2 (Lcn2) (1:200, R&D systems, AF1857), goat anti-Clusterin (Clu) (1:200, R&D systems, AF2747); Rabbit anti-Tuj-1 (1:500, Sigma, T2200-200UL); rat anti-Vimentin (1:500; R&D Systems, MAB2105); rat anti-Cd44 (IM7) (1:200; Thermofisher Scientific, #14-0441-82); goat anti-Dppa4 (1:200; R&D Systems, AF3730), Rabbit anti-Id3 (1:200; Cell Signaling Technology, #9837); rabbit anti–HSV-TK (1:1000) (generated by M. Sofroniew and validated previously in Bush et al. 1999. Neuron.) Secondary antibodies used were goat anti-rabbit (1:10000, A27036, ThermoFisher); donkey anti-goat (1:5000, A15999, ThermoFisher); goat anti-mouse (1:20000, Cat#62-6520, ThermoFisher).

Validation

All antibodies used were sourced from commercial vendors and were selected because they had previously been validated for use on mouse tissue (validated mouse reactivity) and for use in fluorescent immunohistochemistry (IHC) applications. Furthermore, validation of these antibodies can be found in peer reviewed publications by our team and others which are referenced throughout the manuscript. Additional validation information of each antibody is available from the various manufacturers' websites and validation information for each individual antibody is provided below.

Rabbit anti-GFAP (1:1000; Cat#Z-0334, Dako, Santa Clara, CA) validated and used consistently over many publications (e.g. (Anderson et al. Nature. 2016 & 2018), validated by Dako and information available on their website, used extensively across many other publications (~1839 citations on CiteAb);

Rat anti-GFAP (1:1000, Cat#13-0300, Thermofisher, Grand Island, NY) validated by company on their website and used within the concentration range recommended for IHC, cross-validated by us by comparing with the Rabbit GFAP above;

Rabbit anti NeuN (1:1000, Cat#Ab177487, Abcam, Cambridge, MA) - information describing validation of concentration and for use with mouse on company website, used at recommended concentration for IHC by manufacturer and used by us extensively in previous studies (O'Shea et al. Nature Communications. 2020);

Guinea pig anti-NeuN (1:1000, Cat#266-004, Synaptic Systems, Goettingen, Germany) validation information on company website, and our own successful validation was made by comparing with Rabbit anti-NeuN above.

Goat anti-CD13 (1:200, Cat#AF2335, R&D systems, Minneapolis, MN) - validation information on company website and used by us at recommended concentration range in previous studies (e.g. O'Shea et al. Nature Communications. 2020);

rabbit anti-Fibronectin (1:500, Cat#AB2033, Millipore, Burlington, MA) - validation information on company website and used by us extensively in previous studies (e.g. Anderson et al. Nature. 2018);

Rat anti-Galectin-3 (1:200, Cat#14-5301-82 Invitrogen-Thermofisher Scientific, Grand Island, NY)- validation information on company website, manufacturer states that "Antibody was verified by Knockdown to ensure that the antibody binds to the antigen stated" and used at the specified concentration for IHC provided by the manufacturers;

Rat anti-CD68 (1:1000, Cat# MCA1957, AbDserotec-BioRad, Hercules, CA)-validation information on company website and used previously by us in publications;

Rabbit anti-Iba-1 (1:800, Cat#019-19741, Wako, Osaka, Japan)-validation information on company website and used previously by us in publications;

Guinea pig anti-Iba-1 (1:800, Cat#234-004, Synaptic systems, Goettingen, Germany)-validation information on company website, staining profile was compared with the Rabbit anti-Iba-1 to confirm specificity;

Rabbit anti-P2Y12R (1:500, Cat#AS-55043A, Anaspec, Fremont, CA)-validation information on company website, used within recommended concentration, staining compared with Iba-1 in uninjured mouse brain tissue to confirm specificity for microglia;

Rabbit Hemagglutinin (HA) (1:1000, Sigma #H6908) validation information on company website, used within recommended concentration, staining compared with previous studies (e.g. Anderson et al. Nature. 2016);

Goat HA (1:800, Novus, NB600-362) validation information on company website, used within recommended concentration, staining compared with Rabbit HA above;

Rabbit alpha-smooth muscle actin (α-Sma) (1:200, Novus, NB600-531) validation information on company website, used within recommended concentration;

goat anti-Pdgfr-α (1:500, R&D systems, AF1062) validation information on company website, used within recommended concentration;

goat anti-Nestin (1:500, R&D, AF2736) validation information on company website, used within recommended concentration, used previously by us (Wollenberg et al. 2018. Biomaterials).

goat anti-Oct4 (1:500, R&D systems, AF1759) validation information on company website, used within recommended concentration;

goat anti-Sox9 (1:500, R&D systems, AF3075) information describing validation of concentration and for use with mouse on company website, used at recommended concentration for IHC by manufacturer and used by us extensively in previous studies (Ren et al. Scientific Reports. 2017);

rabbit Aldh1l1 (1:1000, Abcam, Ab87117) information describing validation of concentration and for use with mouse on company website, used at recommended concentration for IHC by manufacturer and used by us extensively in previous studies (Ren et al. Scientific Reports. 2017);;

rabbit anti-Amyloid Beta (Aβ) (1:200, Abcam, Ab2539) information describing validation of concentration and for use with mouse on company website, used at recommended concentration for IHC by manufacturer and used by us extensively in previous studies (O'Shea et al. Nature Communications. 2020);

rabbit anti-Amyloid precursor protein (App) (1:200, abcam, ab32136) information describing validation of concentration and for use with mouse on company website, used at recommended concentration for IHC by manufacturer and used by us extensively in previous studies (O'Shea et al. Nature Communications. 2020);

goat anti-Carboxypeptidase E/CPE (Cpe) (1:200, R&D systems, AF3587) information describing validation of concentration and for use with mouse on company website, used at recommended concentration for IHC by manufacturer;

goat anti-Lipocalin-2 (Lcn2) (1:200, R&D systems, AF1857) information describing validation of concentration and for use with mouse on company website, used at recommended concentration for IHC by manufacturer;

goat anti-Clusterin (Clu) (1:200, R&D systems, AF2747) information describing validation of concentration and for use with mouse on company website, used at recommended concentration for IHC by manufacturer;

Rabbit anti-Tuj-1 (1:500, Sigma, T2200-200UL) validation information on company website, used within recommended concentration, used previously by us (Wollenberg et al. 2018. Biomaterials).

rat anti-Vimentin (1:500; R&D Systems, MAB2105) information describing validation of concentration and for use with mouse on company website, used at recommended concentration for IHC by manufacturer;

rat anti-Cd44 (IM7) (1:200; Thermofisher Scientific, #14-0441-82) information describing validation of concentration and for use with

mouse on company website, used at recommended concentration for IHC by manufacturer;
goat anti-Dppa4 (1:200; R&D Systems, AF3730) information describing validation of concentration and for use with mouse on company website, used at recommended concentration for IHC by manufacturer
Rabbit anti-Id3 (1:200; Cell Signaling Technology, #9837) information describing validation of concentration and for use with mouse on company website, used at recommended concentration for IHC by manufacturer.

Eukaryotic cell lines

Policy information about cell lines

Cell line source(s)	Mouse embryonic stem cells (mESC) were derived by certified personnel at Jackson Labs from the inner cell mass of E3.5 blastocyst stage embryos generated from crosses of male homozygous B6N.129-Rpl22tm1.1 Psam/J(RRID: IMSR_JAX: Stock No: 011029) "Ribotag" mice to females hemizygous for a dominant, maternal effect cre allele, B6.Cg-Tg(SOX2-cre)1Amc/J (RRID: IMSR_JAX: 008454) and heterozygous for the "RiboTag" allele. Multiple male and female mESC lines were derived, and each was karyotyped and genotyped to confirm sex and homozygosity for the cre-exised, Ribotag allele
Authentication	Karyotyped and genotyped
Mycoplasma contamination	Derived NPC were tested using Mouse/Rat Comprehensive CLEAR Panel (perfromed by Charles River Research Animal Diagnostic Services) to screen for infectious diseases via PCR. Cells were negative for mycoplasma and all other rodent viruses by this testing.
Commonly misidentified lines (See ICLAC register)	N/A

Animals and other organisms

Policy information about studies involving animals; ARRIVE guidelines recommended for reporting animal research

Laboratory animals	All in vivo animal experiments were conducted within approved UCLA facilities using wildtype or transgenic C57/BL6 female and male mice that were aged between 8 weeks and four months old at the time of craniotomy or spinal cord injury surgery. B6N.129-Rpl22tm1.1Psam/J (RRID: IMSR_JAX: 011029) were bred with B6.Cg-Tg(Gfap-cre)73.12 Mvs/J (RRID: IMSR_JAX: 012886) from an in-house colony to generate Transgenic 73.12 GFAP Cre -RiboTag mice. B6.Cg-Tg(Gfap-TK)7.1Mvs/J (RRID: IMSR_JAX: 005698) were bred from an in-house colony to generate GFAP-TK mice. Mice were housed in a 12-hour light/dark cycle in a specific pathogen-free facility with controlled temperature (20°C-25°C) and humidity (50-70%) and were provided with food and water ad libitum.
Wild animals	No wild animals were used in the study.
Field-collected samples	No field collected samples were used in the study.
Ethics oversight	All in vivo experiments involving the use of mice were conducted according to protocols approved by the Animal Research Committee (ARC) of the Office for Protection of Research Subjects at University of California Los Angeles (UCLA).

Note that full information on the approval of the study protocol must also be provided in the manuscript.
